# Synthesis and Evaluation of Artificial Nucleic Acid Bearing an Oxanorbornane Scaffold

**DOI:** 10.3390/molecules25071732

**Published:** 2020-04-09

**Authors:** Hibiki Komine, Shohei Mori, Kunihiko Morihiro, Kenta Ishida, Takumi Okuda, Yuuya Kasahara, Hiroshi Aoyama, Takao Yamaguchi, Satoshi Obika

**Affiliations:** 1 Graduate School of Pharmaceutical Sciences, Osaka University, 1-6 Yamadaoka, Suita, Osaka 565-0871, Japan; flyfly113@gmail.com (H.K.); s7592mori@gmail.com (S.M.); kenta.ishida0918@gmail.com (K.I.); taku0903taku@gmail.com (T.O.); y-kasahara@nibiohn.go.jp (Y.K.); haoyama@phs.osaka-u.ac.jp (H.A.); 2 National Institutes of Biomedical Innovation, Health and Nutrition (NIBIOHN), 7-6-8 Saito-Asagi, Ibaraki, Osaka 567-0085, Japan

**Keywords:** modified nucleic acid, modified oligonucleotide, oxanorbornane, conformational restriction, entropy, duplex-forming ability, mismatch discrimination, nuclease resistance

## Abstract

Natural oligonucleotides have many rotatable single bonds, and thus their structures are inherently flexible. Structural flexibility leads to an entropic loss when unwound oligonucleotides form a duplex with single-stranded DNA or RNA. An effective approach to reduce such entropic loss in the duplex-formation is the conformational restriction of the flexible phosphodiester linkage and/or sugar moiety. We here report the synthesis and biophysical properties of a novel artificial nucleic acid bearing an oxanorbornane scaffold (OxNorNA), where the adamant oxanorbornane was expected to rigidify the structures of both the linkage and sugar parts of nucleic acid. OxNorNA phosphoramidite with a uracil (U) nucleobase was successfully synthesized over 15 steps from a known sugar-derived cyclopentene. Thereafter, the given phosphoramidite was incorporated into the designed oligonucleotides. Thermal denaturation experiments revealed that oligonucleotides modified with the conformationally restricted OxNorNA-U properly form a duplex with the complementally DNA or RNA strands, although the *T*_m_ values of OxNorNA-U-modified oligonucleotides were lower than those of the corresponding natural oligonucleotides. As we had designed, entropic loss during the duplex-formation was reduced by the OxNorNA modification. Moreover, the OxNorNA-U-modified oligonucleotide was confirmed to have extremely high stability against 3′-exonuclease activity, and its stability was even higher than those of the phosphorothioate-modified counterparts (*S*p and *R*p). With the overall biophysical properties of OxNorNA-U, we expect that OxNorNA could be used for specialized applications, such as conformational fixation and/or bio-stability enhancement of therapeutic oligonucleotides (e.g., aptamers).

## 1. Introduction

**Figure 1:**
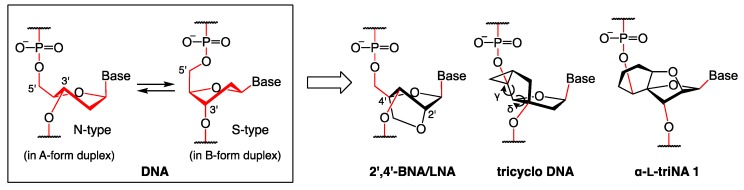
Conformational restrictions of natural oligonucleotides.

The structural flexibility of natural oligonucleotides contributes to the formation of various higher-order structures. However, when unwound oligonucleotides form a specific structure (e.g., duplex), a large entropic loss generally arises. Thus, chemical modifications that restrict the structures of flexible phosphodiester linkages and/or sugar moieties to those seen in A-form DNA·RNA duplexes have a useful role in producing high-affinity (i.e., potent) antisense oligonucleotides [1,2,3]. In our laboratory, a number of conformationally restricted artificial nucleic acids, for which a representative example is 2′,4′-bridged nucleic acid (2′,4′-BNA, commonly called locked nucleic acid (LNA)) (Figure 1), have been developed [4,5,6,7,8]. 2′,4′-BNA/LNA has an N-type sugar pucker commonly seen in the A-form duplex, and thus the 2′,4′-BNA/LNA-modified oligonucleotides exhibit a high duplex-forming ability toward single-stranded RNA (ssRNA). Leumann and co-workers have demonstrated that oligonucleotides modified with tricyclo-DNA, in which a fused ring system rigidifies the torsion angles γ and δ, exhibit increased ssRNA affinity relative to the natural oligonucleotides [9,10,11,12]. More recently, Henessian and co-workers have successfully achieved restrictions of both the sugar ring and the torsion angle γ by designing α-l-triNA 1 [13]. Oligonucleotides containing α-l-triNA 1 are known to have a high binding affinity toward ssRNA.

**Figure 2:**
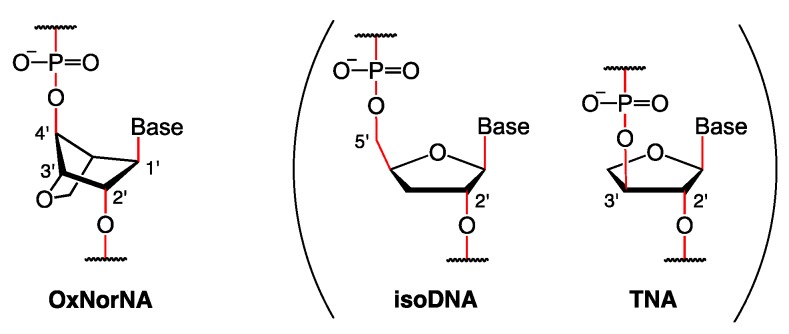
Structures of artificial nucleic acid bearing an oxanorbornane scaffold (OxNorNA), isoDNA, and TNA.

Throughout our research on conformational restrictions of nucleic acids, we newly designed an artificial nucleic acid bearing an oxanorbornane scaffold (OxNorNA) (Figure 2). Oxanorbornane, 2-oxabicyclo[2,2,1]heptane, has a rigid structure, and thus the relative position of the phosphodiester linkage and nucleobase parts of OxNorNA can be strictly fixed. The linkage part of OxNorNA is considered a 4′–2′ system based on the position of nucleobase. This type of linkage system is rarely seen in the other artificial nucleic acids, although the effects of 5′–2′ and 3′–2′ linkage systems (isoDNA and TNA, respectively) on the duplex-forming ability and antisense potency have been intensely investigated [14,15,16,17]. Herein, we report a robust synthetic route for OxNorNA phosphoramite with a uracil (U) nucleobase and the biophysical properties (duplex-forming ability, mismatch discrimination, and nuclease resistance) of OxNorNA-U-modified oligonucleotides.

## 2. Results and Discussion

### 2.1. Synthesis of OxNorNA-U Phosphoramidite

**Scheme 1:**
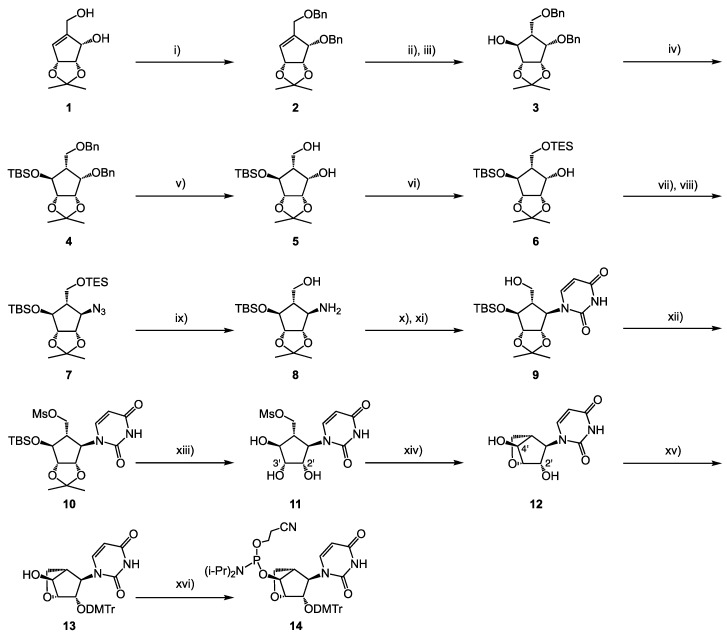
Synthesis of OxNorNA-uracil (U) phosphoramidite **14**. Reagents and conditions: (i) NaH, BnBr, DMF, 0 °C, 92%; (ii) thexylborane, THF, 0 °C to rt; (iii) NaBO_3_·H_2_O, H_2_O, 0 °C to rt, 90% over 2 steps; (iv) *tert*-butyldimethylchlorosilane (TBSCl), imidazole, DMF, rt, 74%; (v) HCOONH_4_, Pd/C, EtOH, reflux, 91%; (vi) triethylchlorosilane (TESCl), 2,6-lutidine, CH_2_Cl_2_, –78 °C, 86%; (vii) trifluoromethanesulfonic anhydride (Tf_2_O), pyridine, CH_2_Cl_2_, 0 °C; (viii) NaN_3_, DMF, rt, 71% over 2 steps; (ix) HCOONH_4_, Pd/C, THF, rt, 98%; (x) 3-methoxyacryloyl isocyanate, THF, –40 °C to rt; (xi) NH_4_OH, EtOH, 120 °C (sealed tube), 48% over 2 steps; (xii) methanesulfonyl chloride (MsCl), Et_3_N, CH_2_Cl_2_, 0 °C, 77%; (xiii) CeCl_3_·7H_2_O, oxalic acid, MeCN, rt, 66%; (xiv) aq. NaOH, 1,4-dioxane, rt, 72%; (xv) 4,4′-dimethoxytrityl trifluoromethanesulfonate (DMTrOTf), CH_2_Cl_2_, pyridine, 2,6-lutidine, 0 °C to rt, 93%; (xvi) 2-cyanoethyl *N*,*N*-diisopropylchlorophosphoramidite, *N*,*N*-diisopropylethylamine (DIPEA), 1-methylimidazole, MeCN, 0 °C to rt, 56%.

**Figure 3:**
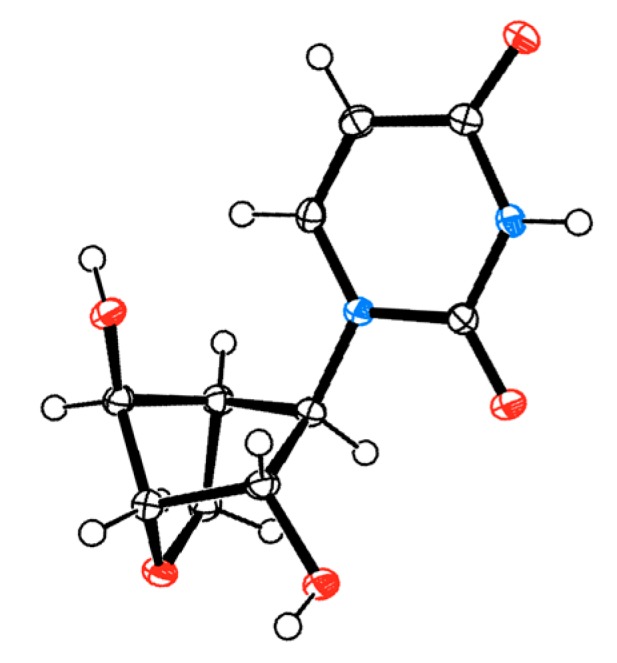
ORTEP drawing of the X-ray crystal structure of **12** (thermal ellipsoids at the 50% probability level).

Synthesis of OxNorNA-U phosphoramidite was started from cyclopentene **1**, derived over eight steps from commercially available d-ribose [18,19,20] (Scheme 1). At first, two hydroxyl groups of **1** were protected by a benzyl group, and the given compound **2** was subjected to a hydroboration reaction to produce the desired cyclopentanol **3** in a regio- and diastereoselective manner. Silylation was followed by hydrogenation using Pd/C afforded diol **5**. The primary alcohol of compound **5** was then selectively protected, and the remaining secondary alcohol was converted into a leaving group (OTf). Subsequent treatment of sodium azide afforded compound **7** at a 71% yield over two steps. The reduction of the azide group, using ammonium formate with Pd/C, underwent the removal of the TES group. A uracil ring was successively formed by a reaction with 3-methoxyacryloyl isocyanate, following the reported procedure [21,22,23]. The direct incorporation of the uracil nucleobase to the triflated compound of **6** was unsuccessful. The hydroxyl group of **9** was then mesylated, and the acetonide and TBS group were removed under mildly acidic conditions (CeCl_3_·7H_2_O, oxalic acid, MeCN, and rt) to afford triol **11**. Intramolecular cyclization of **11** by aqueous (aq.) NaOH regio-selectively afforded the desired oxanorbornane **12** at a 72% yield. The regioselectivity is explained by the difference in the uracil nucleobase steric repulsion of the two possible products. That is, **12** obtained from the S_N_2 reaction of the 3′-hydroxyl group of **11** has the uracil nucleobase at a less hindered position than the other product that can be produced from the S_N_2 reaction of the 2′-hydroxyl group. The structure of **12** was confirmed by NMR (For ^1^H, ^13^C, ^1^H–^1^H COSY, DEPT, HMQC, and HMBC NMR spectra, see Supplementary Material) and X-ray crystal structure (Figure 3). The less hindered 2′-hydroxyl group of **12** was then protected by the dimethoxytrityl (DMTr) group, and the remaining alcohol was phosphytilated to afford **14**, a suitable building block for the reverse (3′-to-5′) oligonucleotide synthesis. We note that tritylation of **12** by using DMTrCl in pyridine resulted in no reaction, but DMTrOTf generated from a reaction of DMTrCl and AgOTf in CH_2_Cl_2_ successfully afforded compound **13** in 93% [16,24].

### 2.2. Oligonucleotide Synthesis

Following the conventional phosphoramidite protocol, the given amidite **14** was successfully incorporated into the designed sequences (Table 1). The coupling time for the incorporation of the synthesized phosphoramidite **14** was prolonged to 12.5 min. In addition, the detritylation time of all reverse phosphoramidites was performed in 2 min. The above conditions afforded OxNorNA-U-modified oligonucleotides **ON1**–**ON3** in 22–47% yields (for high-performance liquid chromatography (HPLC) charts of purified oligonucleotides, see Supplementary Material).

### 2.3. Duplex-forming Ability and Thermodynamic Parameters

The thermal stabilities of the duplexes formed by the OxNorNA-U-modified oligonucleotides (**ON1** and **ON2**) with the complementary single-stranded DNA (ssDNA) or ssRNA were then evaluated by ultraviolet (UV) melting experiments. The given melting temperatures (*T*_m_ values) and thermodynamic parameters are summarized in Table 2 and Table 3 (for van’t Hoff plots, see Figures S1 and S2 in Supplementary Material). As for the ssDNA complement (sequence: 5′-d(AGCAAAAAACGC)-3′) (Table 2), **ON1** with a single OxNorNA modification was found to properly form a duplex. However, the **ON1**·ssDNA duplex furnished a *T*_m_ value of 45.4 °C, which was 4.5 °C lower *T*_m_ than that obtained for the corresponding **ON4**·ssDNA duplex (*T*_m_ = 49.9 °C). Further modification to **ON1** with OxNorNA resulted in a very low affinity for ssDNA (21.0 °C for **ON2**) as compared to the natural oligonucleotide (48.4 °C for **ON5**). Toward the ssRNA complement (sequence: 5′-r(AGCAAAAAACGC)-3′) (Table 3), OxNorNA-modified **ON1** and **ON2** denoted similar tends, but they were found to prefer ssRNA rather than ssDNA (see their Δ*T*_m_/mod. values).

The low *T*_m_ values of the OxNorNA-modified oligonucleotides might be explained by an unusual 4′–2′ linkage system that was changed from the general 5′–3′ linkage system found in the natural oligonucleotides. The linkage shift may lead the nucleobase of OxNorNA to an unfavorable direction for the duplex formations (for the structure comparison between OxNorNA-U and 2′,4′-BNA/LNA-U nucleosides, see reference [25]). Thermodynamic data obtained by van’t Hoff plots indicated that duplex formations by the OxNorNA-modified oligonucleotides were enthalpically unfavored (e.g., base stacking), and entropy factors (e.g., structural restriction) had no compensatory effects on the duplex formations.

### 2.4. Mismatch Discrimination

We also evaluated mismatch discrimination of the OxNorNA-U-modified oligonucleotide. As shown in Table 4 and Table 5, OxNorNA-U-modified **ON1** exhibited much lower *T*_m_ values toward the one-base-mismatched sequences (**N** = G, C, T, and U) than toward the fully matched sequence (**N** = A). The base discrimination patterns of OxNorNA-U-modified **ON1** were similar to the natural counterpart (**ON4**). This result indicated that a single modification with the adamant OxNorNA does not interfere with the common base-pairing rules, even the linkage moiety shifts from the natural nucleic acid.

### 2.5. Circular Dichroism Analysis

**Figure 4:**
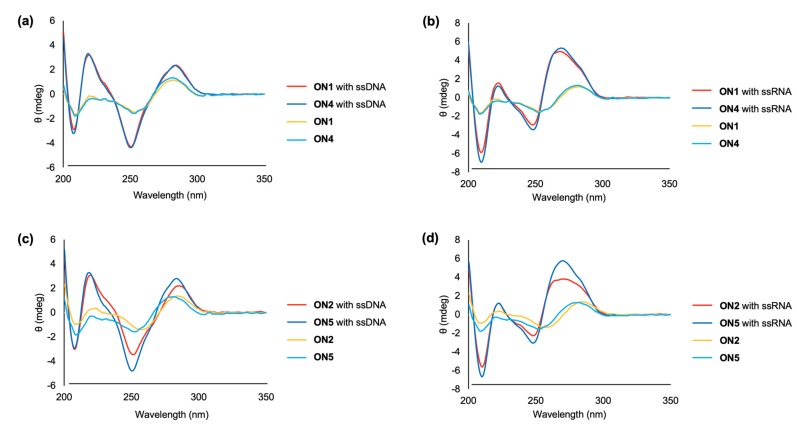
Circular dichroism (CD) spectra of OxNorNA-modified oligonucleotides (**ON1** and **ON2**) and their natural counterparts (**ON4** and **ON5**) in the presence or absence of a complementary strand (ssDNA or ssRNA). (**a**) **ON1** and **ON4** in the presence or absence of ssDNA; (**b**) **ON1** and **ON4** in the presence or absence of ssRNA; (**c**) **ON2** and **ON5** in the presence or absence of ssDNA; (**d**) **ON2** and **ON5** in the presence or absence of ssRNA. Conditions: 10 mM sodium phosphate buffer (pH 7.2) containing 100 mM NaCl and 4 μM of each oligonucleotide at 10 °C. The sequences of the target ssDNA and ssRNA are 5′-d(AGCAAAAAACGC)-3′ and 5′-r(AGCAAAAAACGC)-3′, respectively.

To analyze the structure of OxNorNA-U-modified oligonucleotide in a single-stranded or duplex form, circular dichroism (CD) spectra were recorded at 10 °C in the same buffer as that used for the UV melting experiments (Figure 4). As a result, **ON1** with one OxNorNA modification showed similar CD spectra to the natural **ON4** (Figure 4a,b); thus, **ON1** and **ON4** have similar secondary structures in the presence or absence of complementary strands (ssDNA and ssRNA). From the CD spectra in Figure 4b, **ON1**·ssRNA and **ON4**·ssRNA duplexes were found to form a typical A-form duplex (positive cotton band at 260 nm and negative cotton band at 210 nm). In contrast to the results in Figure 4a,b, **ON2**·ssDNA and **ON2**·ssRNA duplexes with multiple OxNorNA modifications displayed much weaker cotton effects than the corresponding **ON5**·ssDNA and **ON5**·ssRNA duplexes (Figure 4c,d). These results probably indicated disrupted base stacking in **ON2**·ssDNA and **ON2**·ssRNA duplexes and thus resulted in duplex destabilizations (low *T*_m_ values).

### 2.6. Stability Against Nuclease Digestion

**Figure 5:**
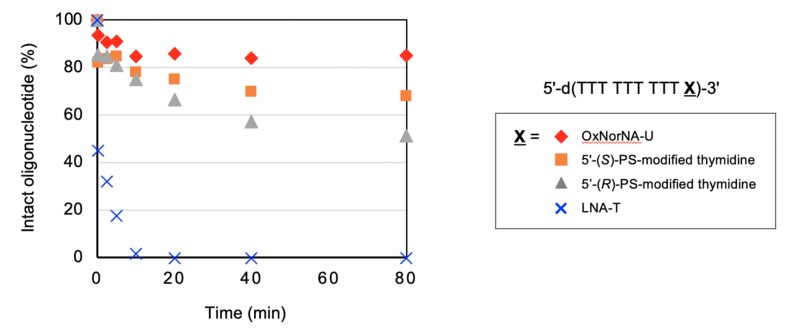
Enzymatic stability of the OxNorNA-modified oligonucleotide. Conditions: 0.133 ug/mL snake venom phosphodiesterase (svPDE), 10 mM MgCl_2_, 50 mM Tris-HCl (pH 8.0), and 2 μM each oligonucleotide at 37 °C. The sequence of the oligonucleotides used was 5′-d(TTT TTT TTT **X**)-3′. **X** = OxNorNA-U (red diamond, **ON3**), **X** = 5′-(*S*)-phosphorothioate (PS)-modified thymidine (orange square, **ON6**), **X** = 5′-(*R*)-PS-modified thymidine (gray triangle, **ON7**), and **X** = locked nucleic acid (LNA)-T (blue cross, **ON 8**).

We next investigated the effect of OxNorNA modification on the stability of oligonucleotides against enzymatic degradation. Oligonucleotides with 3′-terminal modifications (sequence: 5′-d(TTT TTT TTT **X**)-3′, **X** = OxNorNA-U (**ON3**); **X** = 5′-(*R*)-phosphorothioate (PS)-modified thymidine (**ON6**); **X** = 5′-(*S*)-PS-modified thymidine (**ON7**); **X** = locked nucleic acid (LNA) (**ON8**)) were prepared, incubated with 0.133 ug/mL 3′-exonuclease (snake venom phosphodiesterase, svPDE) at 37 °C, and the percentage of the remaining intact oligonucleotides was monitored by HPLC (Figure 5). Under the conditions we tested, the oligonucleotide modified with LNA (**ON8**) was digested within 20 min. Commonly used phosphorothioate (PS) modifications were found to be effective for improving the oligonucleotide stability against nuclease, but more than 30% of **ON6** (*S*p) and **ON7** (*R*p) were cleaved at 80 min. Conversely, over 80% of OxNorNA-modified **ON3** was still intact after 80 min. It was expected that OxNorNA had successfully escaped from the nuclease recognition since it had an altered scaffold from the natural nucleoside and an unusual 4′–2′ linkage system.

## 3. Materials and Methods

### 3.1. General Information

Reagents and solvents were purchased from commercial suppliers and used without purification unless otherwise specified. All experiments involving air and/or moisture-sensitive compounds were carried out under an Ar atmosphere. All reactions were monitored with analytical thin-layer chromatography (TLC; silica gel coated with fluorescent indicator F254; Merck, Darmstadt, Germany). Flash column chromatography was carried out using EPCLC–W–Prep 2XY (Yamazen, Osaka, Japan). NMR spectra were recorded on JNM-ECA-500, JNM-ECS-400, and JNM-ECS-300 spectrometers (JEOL, Akishima, Japan) using CDCl_3_ or DMSO–*d*_6_ as the solvent with tetramethylsilane (TMS) as an internal standard. For ^31^P NMR, 5% H_3_PO_4_ in D_2_O (0.00 ppm) was used as an external standard. Infrared (IR) spectra were recorded on an FT/IR–4200 spectrophotometer (JASCO, Hachioji, Japan). Optical rotations were recorded on a P–2200 instrument (JASCO). Mass spectra of all new compounds were measured on a SpiralTOF JMS-S3000 (for electrospray ionization, ESI) or a JMS-700 instrument (JEOL) (for fast atom bombardment, FAB). Solid-phase oligonucleotide synthesis was performed using an nS–8 Oligonucleotide Synthesizer (GeneDesign, Ibaraki, Japan). Matrix-assisted laser desorption/ionization-time of flight (MALDI-TOF) mass spectra were recorded on an ultrafleXtreme or an Autoflex II mass spectrometer (Bruker Daltonics, Billerica, MA, USA). For HPLC, Shimadzu DGU-20A_3R_, LC-20AD, CBM-20A, CTO-20AC, SPD-20A, and FRC-10A were utilized. For UV absorbance measurements, a UV-1800 spectrometer (Shimadzu, Kyoto, Japan) was utilized. UV melting experiments were performed using a UV–1650 or UV-1800 UV–Vis spectrophotometer equipped with a TMSPC–8 *T*_m_ analysis accessory (Shimadzu).

### 3.2. Synthesis of Compound ***2***

Compound **1** (3.0 g, 16.1 mmol) was dissolved in dry DMF (160 mL), and the solution was cooled in an ice bath. Sodium hydride (50% w/w in mineral oil, 2.30 g) was carefully added to the solution, and the resulting mixture was stirred for 30 min. Benzylbromide (5.7 mL, 48.3 mmol) was then added dropwise, and the mixture was further stirred for 2 h at 0 °C. After the reaction was finished, the mixture was partitioned between CH_2_Cl_2_ and H_2_O. The separated organic layer was washed with brine, dried over Na_2_SO_4_, and concentrated in vacuo. The residue was purified by silica gel column chromatography, eluted with hexane/AcOEt (7:1), to give compound **2** as a colorless oil (5.4 g, 92%). IR (KBr): ν_max_ 3031, 2985, 2933, 2858, 1605, 1496, 1454 cm^−1^; [α]_D_^23^ −98.3 (c 1.00, MeOH); ^1^H-NMR (500 MHz, CDCl_3_): *δ* 7.40–7.25 (10H, m), 5.88–5.86 (1H, m), 4.94 (1H, dd, *J* = 5.7, 1.2 Hz), 4.85 (1H, d, *J* = 12.0 Hz), 4.77 (1H, dd, *J* = 5.7, 5.2 Hz), 4.59 (1H, d, *J* = 12.0 Hz), 4.54 (1H, d, *J* = 11.5 Hz), 4.48 (1H, d, *J* = 11.5 Hz), 4.33–4.35 (1H, m), 4.15 (2H, s), 1.46 (3H, s), 1.40 (3H, s); ^13^C-NMR (125 MHz, CDCl_3_): *δ* 144.4, 138.4, 138.0, 128.3, 128.2, 127.9, 127.8, 127.6, 127.6, 127.5, 112.1, 82.3, 79.7, 77.6, 72.8, 71.9, 66.5, 27.7, 26.7; MS (FAB) *m/z* 389 [M + Na]^+^; HRMS (FAB) Calcd. for C_23_H_26_O_4_Na [M + Na]^+^: 389.1729; found: 389.1731.

### 3.3. Synthesis of Compound ***3***

Compound **2** (6.9 g, 18.9 mmol) was dissolved in dry THF (189 mL), and the solution was cooled in an ice bath. Thexylborane (0.5 M in THF, 120 mL) was added dropwise to the solution, and the resulting mixture was stirred for 3 h at room temperature. After TLC indicated the consumption of the starting material, the mixture was cooled in an ice bath and was added to NaBO_3_·4H_2_O (14.6 g, 95 mmol) and H_2_O (60 mL). The mixture was stirred for 1 h at room temperature, diluted with AcOEt, and washed with H_2_O. The organic layer was further washed with brine, dried over Na_2_SO_4_, and concentrated in vacuo. The residue was purified by silica gel column chromatography, eluted with hexane/AcOEt (3:1), to give compound **3** as a colorless oil (6.5 g, 90%). IR (KBr): ν_max_ 3449, 3063, 3031, 2984, 2928, 2917, 2867, 1497, 1454 cm^−1^; [α]_D_^25^ −65.5 (c 1.00, MeOH); ^1^H-NMR (500 MHz, CDCl_3_): δ 7.35–7.25 (10H, m), 4.78 (1H, d, *J* = 12.6 Hz), 4.64 (1H, dd, *J* = 7.5, 4.6 Hz), 4.46–4.53 (3H, m), 4.44 (1H, dd, *J* = 6.9, 2.3 Hz), 4.26–4.30 (1H, m), 3.98 (1H, dd, *J* = 5.2, 4.6 Hz), 3.73–3.76 (2H, m), 2.43 (1H, d, *J* = 2.3 Hz), 2.24–2.32 (1H, m), 1.43 (3H, s), 1.31 (3H, s); ^13^C-NMR (125 MHz, CDCl_3_): *δ* 138.4, 138.0, 128.4, 128.2, 127.8, 127.7, 127.6, 127.5, 113.0, 86.1, 80.0, 78.0, 77.7, 73.4, 72.8, 68.4, 50.1, 25.9, 24.5; MS (FAB) *m/z* 385 [M + H]^+^; HRMS (FAB) Calcd. for C_23_H_29_O_5_ [M + H]^+^: 385.2015; found: 385.2016.

### 3.4. Synthesis of Compound ***4***

Compound **3** (3.00 g, 7.81 mmol) was dissolved in dry DMF (80 mL), and imidazole (1.60 g, 23.4 mmol) and *tert*-butyldimethylchlorosilane (2.36 g, 15.6 mmol) were added. The resulting mixture was stirred for 1 h at room temperature. After the reaction was finished, the mixture was diluted with AcOEt and washed with saturated aq. NaHCO_3_. The organic layer was washed with brine, dried over Na_2_SO_4_, and concentrated in vacuo. The residue was purified by silica gel column chromatography, eluted with hexane/AcOEt (8:1), to give compound **4** as a colorless oil (2.9 g, 74%). IR (KBr): ν_max_ 3088, 3064, 3031, 2979, 2951, 2928, 2856, 1633, 1605, 1471, 1462, 1532 cm^−1^; [α]_D_^25^ −37.2 (c 1.00, MeOH); ^1^H-NMR (500 MHz, CDCl_3_): *δ* 7.23–7.36 (10H, m), 4.68–4.72 (1H, m), 4.51–4.64 (2H, m), 4.52 (1H, d, *J* = 12.1 Hz), 4.44 (1H, d, *J* = 12.1 Hz), 4.29–4.31 (1H, m), 4.11–4.13 (1H, m), 4.05–4.09 (1H, m), 3.77 (1H, dd, *J* = 9.2, 6.3 Hz), 3.62 (1H, dd, *J* = 9.2, 8.6 Hz), 2.30–2.38 (1H, m), 1.41 (3H, s), 1.28 (3H, s), 0.85 (9H, s), 0.08 (3H, s), 0.06 (3H, s); ^13^C-NMR (125 MHz, CDCl_3_): *δ* 138.7, 138.4, 128.3, 127.9, 127.6, 127.6, 127.4, 112.0, 86.4, 79.5, 77.8, 76.9, 76.6, 73.0, 72.6, 67.2, 50.6, 25.8, 25.7, 25.6, 24.0, 17.9, −4.8, −5.0; MS (FAB) *m/z* 499 [M + H]^+^; HRMS (FAB) Calcd. for C_29_H_43_O_5_Si [M + H]^+^: 499.2880; found: 499.2884.

### 3.5. Synthesis of Compound ***5***

Compound **4** (2.87 g, 5.75 mmol) was dissolved in EtOH (60 mL), and ammonium formate (1.60 g, 25.4 mmol) and 20% Pd(OH)_2_/C (500 mg) were added. The resulting suspension was refluxed for 3 h. The reaction mixture was cooled to room temperature and filtrated through a Celite pad. The filtrate was concentrated in vacuo, and the resulting residue was purified by silica gel column chromatography, eluted with hexane/AcOEt (1:4), to give compound **5** as a colorless oil (1.7 g, 91%). IR (KBr): ν_max_ 3427, 2954, 2932, 2858, 1633, 1605, 1472, 1463 cm^−1^; [α]_D_^26^ −44.0 (c 1.00, MeOH); ^1^H-NMR (500 MHz, CDCl_3_): *δ* 4.56 (1H, dd, *J* = 7.5, 5.2 Hz), 4.41 (1H, dd, *J* = 7.5, 3.4 Hz), 4.28 (1H, dd, *J* = 9.2, 3.5 Hz), 4.18–4.23 (1H, m), 3.81–3.93 (2H, m), 2.71–2.75 (1H, m), 1.97–2.03 (1H, m), 1.51 (3H, s), 1.34 (3H, s), 0.89 (9H, s), 0.11 (3H, s), 0.10 (3H, s); ^13^C-NMR (125 MHz, CDCl_3_): *δ* 113.6, 87.1, 78.3, 71.4, 60.3, 53.0, 26.0, 25.7, 24.6, 17.9, −4.7, −5.2; MS (FAB) *m/z* 319 [M + H]^+^; HRMS (FAB) Calcd. for C_15_H_31_O_5_Si [M + H]^+^: 319.1941; found: 319.1950.

### 3.6. Synthesis of Compound ***6***

Compound **5** (2.90 g, 9.09 mmol) was dissolved in dry CH_2_Cl_2_ (90 mL), and 2,6-lutidine (3.2 mL, 27.3 mmol) was added. The mixture was cooled to −78 °C, and then chlorotriethylsilane (1.68 mL, 10.0 mmol) was added dropwise. The resulting mixture was stirred for 1 h at −78 °C. The reaction was quenched with an addition of MeOH and diluted with CH_2_Cl_2_. The mixture was partitioned between CH_2_Cl_2_ and saturated aq. NaHCO_3_. The organic layer was concentrated in vacuo, and the resulting residue was purified by silica gel column chromatography, eluted with hexane/AcOEt (8:1), to give compound **6** as a colorless oil (3.39 g, 86%). IR (KBr): ν_max_ 3530, 2954, 2877, 2856 cm^−1^; [α]_D_^22^ −32.6 (c 1.00, MeOH); ^1^H-NMR (500 MHz, CDCl_3_): *δ* 4.57 (1H, dd, *J* = 7.5, 5.2 Hz), 4.36 (1H, dd, *J* = 6.9, 2.3 Hz), 4.22 (1H, dd, *J* = 8.6, 4.6 Hz), 3.92–4.00 (2H, m), 3.69 (1H, dd, *J* = 9.8, 5.7 Hz), 2.66–2.68 (1H, m), 2.08–2.16 (1H, m), 1.50 (3H, s), 1.32 (3H, s), 0.96 (9H, t, *J* = 8.0 Hz), 0.88 (9H, s), 0.60 (6H, q, *J* = 8.0 Hz), 0.08 (3H, s), 0.05 (3H, s); ^13^C-NMR (125 MHz, CDCl_3_): *δ* 112.9, 86.9, 79.0, 69.8, 59.7, 54.2, 25.9, 25.7, 24.3, 17.9, 6.7, 4.3, −4.7, −5.1; MS (FAB) *m/z* 433 [M + H]^+^; HRMS (FAB) Calcd. for C_21_H_44_O_5_Si_2_ [M + H]^+^: 433.2806; found: 433.2804.

### 3.7. Synthesis of Compound ***7***

Compound **6** (3.20 g, 7.37 mmol) was dissolved in dry pyridine (74 mL), and the solution was cooled in an ice bath. Trifluoromethanesulfonic anhydride (1.5 mL) in dry CH_2_Cl_2_ (20 mL) was added dropwise, and the reaction mixture was stirred for 1 h at 0 °C. After the addition of water, the resulting mixture was extracted with CH_2_Cl_2_. The organic layer was washed with brine, dried over Na_2_SO_4_, and concentrated in vacuo. The crude product was co-evaporated with toluene three times and then used immediately for the next reaction without further purification. The triflate was dissolved in dry DMF (74 mL), and sodium azide (1.44 g, 22.1 mmol) was added. The resulting mixture was stirred for 8 h at room temperature. After the addition of water, the resulting mixture was extracted with AcOEt. The organic layer was washed with brine, dried over Na_2_SO_4_, and concentrated in vacuo. The resulting residue was purified by silica gel column chromatography, eluted with hexane/AcOEt (12:1), to give compound **7** as a colorless oil (2.40 g, 71% in 2 steps). IR (KBr): ν_max_ 2879, 2103 cm^−1^; [α]_D_^24^ −27.1 (c 1.00, MeOH); ^1^H-NMR (500 MHz, CDCl_3_): *δ* 4.38 (1H, dd, *J* = 8.0, 5.2 Hz), 4.27 (1H, dd, *J* = 7.5, 3.4 Hz), 3.95 (1H, dd, *J* = 9.2, 4.0 Hz), 3.63 (1H, dd, *J* = 5.2, 10.3 Hz), 3.61–3.59 (2H, m), 1.87–1.81 (1H, m), 1.38 (3H, s), 1.21 (3H, s), 0.89 (9H, t, *J* = 8.0 Hz), 0.81 (9H, s), 0.53 (6H, q, *J* = 8.0 Hz), 0.03 (3H, s), 0.00 (3H, s); ^13^C-NMR (125 MHz, CDCl_3_): *δ* 112.9, 85.9, 83.1, 75.3, 64.6, 57.7, 53.8, 27.1, 25.7, 25.2, 17.9, 6.75, 4.33, −4.59, −5.19; MS (FAB) *m/z* 480 [M + Na]^+^; HRMS (FAB) Calcd. for C_21_H_43_N_3_O_4_Si_2_Na [M + Na]^+^: 480.2690; found: 480.2701.

### 3.8. Synthesis of Compound ***8***

Compound **7** (10.1 g, 22.1 mmol) was dissolved in EtOH (220 mL), and ammonium formate (6.97 g, 111 mmol) and 20% Pd(OH)_2_/C (2.00 g) were added. The suspension was vigorously stirred for 3 h at room temperature and then filtrated through a Celite pad. The filtrate was concentrated in vacuo, and the resulting residue was purified by silica gel column chromatography, eluted with CHCl_3_/MeOH (8:2), to give compound **8** as a white solid (6.9 g, 98%). IR (KBr): ν_max_ 3184, 2926, 2858, 1596 cm^−1^; [α]_D_^23^ −13.4 (c 1.00, MeOH); ^1^H-NMR (500 MHz, DMSO-*d_6_*): *δ* 8.33 (1H, s), 5.80 (2H, brs), 4.41 (1H, dd, *J* = 4.6, 7.5 Hz), 4.30 (1H, dd, *J* = 4.6, 7.5 Hz), 3.88 (1H, dd, *J* = 5.2, 9.7 Hz), 3.60 (1H, dd, *J* = 3.5, 11.5 Hz), 3.38 (1H, dd, *J* = 4.0, 11.5 Hz), 3.09 (1H, dd, *J* = 4.6, 10.3 Hz), 1.86–1.78 (1H, m), 1.38 (3H, s), 1.21 (3H, s); ^13^C-NMR (125 MHz, DMSO-*d_6_*): *δ* 165.7, 112.0, 85.3, 82.7, 75.8, 56.1, 54.3, 53.4, 27.0, 25.7, 25.0, 17.7, −4.6, −5.0; MS (FAB) *m/z* 318 [M + H]^+^; HRMS (FAB) Calcd. for C_15_H_32_NO_4_Si [M + H]^+^: 318.2101; found: 318.2104.

### 3.9. Synthesis of Compound ***9***

An amount of 3-methoxyacryloyl chloride (8.52 g, 70.8 mmol) was added to a suspension of silver cyanate (10.6 g, 70.8 mmol) in dry benzene (300 mL), and the mixture was refluxed for 30 min and cooled to room temperature. The resulting supernatant was slowly added over 15 min to a solution of compound **8** (7.5 g, 23.6 mmol) in dry THF (300 mL) at −40 °C. The mixture was allowed to gradually warm to room temperature and was stirred for 16 h. After the solvents were removed in vacuo, the residue was purified by silica gel column chromatography, eluted with hexane/AcOEt (1:2), to give a white solid. The given solid was dissolved in EtOH (60 mL) and treated with ammonium hydride (60 mL, 28% in water) in a sealed tube at 120 °C for 4 h. The resulting mixture was cooled at room temperature. After concentrated in vacuo, the residue was purified by silica gel column chromatography, eluted with hexane/AcOEt (1:2), to give compound **9** as a white solid (4.6 g, 48% in 2 steps). IR (KBr): ν_max_ 3156, 2931, 2890, 2858, 1715, 1472 cm^−1^; [α]_D_^23^ −16.2 (c 1.00, MeOH); ^1^H-NMR (500 MHz, CDCl_3_): *δ* 9.60 (1H, brs), 7.32 (1H, d, *J* = 8.0 Hz), 5.72 (1H, d, *J* = 8.0 Hz), 4.82 (1H, dd, *J* = 7.4, 5.2 Hz), 4.53 (1H, dd, *J* = 10.3, 4.6 Hz), 4.47 (1H, dd, *J* = 7.5, 4.1 Hz), 4.05 (1H, dd, *J* = 8.6, 4.0 Hz), 3.57–3.72 (2H, m), 2.64 (1H, brs), 2.34–2.44 (1H, m), 1.51 (3H, s), 1.28 (3H, s), 0.89 (9H, s), 0.14 (3H, s), 0.10 (3H, s); ^13^C-NMR (125 MHz, CDCl_3_): *δ* 163.4, 151.3, 142.9, 113.2, 113.2, 102.8, 85.6, 80.7, 62.9, 58.8, 52.6, 27.1, 25.7, 25.6, 25.0, 17.9, −4.6, −5.2; MS (FAB) *m/z* 413 [M + H]^+^; HRMS (FAB) Calcd. for C_19_H_33_N_2_O_6_Si [M + H]^+^: 413.2108; found: 413.2102.

### 3.10. Synthesis of Compound ***10***

Compound **9** (4.60 g, 11.2 mmol) was dissolved in dry CH_2_Cl_2_ (110 mL), and Et_3_N (4.68 mL, 33.6 mmol) was added. The mixture was cooled to 0 °C, and methanesulfonyl chloride (952 µL, 12.3 mmol) was added dropwise. The resulting mixture was stirred for 1 h at 0 °C. The reaction was quenched with an addition of MeOH and diluted with CH_2_Cl_2_. The mixture was then partitioned between CH_2_Cl_2_ and saturated aq. NaHCO_3_. The organic layer was concentrated in vacuo and the resulting residue was purified by silica gel column chromatography, eluted with hexane/AcOEt (1:2), to give compound **10** as a white solid (4.2 g, 77%). IR (KBr): ν_max_ 3173, 2989, 2931, 2886, 2858, 1714, 1472 cm^−1^; [α]_D_^23^ −24.1 (c 1.00, MeOH); ^1^H-NMR (500 MHz, DMSO-*d_6_*): *δ* 11.34 (1H, brs), 7.81 (1H, d, *J* = 8.0 Hz), 5.61 (1H, dd, *J* = 8.0, 2.3 Hz), 4.75 (1H, dd, *J* = 7.5, 5.2 Hz), 4.49–4.57 (1H, m), 4.47 (1H, dd, *J* = 8.1, 5.2 Hz), 4.12–4.22 (2H, m), 3.88 (1H, dd, *J* = 10.4, 5.8 Hz), 3.12 (3H, s), 2.67–2.79 (1H, m), 1.43 (3H, s), 1.22 (3H, s), 0.87 (9H, s), 0.11 (3H, s), 0.08 (3H, s); ^13^C-NMR (125 MHz, DMSO-*d_6_*): *δ* 163.2, 151.1, 112.7, 102.1, 84.1,79.6, 75.6, 66.3, 48.1, 36.2, 27.1, 25.6, 25.1, 17.6, 17.6, −4.6, −5.2; MS (FAB) *m/z* 491 [M + H]^+^; HRMS (FAB) Calcd. for C_20_H_34_N_2_O_8_Si [M + Na]^+^: 491.1883; found: 491.1881.

### 3.11. Synthesis of Compound ***11***

Compound **10** (4.20 g, 8.57 mmol) was dissolved in MeCN (86 mL), and oxalic acid (77 mg, 0.86 mmol) and CeCl_3_·7H_2_O (9.58 g, 25.7 mmol) were added. The resulting mixture was stirred for 3 d at room temperature and then filtrated through a Celite pad. The filtrate was concentrated in vacuo, and the resulting residue was purified by silica gel column chromatography, eluted with CHCl_3_/MeOH (7:3), to give compound **11** as a white solid (1.9 g, 66%). IR (KBr): ν_max_ 3343, 2931, 1690, 1349, 1173 cm^−1^; [α]_D_^25^ −75.5 (c 1.00, MeOH); ^1^H-NMR (500 MHz, DMSO-*d_6_*): *δ* 11.2 (1H, s), 7.60 (1H, d, *J* = 8.1 Hz), 5.62 (1H, dd, *J* = 8.1, 2.3 Hz), 5.34 (1H, brs), 4.93 (1H, brs), 4.47–4.42 (1H, m), 4.26–4.13 (3H, m), 3.72 (1H, dd, *J* = 5.2, 2.9 Hz), 3.58 (1H, dd, *J* = 5.2, 3.5 Hz), 3.12 (3H, s), 2.19–2.11 (1H, m); ^13^C-NMR (125 MHz, DMSO-*d_6_*): *δ* 163.3, 151.4, 143.0, 101.8, 75.5, 74.5, 72.6, 69.8, 62.2, 46.6, 36.4; MS (FAB) *m/z* 337 [M + H]^+^; HRMS (FAB) Calcd. for C_11_H_17_N_2_O_8_BS [M + H]^+^: 337.0706; found: 337.0700.

### 3.12. Synthesis of Compound ***12***

Compound **11** (1.90 g, 5.65mmol) was dissolved in 1,4-dioxane (57 mL), and 2N aq. NaOH (12 mL) was added. The resulting mixture was stirred for 2 h at room temperature. The mixture was concentrated in vacuo, and the resulting residue was purified by silica gel column chromatography, eluted with CHCl_3_/MeOH (8:2), and by crystallization with AcOEt/MeOH (1:1) to give compound **12** as colorless crystals (980 mg, 72%). IR (KBr): ν_max_ 3387, 1685 cm^−1^; [α]_D_^22^ −170.2 (c 1.00, MeOH); ^1^H-NMR (500 MHz, DMSO-*d_6_*): *δ* 11.28 (1H, brs), 8.05 (1H, d, *J* = 13.8 Hz), 5.87 (1H, s), 5.54 (1H, d, *J* = 13.0 Hz), 5.02 (1H, d, *J* = 10.7 Hz), 4.54–4.43 (1H, m), 4.32–4.19 (2H, m), 3.87 (1H, dd, *J* = 3.8, 12.2 Hz), 3.76 (1H, s), 3.64–3.53 (1H, m), 2.33 (1H, s); ^13^C-NMR (125 MHz, DMSO-*d_6_*): *δ* 163.2, 151.9, 143.7, 100.0, 79.1, 78.2, 75.6, 71.4, 65.5, 45.8; MS (FAB) *m/z* 241 [M + H]^+^; HRMS (FAB) Calcd. for C_10_H_13_N_2_O_5_ [M + H]^+^: 241.0824; found: 241.0826.

### 3.13. Synthesis of Compound ***13***

Silver triflate (241 mg, 0.938 mmol) was added portion wise to a solution of 4,4′-dimethoxytrityl chloride (337 mg, 0.995 mmol) in dry CH_2_Cl_2_ (1.5 mL). After stirring for 2 h at room temperature, the mixture left to stand for 1 h to precipitate AgCl. The supernatant (375 μL) containing DMTrOTf was added to a solution of compound **12** (30.0 mg, 0.125 mmol) in dry pyridine (370 μL) and dry 2,6-lutidine (370 μL) at 0 °C, and the resulting mixture was stirred for 4 h at room temperature. The reaction was quenched with additions of saturated aq. NaHCO_3_ and saturated aq. CuSO_4_. The mixture was then filtrated through a Celite pad, and the filtrate was partitioned between AcOEt and saturated aq. NaHCO_3_. The organic layer was then concentrated in vacuo, and the resulting residue was purified by silica gel column chromatography, eluted with CHCl_3_/MeOH (9:1), to give compound **13** as a white solid (63 mg, 93%). IR (KBr): ν_max_ 3405, 3063, 2941, 2843, 1687, 1507, 1613, 1466 cm^−1^; [α]_D_^28^ −1.97 (c 1.00, MeOH); ^1^H-NMR (300 MHz, DMSO-*d_6_*): *δ* 11.3 (1H, d, *J* = 1.8 Hz), 7.82 (1H, d, *J* = 8.3 Hz), 5.44–5.48 (2H, dd, *J* = 9.6, 4.8 Hz), 7.26–7.34 (7H, m), 6.85–6.92 (4H,m), 5.96 (1H, d, *J* = 3.2 Hz), 5.46 (1H, dd, *J* = 3.2, 1.6 Hz), 4.86 (1H, d, *J* = 4.1Hz), 4.50 (1H,d, *J* = 4.6 Hz), 4.11 (1H, s), 3.89 (1H, dd, *J* = 4.6, 2.3 Hz), 3.78 (6H, d, *J* = 4.1 Hz), 3.73–3.78 (1H, m), 2.69 (1H, s), 2.22 (1H, s); ^13^C-NMR (75 MHz, DMSO-*d_6_*): *δ* 163.5, 168.6, 152.2, 145.9, 143.8, 136.4, 130.2, 128.3, 128.0, 127.2, 113.7, 101.1, 86.5, 81.8, 78.1, 75.8, 72.5, 63.8, 55.5, 46.1, 31.4; MS (FAB) *m/z* 543 [M + H]^+^; HRMS (FAB) Calcd. for C_31_H_31_N_2_O_7_ [M + H]^+^: 543.2131; found: 543.2129.

### 3.14. Synthesis of Compound ***14***

*N*,*N*-Diisopropylethylamine (40 μL, 0.231 mmol), 1-methylimidazole (9.2 μL, 0.116 mmol), and 2-cyanoethyl *N*,*N*-diisopropylchlorophosphoramidite (26 μL, 0.116 mmol) were added to a degassed solution of compound **13** (21 mg, 0.0387 mmol) in dry MeCN (385 μL) at 0 °C. The resulting mixture was stirred for 14 h at room temperature. The reaction was quenched with an addition of saturated aq. NaHCO_3_. The mixture was concentrated in vacuo, and the resulting residue was purified by silica gel column chromatography, eluted with hexane/AcOEt (7:1), and reprecipitation to give compound **14** as a white solid (16 mg, 56%). IR (KBr): ν_max_ 3469, 3064, 2964, 2925, 1690, 1507, 1615, 1460 cm^−1^; ^1^H-NMR (400 MHz, CDCl_3_): *δ* 8.53 (1H, s), 7.28–7.50 (8H, m), 7.17–7.26 (2H, m), 6.76–6.82 (4H, m), 5.46 (1/2H, d, *J* = 8.2 Hz), 5.40 (1/2H, d, *J* = 8.2 Hz), 4.88 (1/2H, d, *J* = 3.7 Hz), 4.80 (1/2H, d, *J* = 3.7 Hz), 4.59 (1/2H, d, *J* = 4.1 Hz), 4.54 (1/2H, d, *J* = 4.1 Hz), 4.29 (1H, d, *J* = 8.7 Hz), 3.99–4.00 (1H, m), 3.77 (3H, d, *J* = 3.2 Hz), 3.76 (3H, d, *J* = 2.3 Hz), 3.60–3.68 (2H, m), 3.42–3.50 (2H, m), 3.32 (1/2H, s), 3.08 (1/2H, s), 2.40–2.55 (1H, m), 2.29–2.30 (1H, m), 1.28–1.29 (2H, m), 1.16 (3H, s), 1.14 (3H, s), 1.04 (3H, d, *J* = 3.7 Hz), 1.03 (3H, d, *J* = 3.7 Hz); ^13^C-NMR (100 MHz, CDCl_3_): δ 158.6, 145.1, 143.2, 136.1, 136.0, 130.1, 130.0, 128.0, 127.9, 126.9, 117.1, 113.3 100.7, 87.0, 79.3, 72.0, 71.8, 64.9, 58.4, 58.2, 55.2, 46.0, 45.9, 43.3, 43.1, 43.0, 31.9, 31.6, 29.7, 29.6, 24.6, 24.5, 22.6, 20.0, 19.9, 14.1; ^31^P-NMR (162 MHz, CDCl_3_): *δ* 150.3, 149.2; MS (FAB) *m/z* 743 [M + H]^+^; HRMS (FAB) Calcd. for C_40_H_48_N_4_O_8_P [M + H]^+^: 743.3210; found: 743.3217.

### 3.15. Energy Minimized Structure (Spartan 16)

The molecular structure run out multi-step stabilized. At first, stable conformers were calculated by using conformer distribution (MMFF). The given conformers were further calculated using HF/3-21G (equilibrium geometry) and then ωB97X-D/6-31G* to eliminate high-energy and duplicate conformers. Finally, the most stable structures were searched by ωB97X-V/6-3111+G(2df, 2p) density functional model.

### 3.16. X-ray Crystal Structure

A suitable crystal of compound **12** was carefully selected under an optical microscope and glued to thin glass fibers and mounted on the goniometer in a liquid nitrogen flow. X-ray diffraction data were collected on a Rigaku R-AXIS RAPID diffractometer employing graphite-monochromated CuKα radiation. The structure was solved by the direct method with the SIR-88 program [26] and refined with the SHELXL program [27]. The structural model was drawn with the ORTEP-3 program [28]. Further information on the crystal structure determinations has been deposited with the Cambridge Crystallographic Data Center (1989958). The data can be obtained free of charge via http://www.ccdc.cam.ac.uk/conts/retrieving.html (or from the CCDC, 12 Union Road, Cambridge CB2 1EZ, UK; Fax: +44 1223 336033; E-mail: deposit@ccdc.cam.ac.uk).

### 3.17. Oligonucleotide Synthesis

Oligonucleotides modified with OxNorNA-U (**ON1**–**ON3**) were synthesized by the reverse DNA synthesis using an nS–8 Oligonucleotide Synthesizer (GeneDesign) and a universal CPG solid support (0.2 µmol scale, Glen Research). The coupling time for OxNorNA phosphoramidite was extended to 12.5 min, and 5-[3,5-bis(trifluoromethyl)phenyl]-1*H*-tetrazole was used as an activator. The detritylation time of all reverse phosphoramidites was prolonged to 2 min (from 20 s), and 3 w/v% trichloroacetic acid/dichloromethane solution was used for the detritylation. The other synthetic procedures involved the standard phosphoramidite protocols. Cleavage from the solid support and removal of protecting groups was accomplished by mild conditions (*tert*-butylamine/water (1:3 v/v), 24 h at room temperature, and then 12 h at 60 °C). The resulting oligonucleotides were briefly purified using reverse-phase HPLC (Waters XTerra^®^ MS C_18_ 2.5 µm, 10 × 50 mm column, eluent A (0.1 M triethylammonium acetate (TEAA) in water), eluent B (MeCN), gradient: 5–9% or 6–15% of eluent B, flow rate = 5.0 mL/min). The resulting oligonucleotides were further purified with Sep-Pak^®^ Plus C18 cartridges, where the DMTr group was removed by 2% TFA in water, and by reverse-phase HPLC (Waters XTerra^®^ MS C_18_ 2.5 µm, 10 × 50 mm column, eluent A (0.1 M triethylammonium acetate (TEAA) in water), eluent B (MeCN), gradient: 5–9% or 6–15% of eluent B, flow rate = 5.0 mL/min). The purified oligonucleotides were analyzed using reverse-phase HPLC (Waters XTerra^®^ MS C_18_ 2.5 µm, 4.6 × 50 mm column), and their compositions were confirmed by MALDI-TOF MS analysis (matrix: 3-hydroxypicorinic acid, additive: diammonium hydrogen citrate).

### 3.18. UV Melting Experiment

The UV melting experiments were performed on Shimadzu UV-1650B and UV-1800 spectrometers equipped with a *T*_m_ analysis accessory. Samples containing oligonucleotide (4 µM), the target DNA or RNA (4 µM), and 100 mM NaCl in a 10 mM phosphate buffer (pH 7.2) were annealed at 100 °C and then cooled slowly to room temperature. The melting profile was recorded from 5 to 90 °C at a scan rate of 0.5 °C/min with detection at 260 nm. The *T*_m_ value was obtained from the temperature for half-dissociation of the formed duplexes based on the first derivative of the melting curve. For van’t Hoff plots, *T*_m_ values were determined at several oligonucleotide concentrations (0.90, 1.48, 2.44, 4.00, 6.52, and 13.6 μM) (see Figures S1 and S2 in Supplementary Material). The values of Δ*H*°, Δ*S*°, and Δ*G*° were calculated according to the equations shown below (R is an ideal gas constant, and C_t_ indicates oligonucleotide concentration):

1/*T*_m_ = (R/Δ*H*°)·ln(C_t_/4) + Δ*S*°/Δ*H*° (1)

Δ*G*° = Δ*H*° − TΔ*S*° (2)

### 3.19. CD Spectral Analysis

CD spectra were measured at 10 °C in a quartz cuvette with a 1-cm optical path length by using a J-720W spectrophotometer (JASCO). The sample solutions (360 μL) were prepared by dissolving oligonucleotide (4 μM) and NaCl (100 mM) in the phosphate buffer (10 mM, pH 7.2). The molar ellipticity was calculated from the equation [*θ*] = *θ*/*cl*, where *θ* is the relative intensity, *c* is the sample concentration, and *l* is the cell path length in centimeters.

### 3.20. Enzymatic Stability Analysis

Samples (each sample volume: 130 μL) containing MgCl_2_ (10 mM), oligonucleotide (2 μM), and snake venom phosphodiesterase (svPDE, 0.133 μg/mL) in the Tris-HCl buffer (50 mM, pH 8.0) were incubated at 37 °C. Portions of the samples (20 μL) were taken at respective time points, and svPDE was immediately deactivated by heating the sample at 90 °C for 2.5 min. The percentage of the remaining intact oligonucleotides was determined by reverse-phase HPLC (2.5 μm, 4.6 × 50 mm) and plotted against their reaction time.

## 4. Conclusions

We synthesized and evaluated structurally restricted OxNorNA-U-modified oligonucleotides. Although OxNorNA-U-modified oligonucleotides showed a lower duplex-forming ability as compared to the natural counterparts, the base discrimination ability of those was similar to that obtained for the natural oligonucleotides. Since OxNorNA has a rigid structure and exhibits extremely high enzymatic stability, we expect that OxNorNA could be useful for the point modifications of aptamers.

## Figures and Tables

**Table 1:** Isolated yields of OxNorNA-U-modified oligonucleotides, together with matrix-assisted laser desorption/ionization-time of flight mass spectra (MALDI-TOF MS) data.

ID	Sequence *^a^*	Yield (%)	[M − H]^−^	
			Calcd	Found
**ON1**	5′-d(GCG TTX TTT GCT)-3′	22	3630.4	3630.3
**ON2**	5′-d(GCG XTX TXT GCT)-3′	31	3626.3	3626.3
**ON3**	5′-d(TTT TTT TTT X)-3′	47	2977.0	2976.6

*^a^***X** = OxNorNA-U.

**Table 2:** *T*_m_ values and thermodynamic parameters of duplexes formed between oligonucleotides and the complementary single-stranded DNA (ssDNA) *^a^.*

ID	Sequence *^b^*	*T*_m_ (Δ*T*_m_/mod.) [°C]	Δ*H*° [kcal/mol]	Δ*S*° [kcal/mol]	Δ*G*° [kcal/mol]
**ON4**	5′-d(GCG TTU TTT GCT)-3′	49.9	−108.6	−310.1	−12.4
**ON1**	5′-d(GCG TTX TTT GCT)-3′	45.4 (−4.5)	−76.4	−214.8	−9.8
**ON5**	5′-d(GCG UTU TUT GCT)-3′	48.4	−81.4	−227.2	−11.0
**ON2**	5′-d(GCG XTX TXT GCT)-3′	21.0 (−9.1)	−42.0	−117.2	−5.6

*^a^* Conditions: 10 mM sodium phosphate buffer (pH 7.2) containing 100 mM NaCl and 4 μM of each oligonucleotide. *T*_m_ values are averages of at least three measurements. The sequence of ssDNA is 5′-d(AGCAAAAAACGC)-3′. Δ*T*_m_/mod.: the change in *T*_m_ value (Δ*T*_m_) per modification compared to the unmodified oligonucleotide. Δ*G*° values at 37 °C are shown. For thermodynamic parameters and van’t Hoff plots, see Table S1 and Figure S1 in Supplementary Material. *^b^* **X** = OxNorNA-U.

**Table 3:** *T*_m_ values and thermodynamic parameters of duplexes formed between oligonucleotides and the complementary single-stranded RNA (ssRNA) *^a^.*

ID	Sequence *^b^*	*T*_m_ (Δ*T*_m_/mod.) [°C]	Δ*H*° [kcal/mol]	Δ*S*° [kcal/mol]	Δ*G*° [kcal/mol]
**ON4**	5′-d(GCG TTU TTT GCT)-3′	46.5	−94.2	−268.3	−11.0
**ON1**	5′-d(GCG TTX TTT GCT)-3′	42.4 (−4.1)	−92.0	−265.9	−9.5
**ON5**	5′-d(GCG UTU TUT GCT)-3′	44.7	−86.8	−246.9	−10.2
**ON2**	5′-d(GCG XTX TXT GCT)-3′	24.7 (−6.7)	−56.3	−163.6	−5.5

*^a^* Conditions: 10 mM sodium phosphate buffer (pH 7.2) containing 100 mM NaCl and 4 μM of each oligonucleotide. *T*_m_ values are averages of at least three measurements. The sequence of ssRNA is 5′-r(AGCAAAAAACGC)-3′. Δ*T*_m_/mod.: the change in *T*_m_ value (Δ*T*_m_) per modification compared to the unmodified oligonucleotide. Δ*G*° values at 37 °C are shown. For thermodynamic parameters and van’t Hoff plots, see Table S2 and Figure S2 in Supplementary Material. *^b^* **X** = OxNorNA-U.

**Table 4:** Mismatch discrimination of natural and OxNorNA-modified oligonucleotides toward ssDNA *^a^*.

ID	Sequence *^b^*	*T*_m_ (Δ*T*_m_ = *T*_m_ [mismatch] − *T*_m_ [match]) [°C]			
		N = A	N = G	N = C	N = T
**ON4**	5′-d(GCG TTU TTT GCT)-3′	49.9	35.5 (−14.4)	38.5 (−11.4)	36.5 (−13.4)
**ON1**	5′-d(GCG TTX TTT GCT)-3′	45.4	33.2 (−12.2)	36.0 (−9.4)	32.5 (−12.9)

*^a^* Conditions: 10 mM sodium phosphate buffer (pH 7.2) containing 100 mM NaCl and 4 μM of each oligonucleotide. *T*_m_ values are averages of at least three measurements. The sequence of ssDNA is 5′-d(AGCAAA**N**AACGC)-3′. *^b^* **X** = OxNorNA-U.

**Table 5:** Mismatch discrimination of natural and OxNorNA-modified oligonucleotides toward ssRNA *^a.^*

ID	Sequence *^b^*	*T*_m_ (Δ*T*_m_ = *T*_m_ [mismatch] − *T*_m_ [match]) [°C]			
		N = A	N = G	N = C	N = U
**ON4**	5′-d(GCG TTU TTT GCT)-3′	46.5	31.9 (−14.6)	41.4 (−5.1)	32.6 (−13.9)
**ON1**	5′-d(GCG TTX TTT GCT)-3′	42.4	27.8 (−14.6)	35.1 (−7.3)	27.6 (−14.8)

*^a^* Conditions: 10 mM sodium phosphate buffer (pH 7.2) containing 100 mM NaCl and 4 μM of each oligonucleotide. *T*_m_ values are averages of at least three measurements. The sequence of ssRNA is 5′-r(AGCAAA**N**AACGC)-3′. *^b^* **X** = OxNorNA-U.

## Supplementary Materials

The following are available online, Figure S1: van’t Hoff plots of the duplexes formed between oligonucleotides (**ON1**, **ON2**, **ON4**, and **ON5**) and complementary ssDNA, Figure S2: van’t Hoff plots of the duplexes formed between oligonucleotides (**ON1**, **ON2**, **ON4**, and **ON5**) and complementary ssRNA, Table S1: *T_m_* values of duplexes formed between oligonucleotides (**ON1**, **ON2**, **ON4**, and **ON5**) and complementary ssDNA, Table S2: *T_m_* values of duplexes formed between oligonucleotides (**ON1**, **ON2**, **ON4**, and **ON5**) and complementary ssRNA. Copies of the NMR spectra of all new compounds. Copies of the HPLC and MALDI-TOF MS charts of the synthesized oligonucleotides.
