# Supplementary material for: Synthesis and Evaluation of Artificial Nucleic Acid Bearing an Oxanorbornane Scaffold
Source: Molecules. 2020 Apr 9;25(7):1732. doi: 10.3390/molecules25071732 (PMC7180610; doi:10.3390/molecules25071732)

Supplementary Material

for

Synthesis and evaluation of artificial nucleic acid bearing an oxanorbornane scaffold

Hibiki Komine [1,†], Shohei Mori [1,†], Kunihiko Morihiro [1,2,#,*], Kenta Ishida [1], Takumi Okuda [1], Yuuya Kasahara [1,2], Hiroshi Aoyama [1], Takao Yamaguchi [1,*] and Satoshi Obika [1,2,*]

[1] Graduate School of Pharmaceutical Sciences, Osaka University, 1-6 Yamadaoka, Suita, Osaka 565-0871, Japan

[2] National Institutes of Biomedical Innovation, Health and Nutrition (NIBIOHN), 7-6-8 Saito-Asagi, Ibaraki, Osaka 567-0085, Japan

* Correspondence: morihiro@bioorg.rcast.u-tokyo.ac.jp (K.M.); yamaguchi-ta@phs.osaka-u.ac.jp (T.Y.); obika@phs.osaka-u.ac.jp (S.O.); Tel.: +81-6-6879-8200 (S.O.)

[#] Current address: Department of Chemistry and Biotechnology, Graduate School of Engineering, The University of Tokyo, 7-3-1 Hongo, Bunkyo-ku, Tokyo 113-8656, Japan

[†] Both authors should be considered as first authors.

Contents

1. Supplementary results (van't Hoff plots)
2. Copies of the NMR spectra of all new compounds
3. Copies of the HPLC and MALDI-TOF MS charts of the synthesized oligonucleotides

1. Supplementary results

Table S1. T_m values of duplexes formed between oligonucleotides (**ON1**, **ON2**, **ON4**, and **ON5**) and complementary ssDNA

	Sequence	T_m [°C] values at each concentration					
		0.9 μM	1.48 μM	2.44 μM	4.00 μM	6.52 μM	13.6 μM
**ON4**	5'-d(GCG TTU TTT GCT)-3'	46.5	48.4	49.0	49.9	50.7	52.0
**ON1**	5'-d(GCG TT**X** TTT GCT)-3'	40.1	41.2	43.0	45.4	45.6	46.8
**ON5**	5'-d(GCG UTU TUT GCT)-3'	45.0	45.9	46.9	48.4	49.3	51.9
**ON2**	5'-d(GCG **X**T**X** T**X**T GCT)-3'	14.1	15.9	17.5	21.0	22.2	24.7

Conditions: 10 mM sodium phosphate buffer (pH 7.2), 100 mM NaCl aq., and 0.90–13.6 μM each oligonucleotide (six data points). The T_m values reflect the average of at least three measurements. The sequence of the target ssDNA is 5′-d(AGCAAAAAACGC)-3′. U = 2′-deoxyuridine, X = OxNorNA-U.

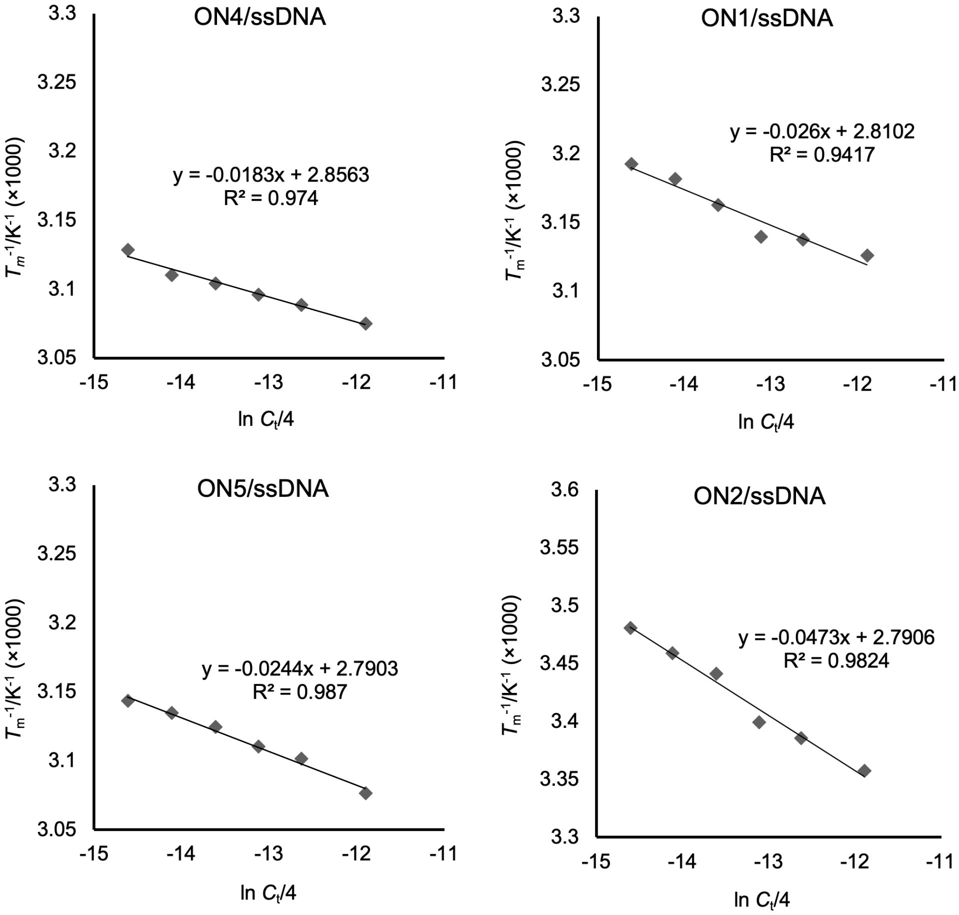

Figure S1. Van't Hoff plots of the duplexes formed between oligonucleotides (**ON1**, **ON2**, **ON4**, and **ON5**) and complementary ssDNA.

Table S2. T_m values of duplexes formed between oligonucleotides (**ON1**, **ON2**, **ON4**, and **ON5**) and complementary ssRNA

	Sequence	T_m [°C] values at each concentration					
		0.9 μM	1.48 μM	2.44 μM	4.00 μM	6.52 μM	13.6 μM
**ON4**	5'-d(GCG TTU TTT GCT)-3'	43.3	45.0	46.0	46.5	47.6	49.6
**ON1**	5'-d(GCG TTX TTT GCT)-3'	38.9	39.3	41.2	42.4	43.1	44.3
**ON5**	5'-d(GCG UTU TUT GCT)-3'	40.8	42.5	44.4	44.7	46.1	47.1
**ON2**	5'-d(GCG XTX TXT GCT)-3'	18.9	20.1	21.9	24.7	25.0	26.9

Conditions: 10 mM sodium phosphate buffer (pH 7.2), 100 mM NaCl aq., and 0.90–13.6 μM each oligonucleotide (six data points). The T_m values reflect the average of at least three measurements. The sequence of the target ssRNA is 5′-r(AGCAAAAAACGC)-3′. U = 2′-deoxyuridine, X = OxNorNA-U.

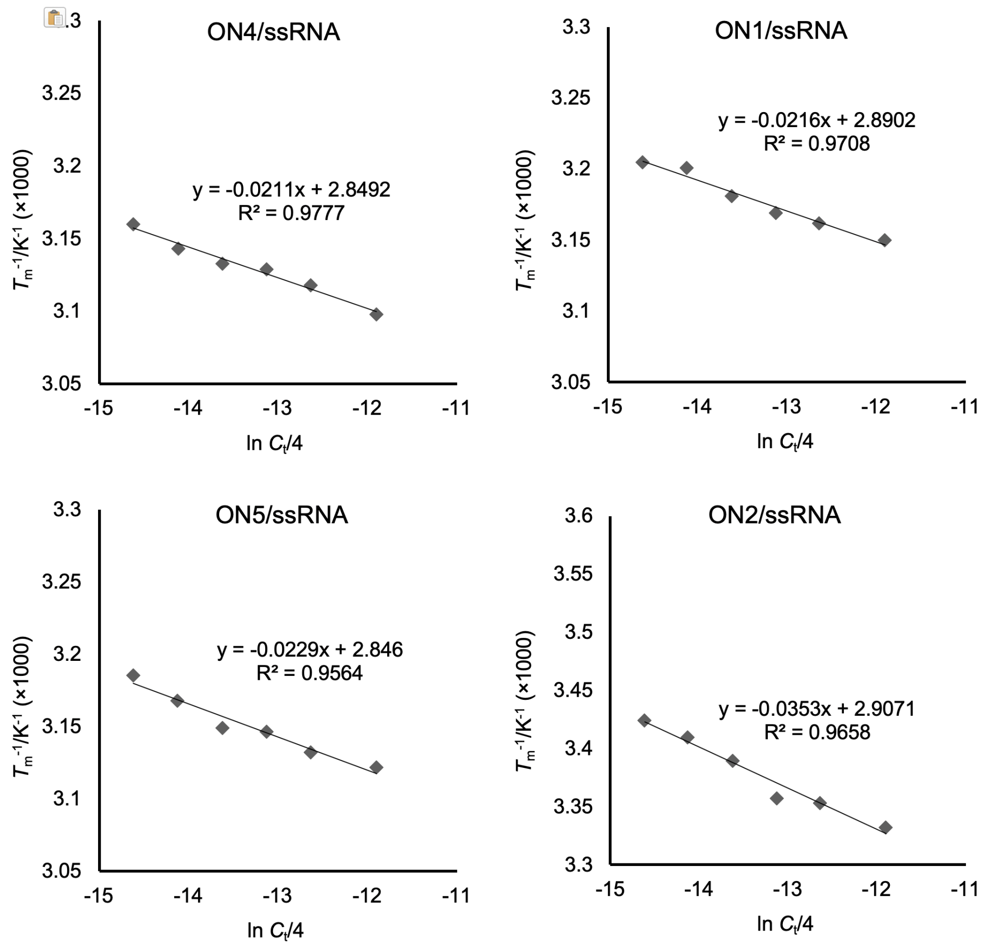

Figure S2. Van't Hoff plots of the duplexes formed between oligonucleotides (**ON1**, **ON2**, **ON4**, and **ON5**) and complementary ssRNA.

2. Copies of the ^{1}H, ^{13}C, and ^{31}P NMR spectra of all new compounds

Compound **2** (^{1}H NMR, $CDCl_3$, 500MHz)

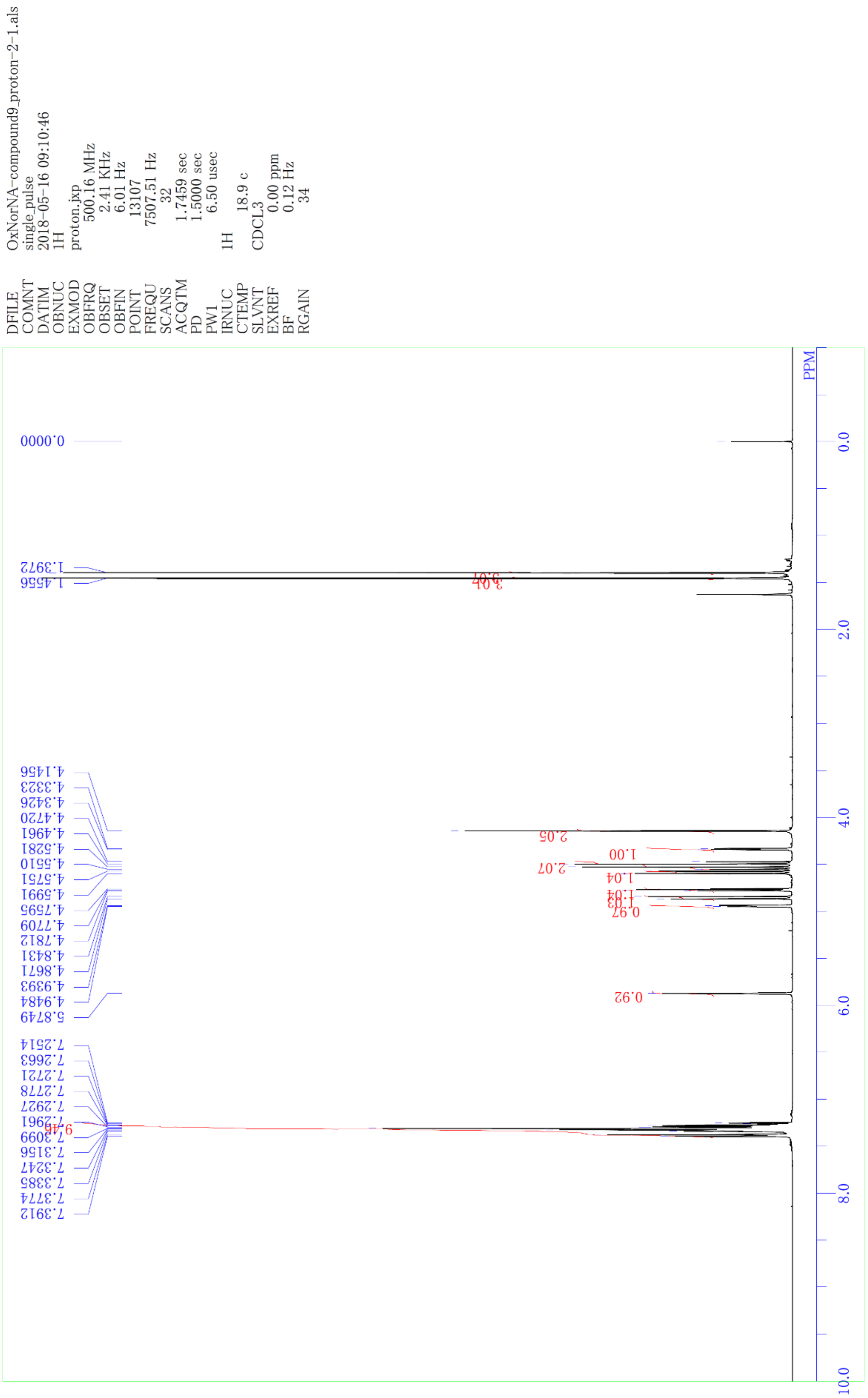

Compound **2** (^{13}C NMR, $CDCl_3$, 125MHz)

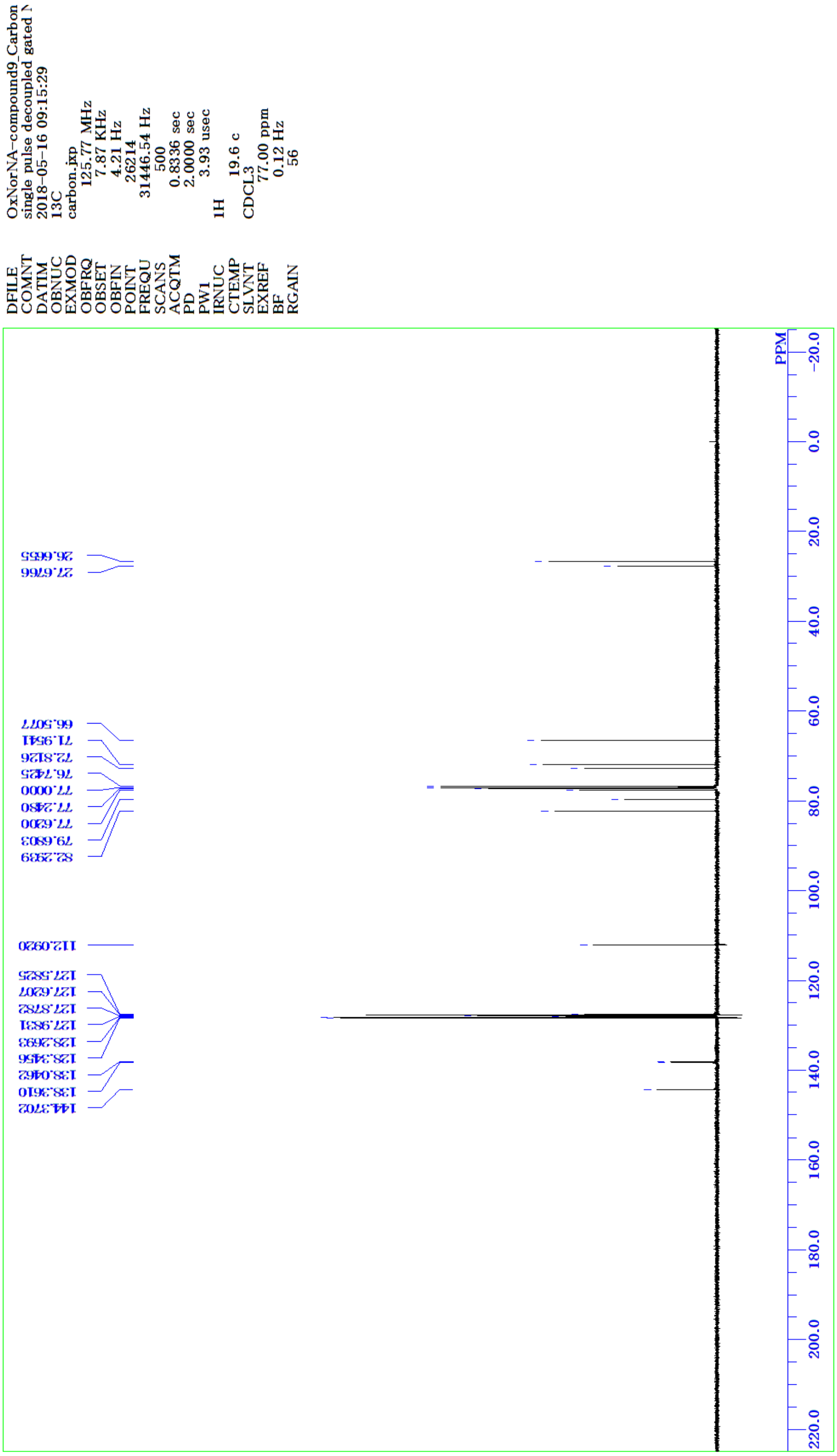

Compound **3** (^{1}H NMR, $CDCl_3$, 500MHz)

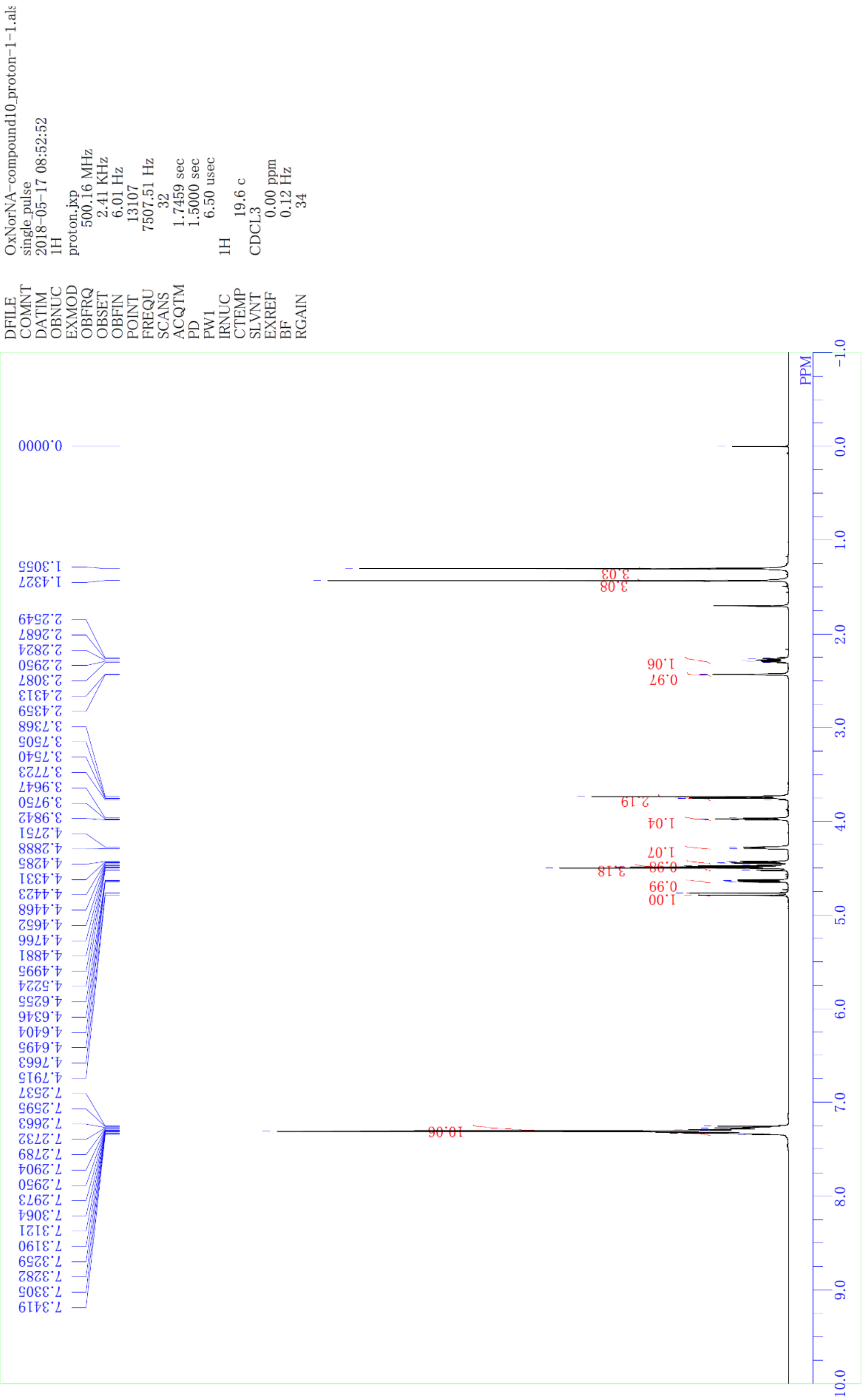

Compound **3** (^{13}C NMR, $CDCl_3$, 125MHz)

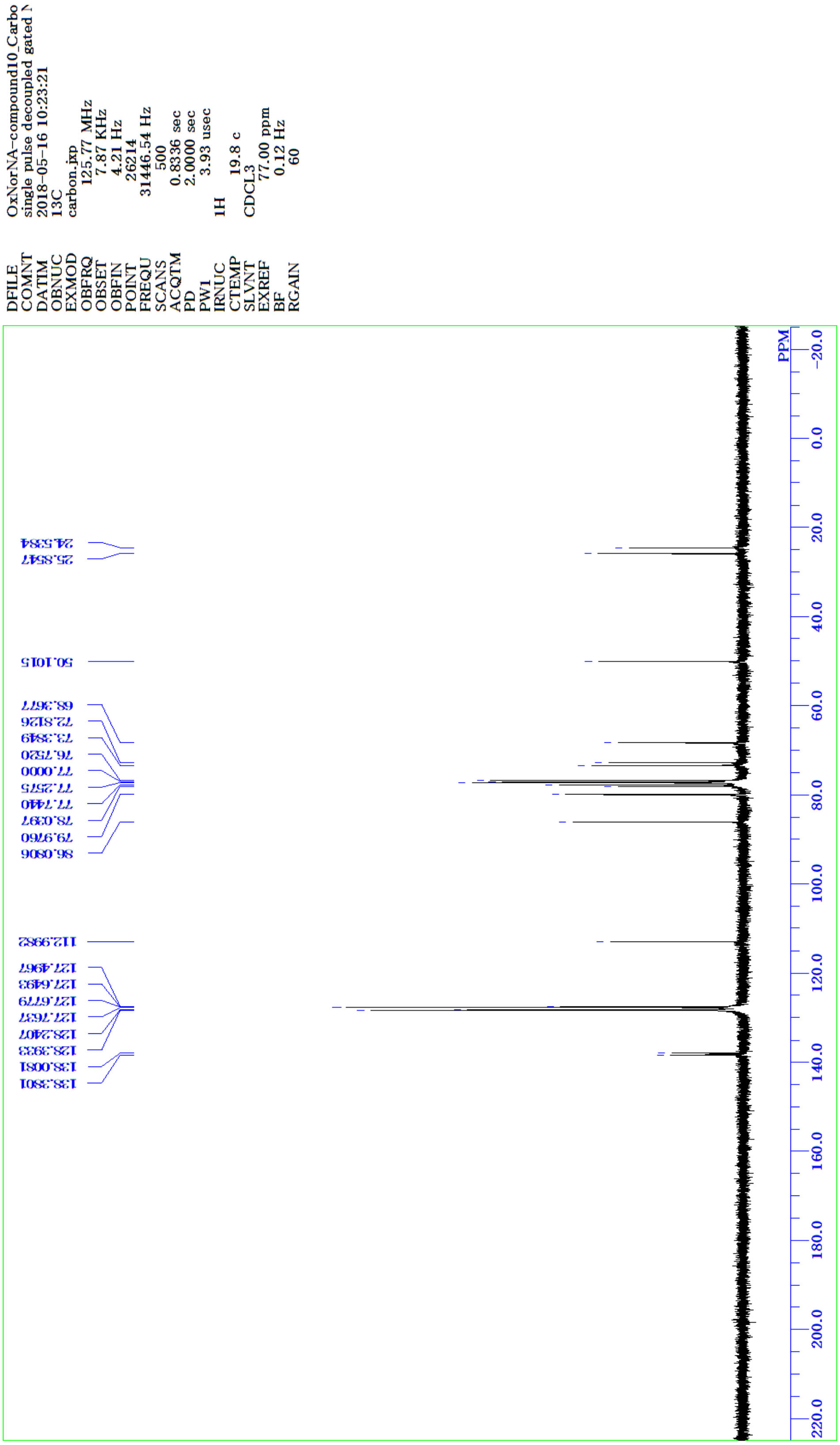

Compound **4** (^1H NMR, $CDCl_3$, 500MHz)

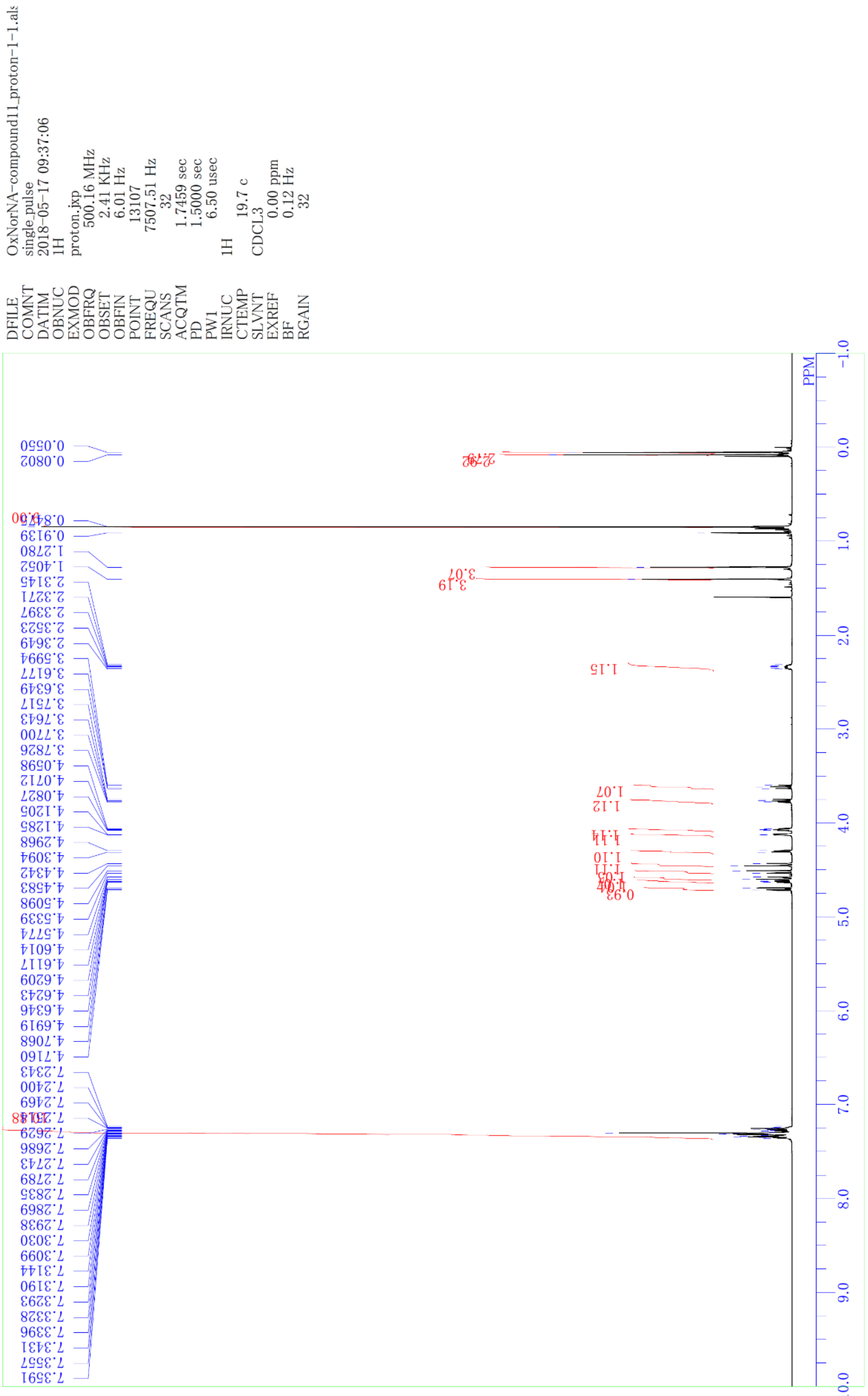

Compound **4** (^{13}C NMR, $CDCl_3$, 125MHz)

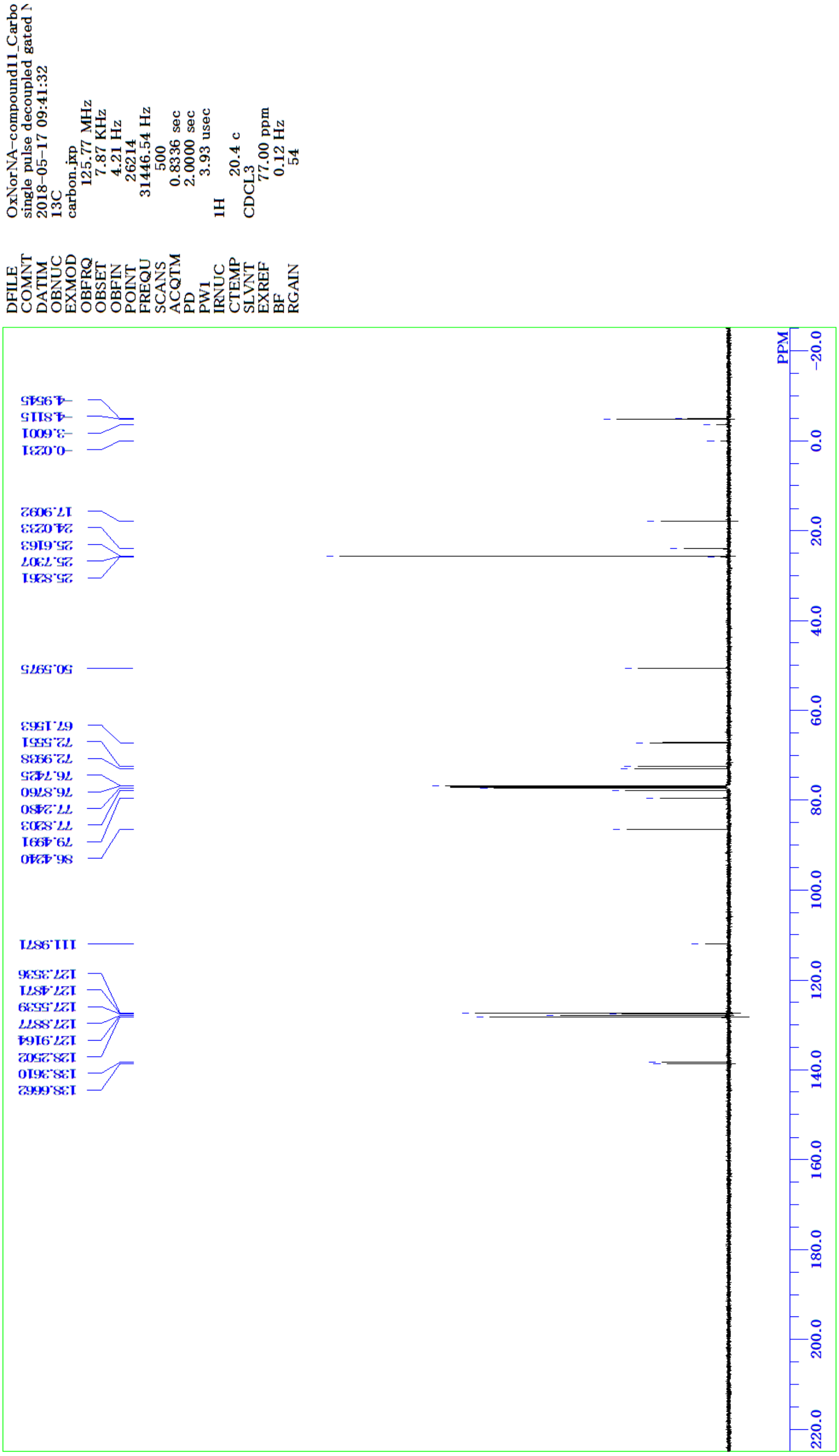

Compound **5** (^{1}H NMR, $CDCl_3$, 500MHz)

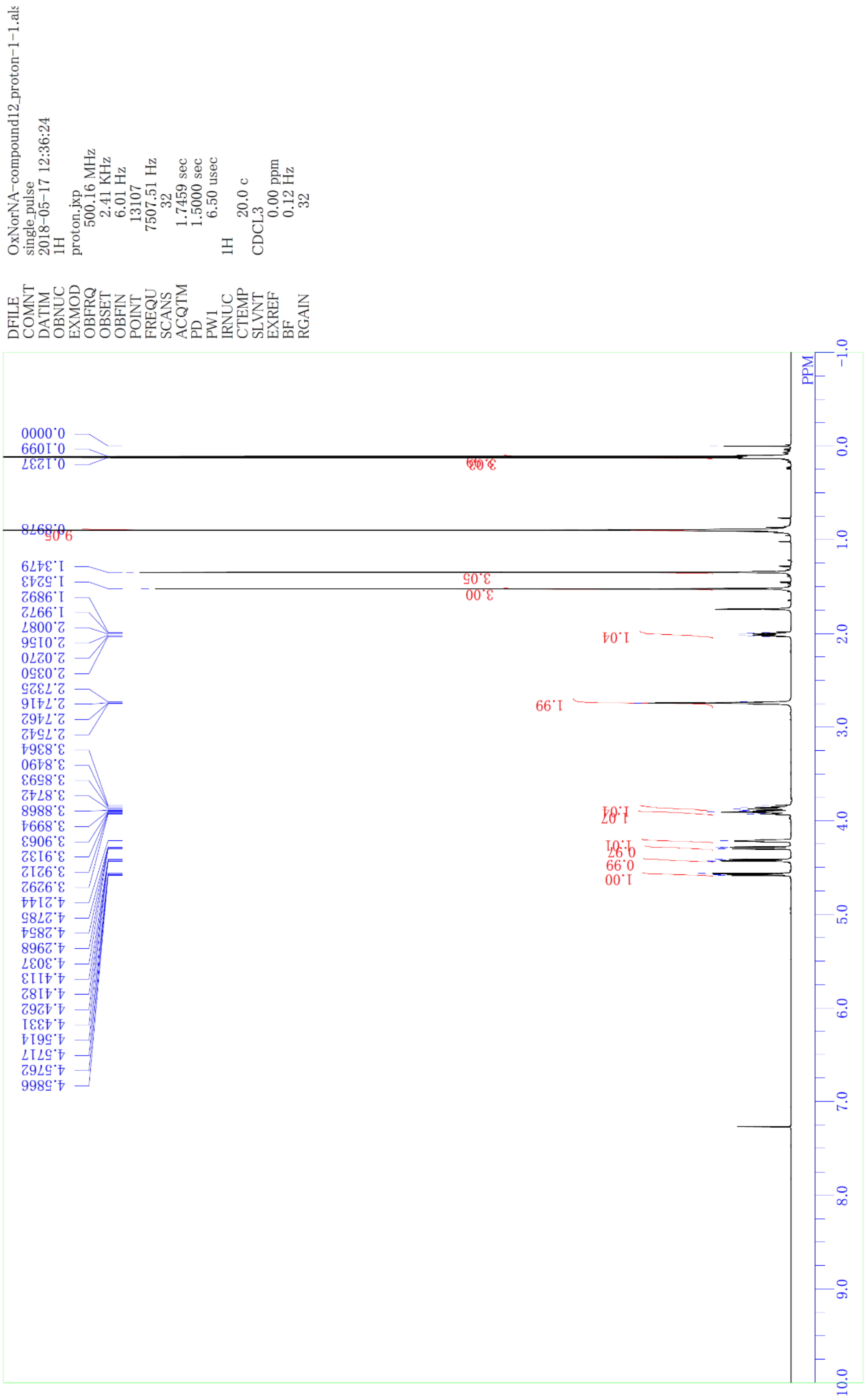

Compound **5** (^{13}C NMR, $CDCl_3$, 125MHz)

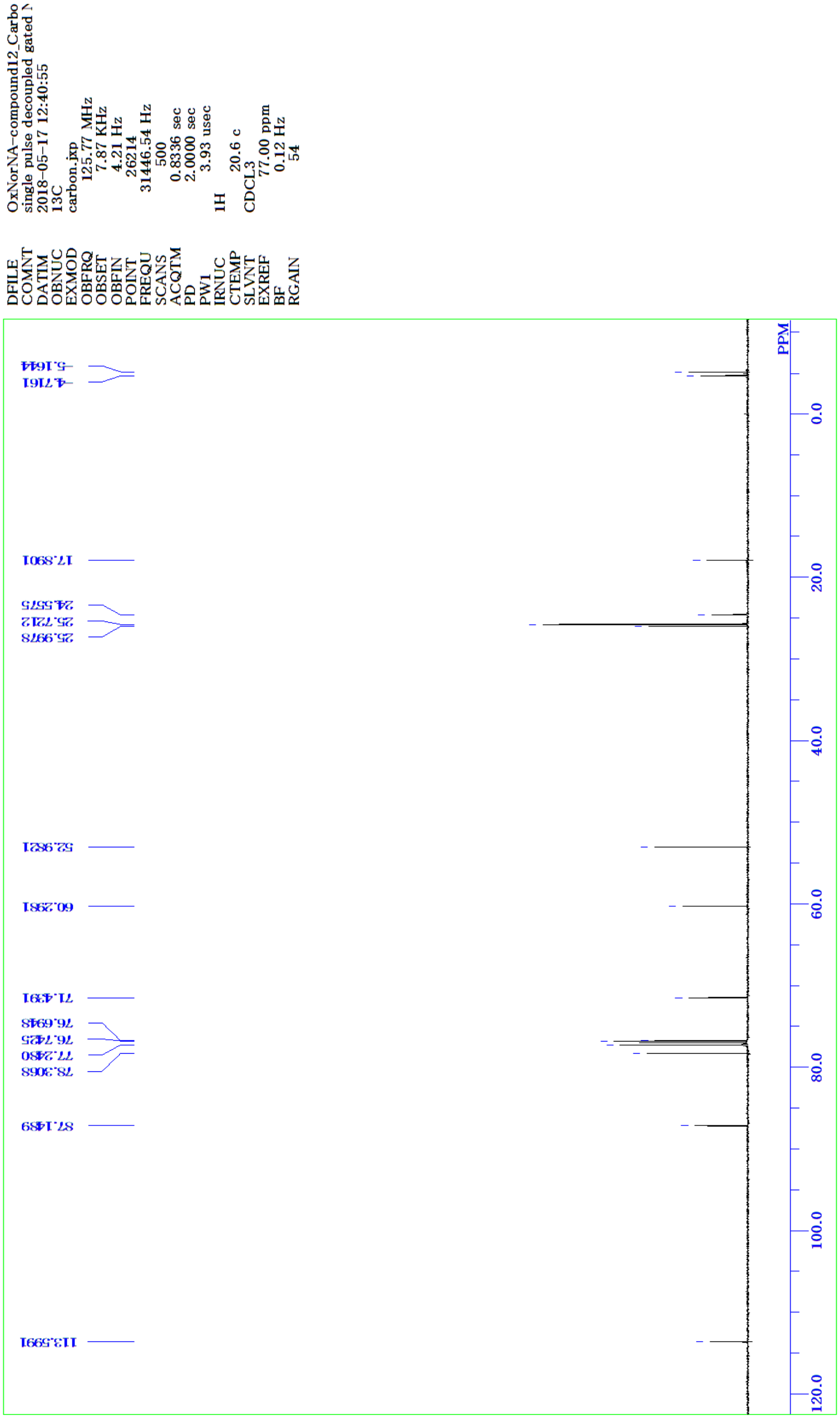

Compound **6** (^{1}H NMR, $CDCl_3$, 500MHz)

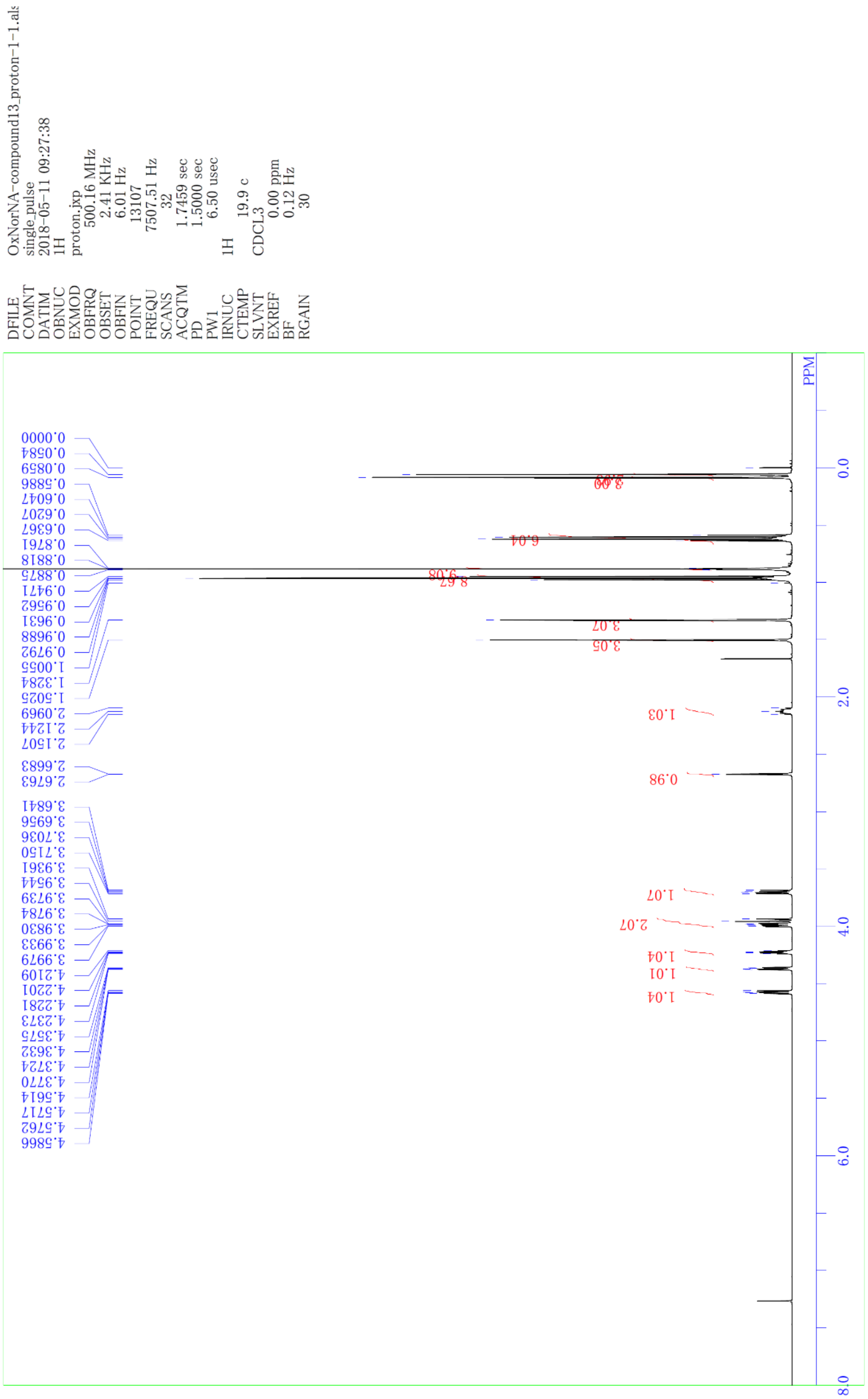

Compound **6** (^{13}C NMR, $CDCl_3$, 125MHz)

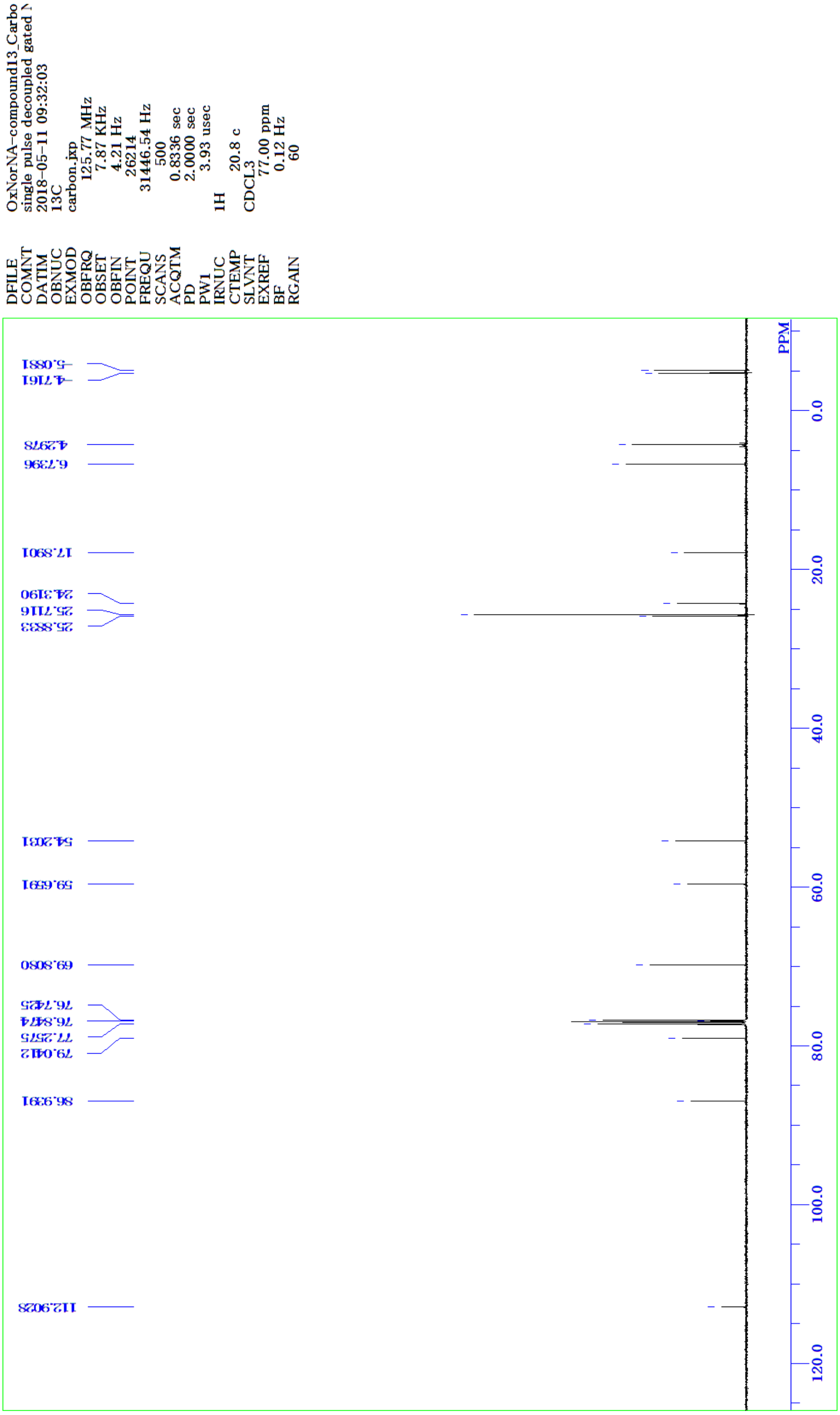

Compound **7** (^{1}H NMR, $CDCl_3$, 500MHz)

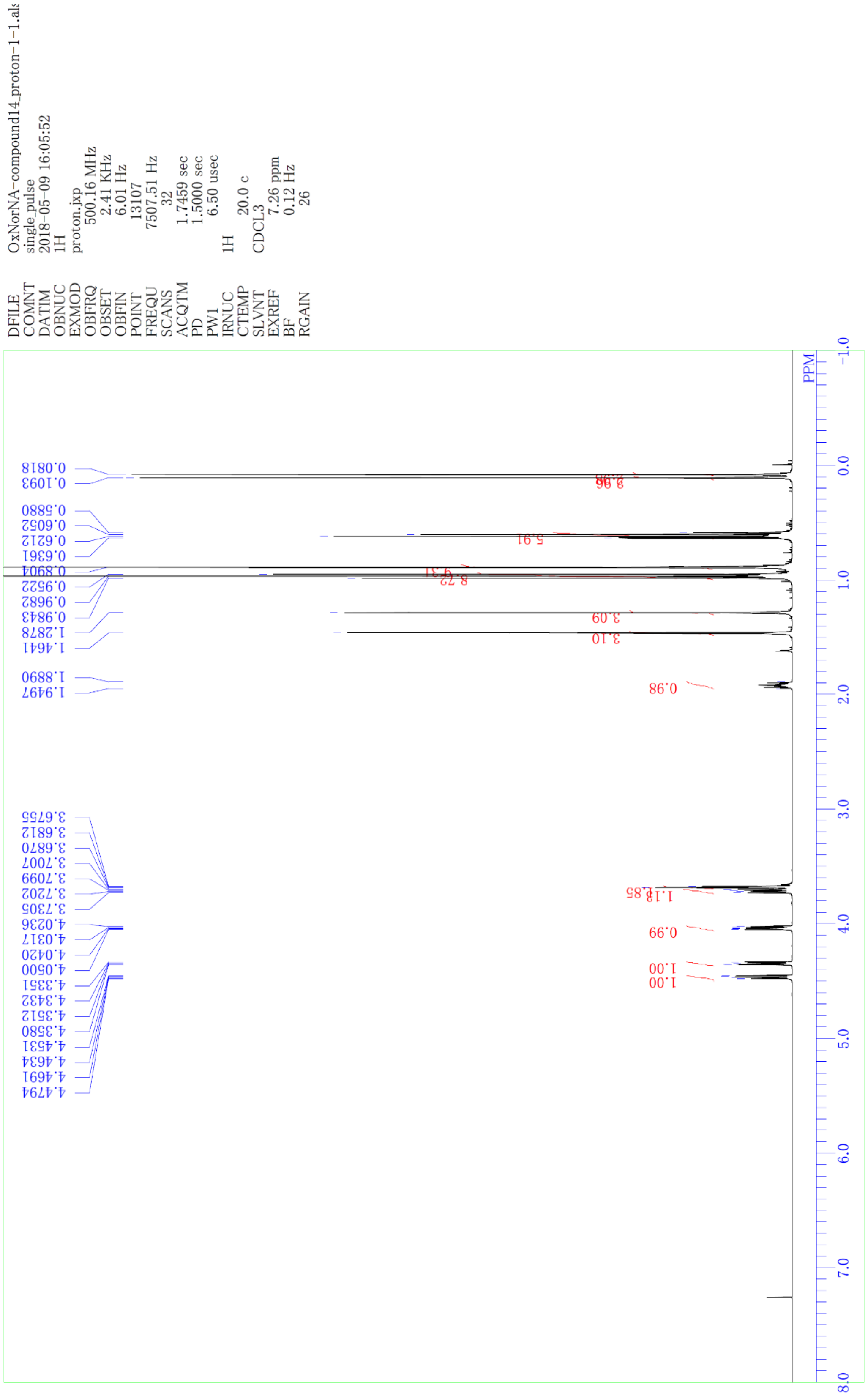

Compound **7** (^{13}C NMR, $CDCl_3$, 125MHz)

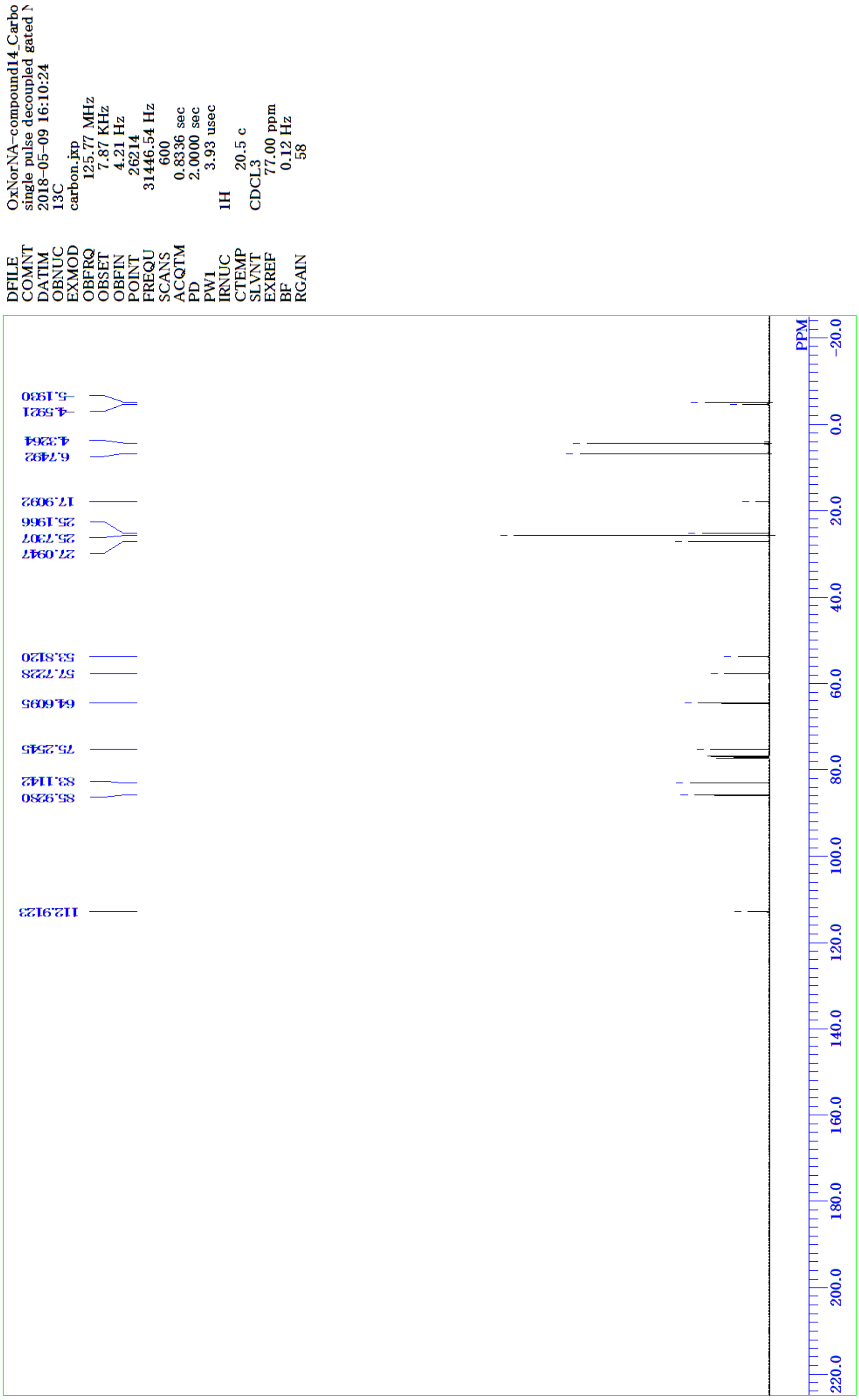

Compound **8** (^{1}H NMR, DMSO-d_6, 500MHz)

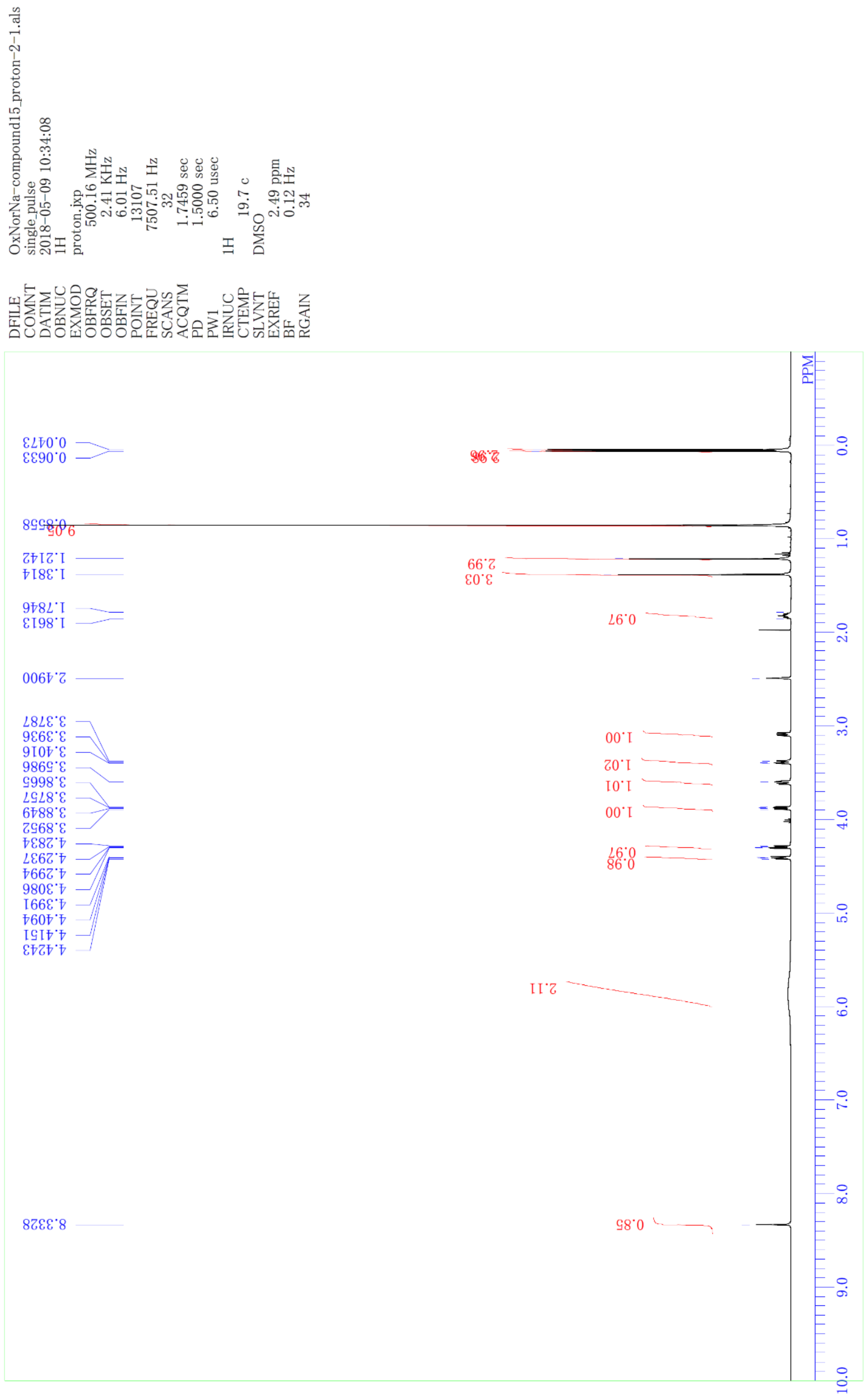

Compound **8** (^{13}C NMR, DMSO-d_6, 125MHz)

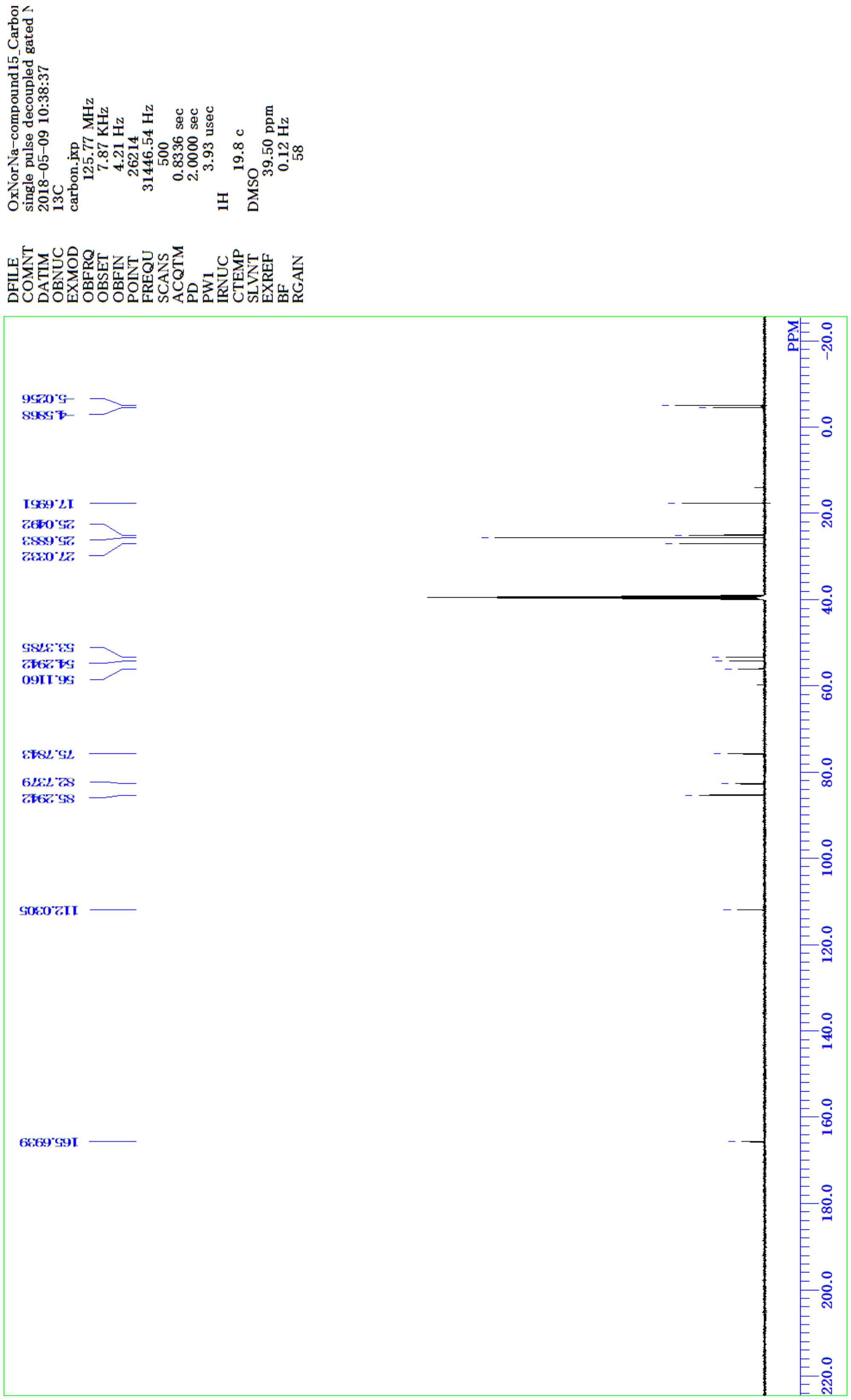

Compound **9** (^{1}H NMR, $CDCl_3$, 500MHz)

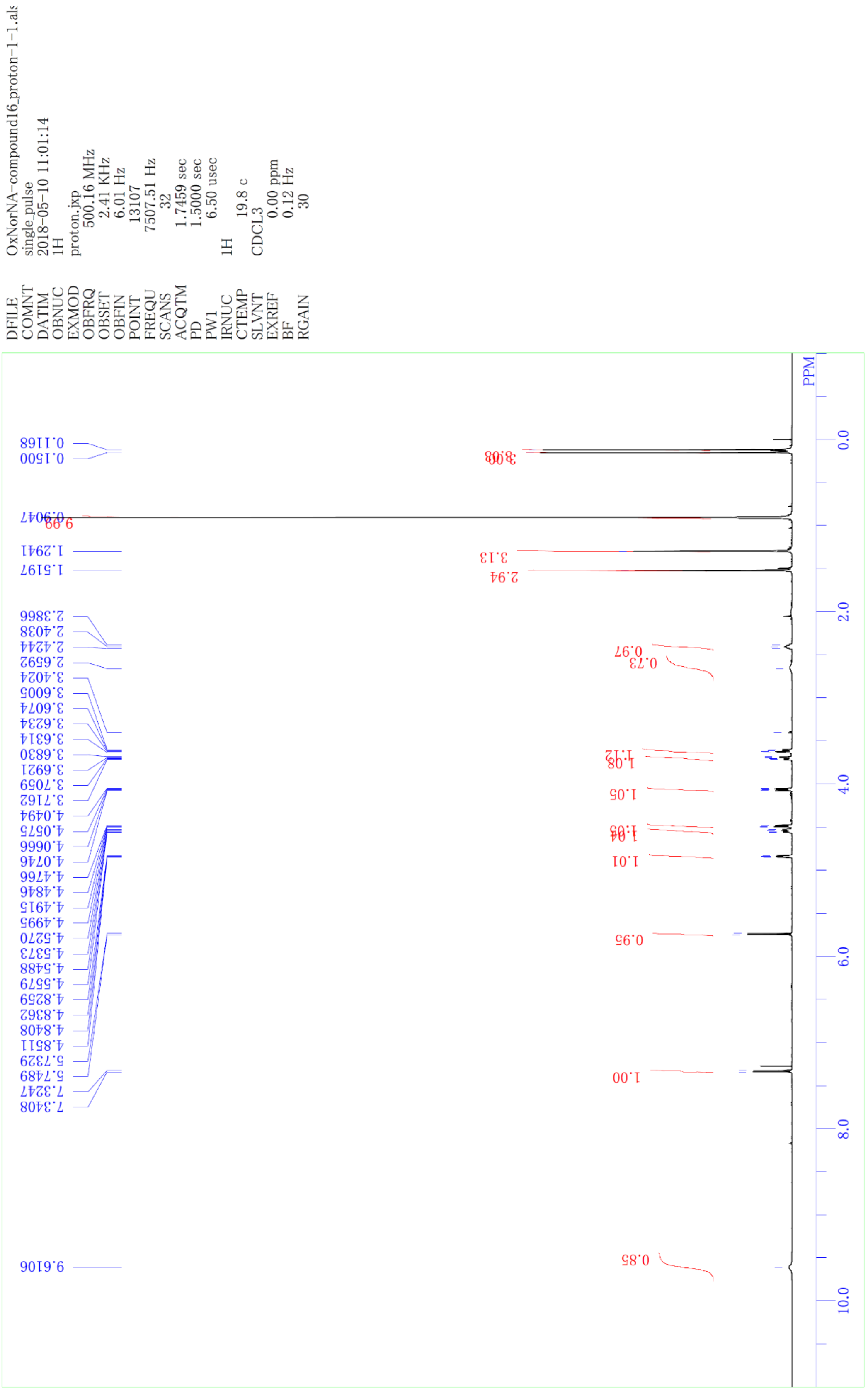

Compound **9** (^{13}C NMR, $CDCl_3$, 125MHz)

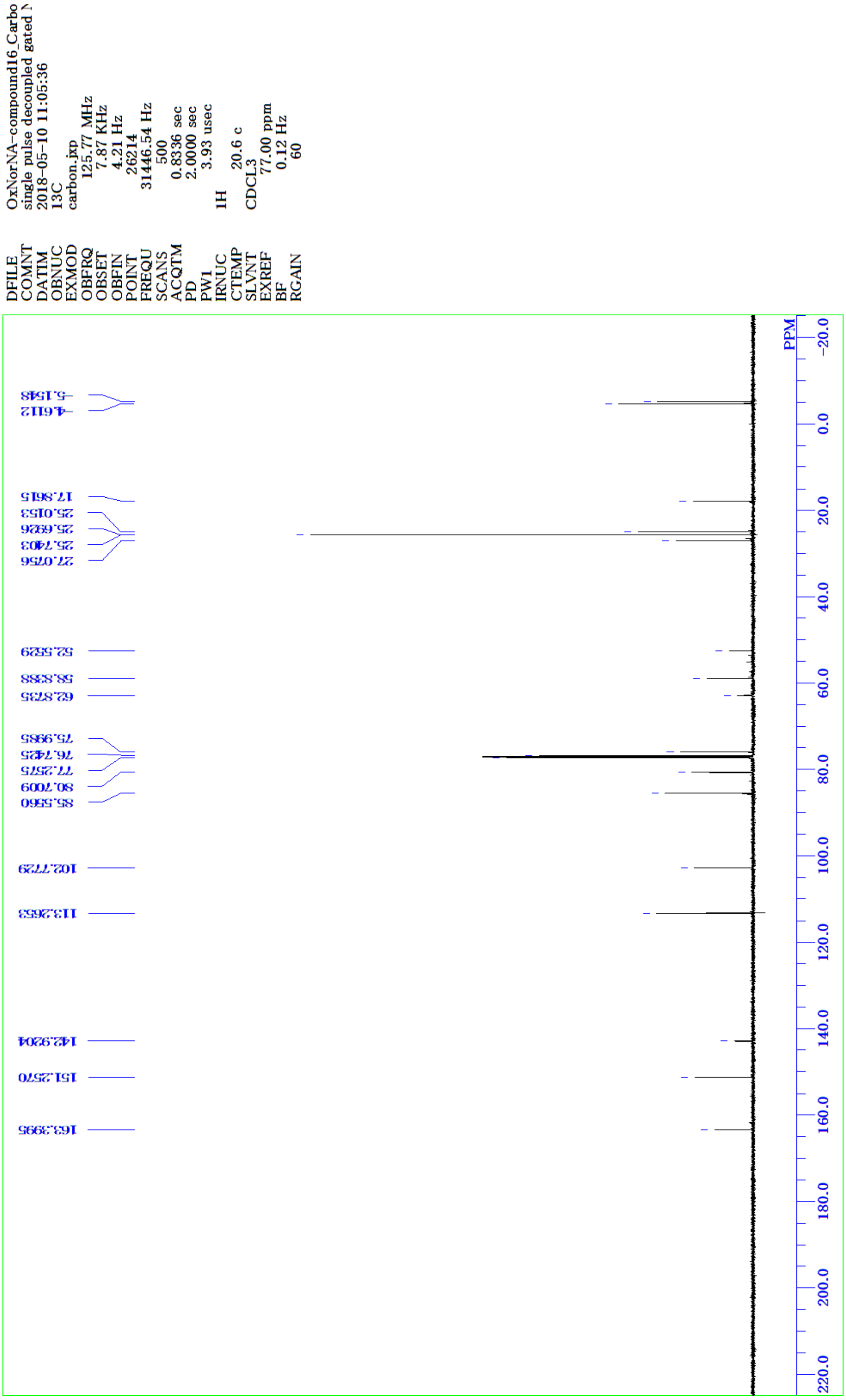

Compound **10** (^{1}H NMR, DMSO-d_6, 500MHz)

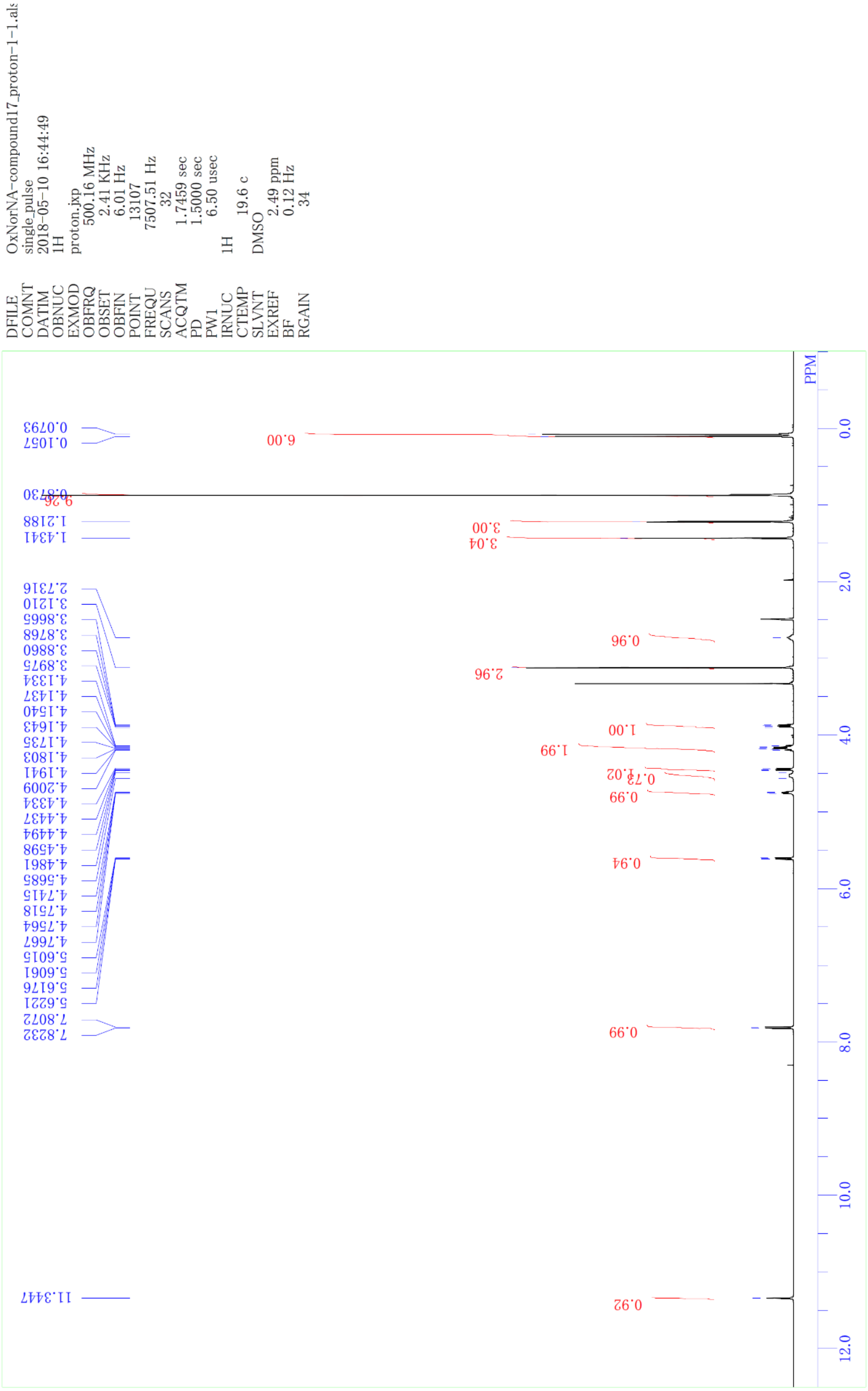

Compound **10** (^{13}C NMR, DMSO-d_6, 125MHz)

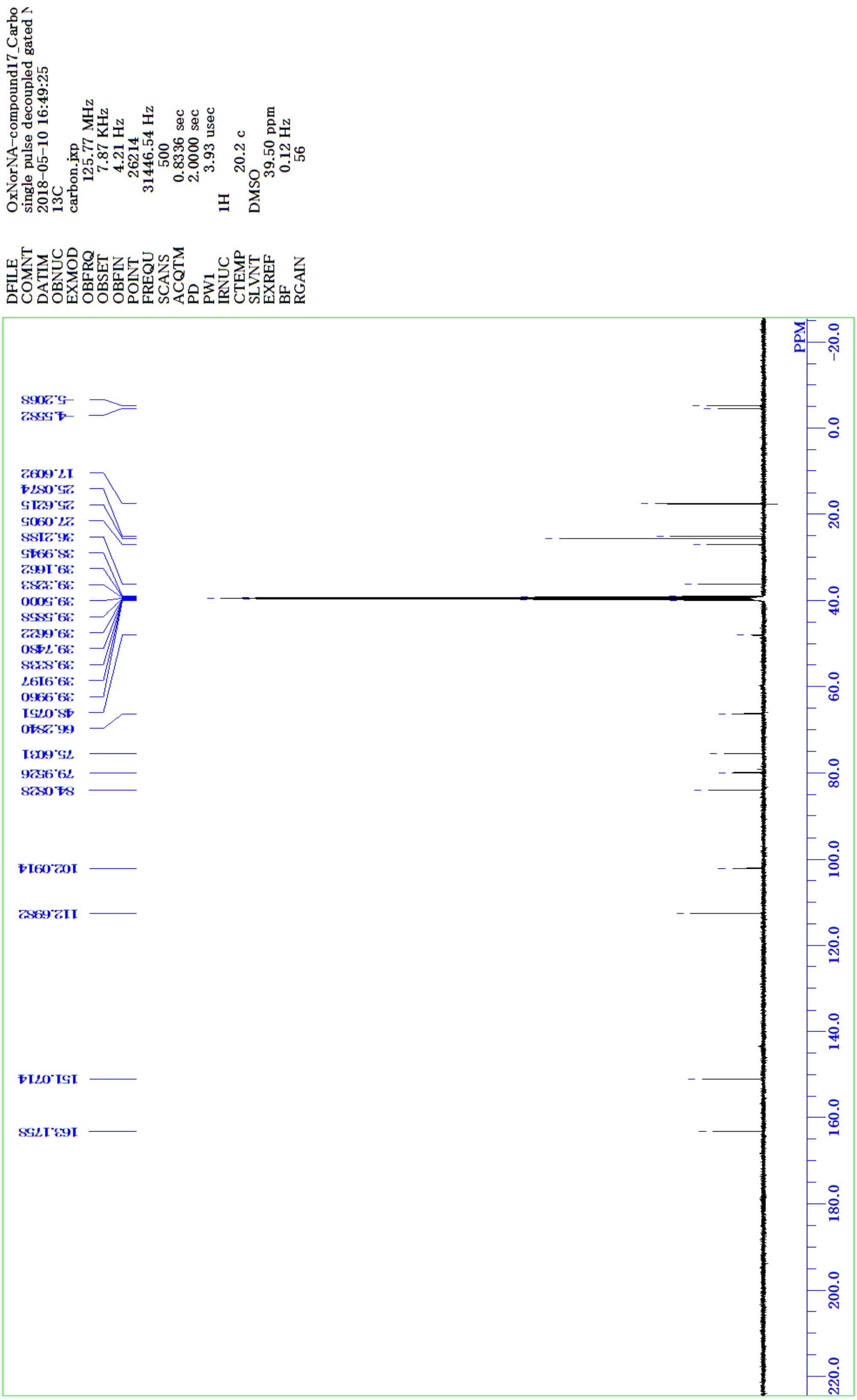

Compound **11** (^{1}H NMR, DMSO-d_6, 500MHz)

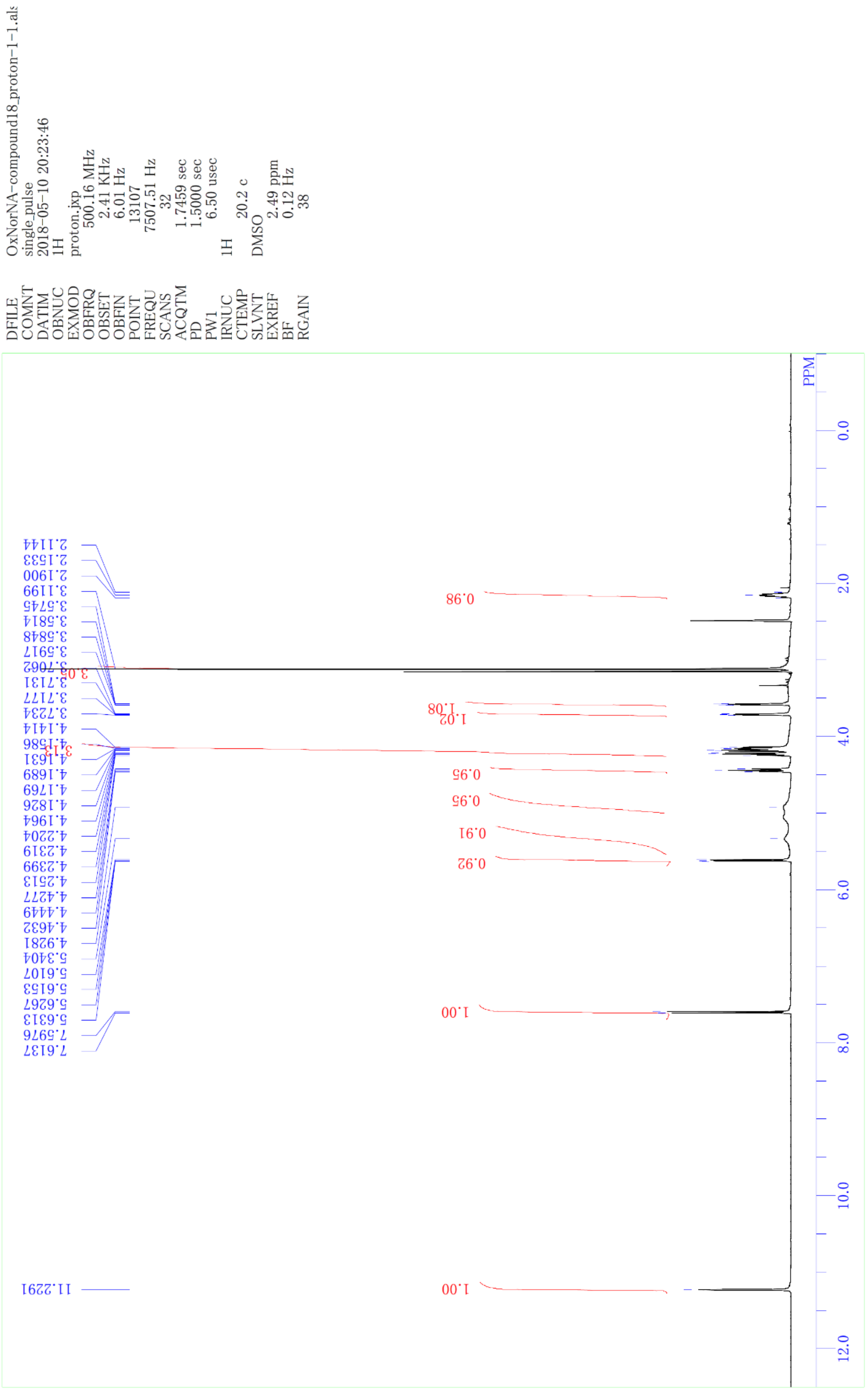

Compound **11** (^{13}C NMR, DMSO-d_6, 125MHz**)**

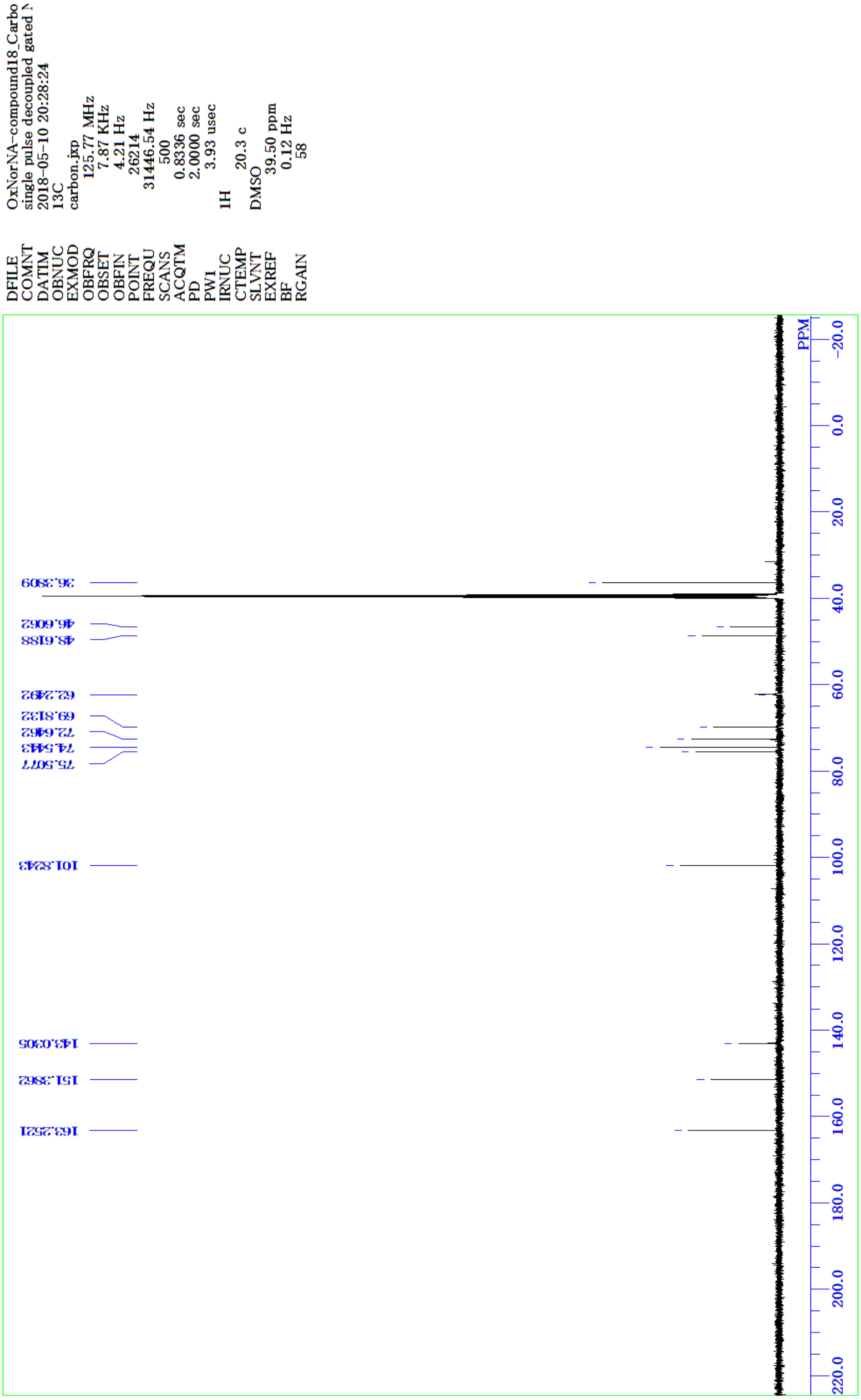

Compound **11** (^{1}H–^{1}H COSY NMR, DMSO-d_6, 300MHz)

```
DATIM   29-05-2018 21:22:22
DFILE   OxNorNA-Compound19_cosy
OBNUC   1H
EXMOD   cosy.jxp
OFR     300.53 MHz
OBSET   1.15 KHz
OBFIN   8.57 Hz
POINT   1024
FREQU   4508.57 Hz
SCANS   4
ACQTM   0.2271 sec
PD      1.5000 sec
PW1     11.00 usec
IRN
CTEMP   20.9 c
SLVNT   DMSO
EXREF   2.49 ppm
BF      4.20 Hz
RGAIN   44
```

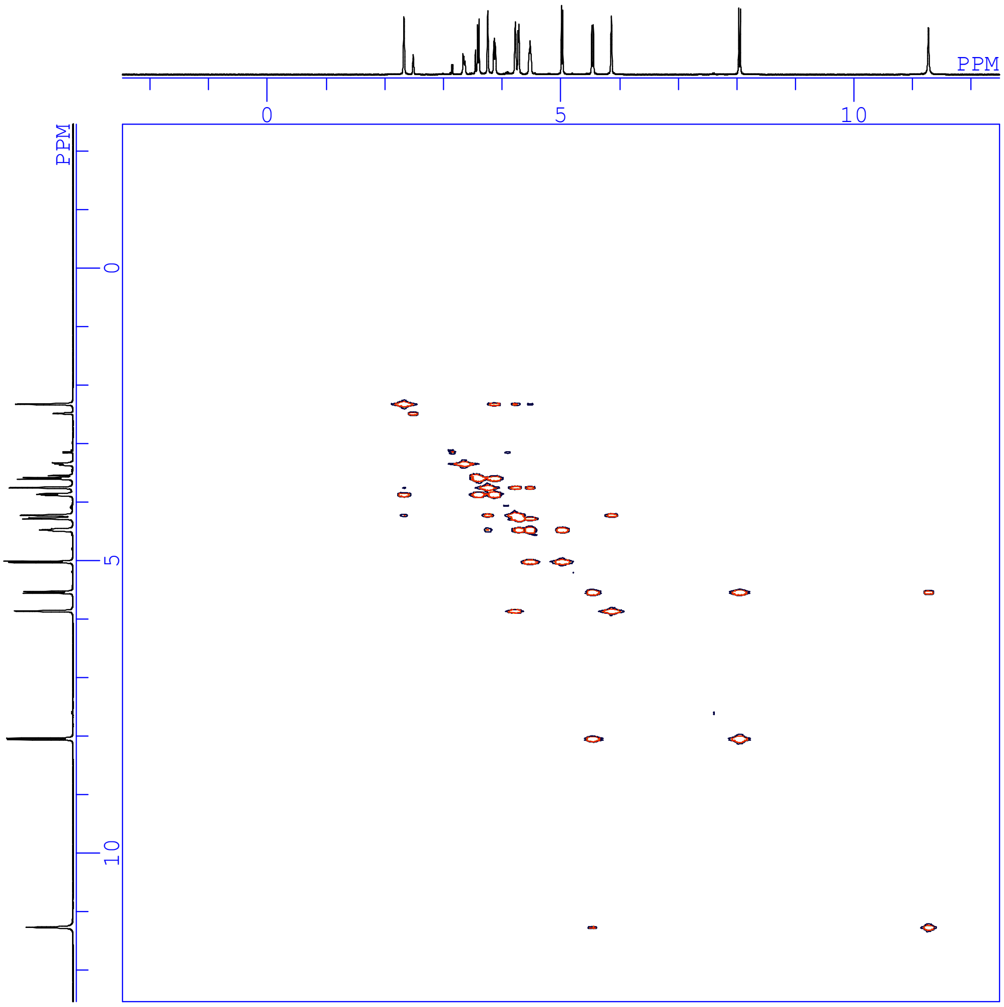

Compound **11** (DEPT NMR, DMSO-d_6, 76MHz)

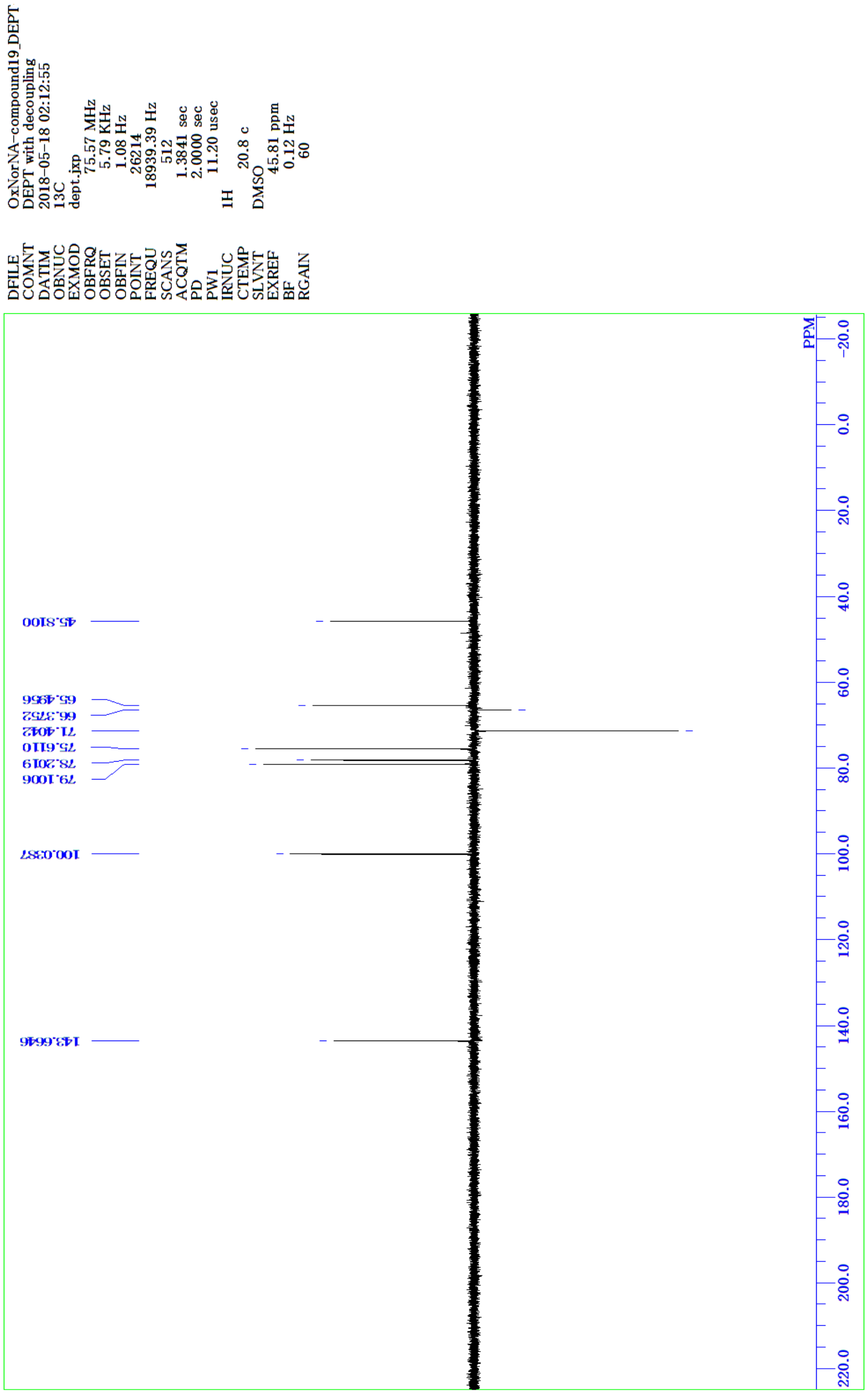

Compound **11** (HMQC NMR, DMSO-d_6, 300MHz)

DATIM	18-05-2018 03:14:34	
DFILE	OxNorNA-compound19_HMQC	
OBNUC	1H	
EXMOD	hmqc.jxp	
OFR	300.53	MHz
OBSET	1.15	KHz
OBFIN	8.57	Hz
POINT	819	
FREQU	4510.60	Hz
SCANS	4	
ACQTM	0.1816	sec
PD	1.5000	sec
PW1	11.00	usec
IRN		
CTEMP	20.6	c
SLVNT	DMSO	
EXREF	2.49	ppm
BF	4.20	Hz
RGAIN	84	

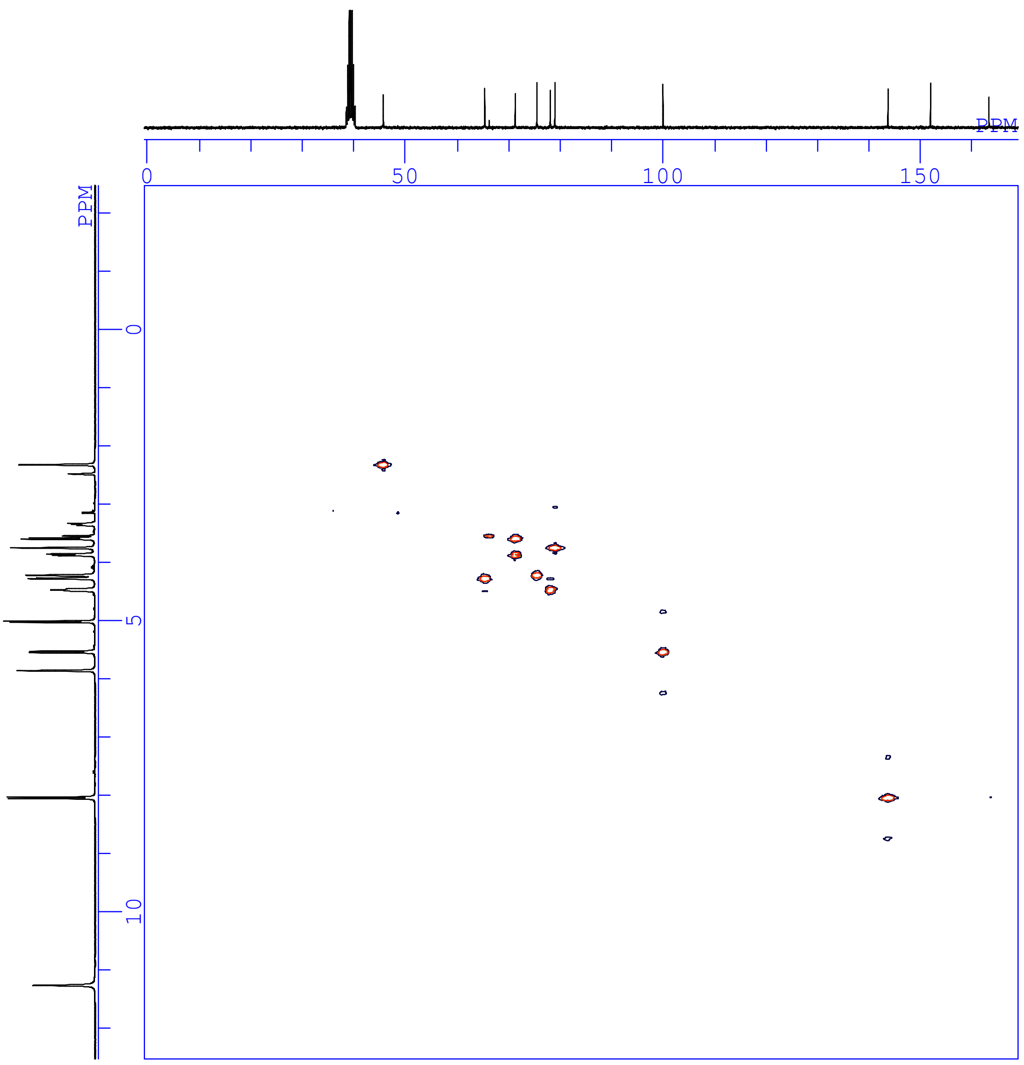

Compound **11** (HMBC NMR, DMSO-d_6, 300MHz)

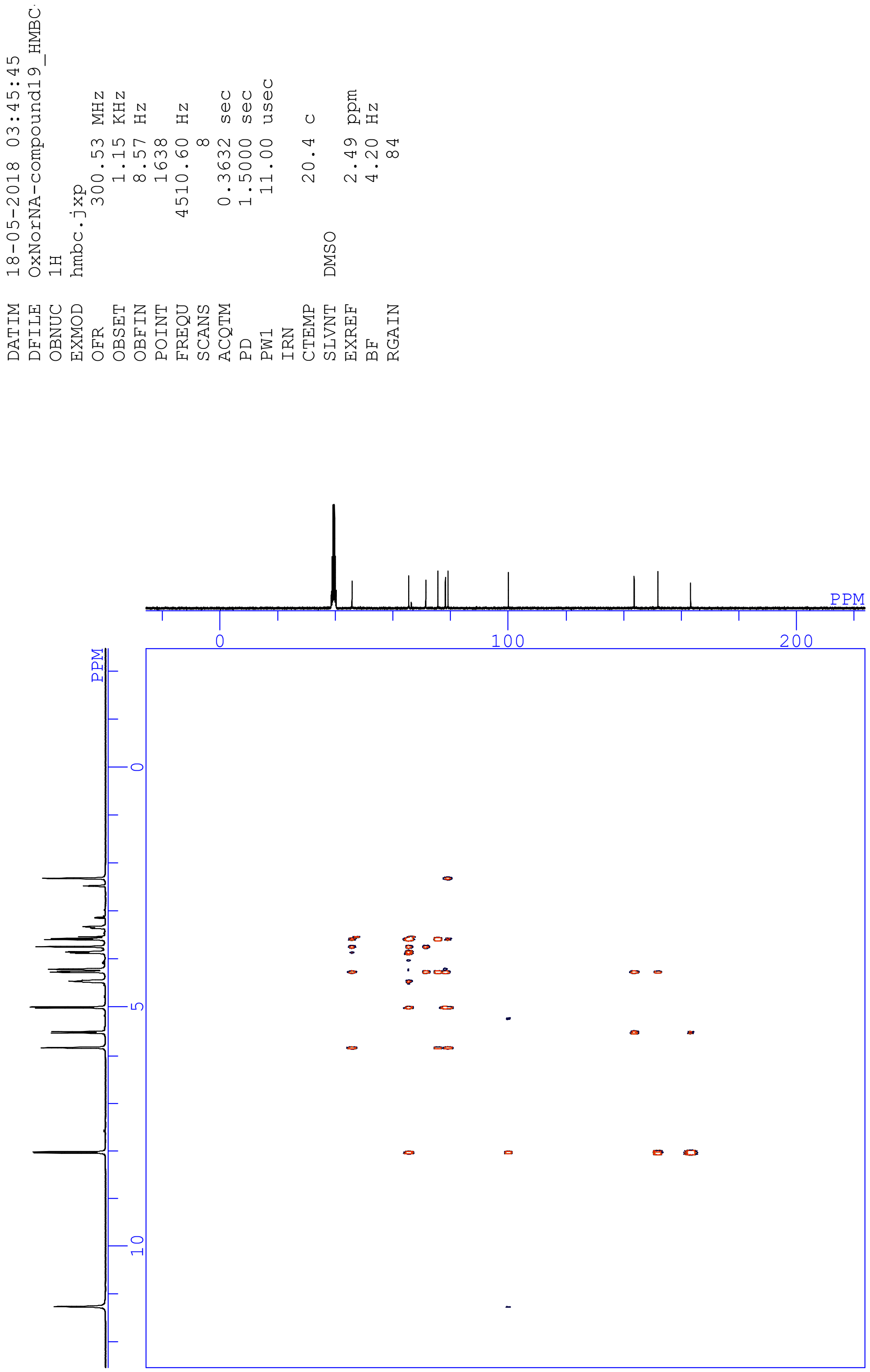

Compound **11** (NOESY NMR, DMSO-d_6, 300MHz**)**

DATIM	18-05-2018 04:52:42	
DFILE	OxNorNA-compound19_NOESY	
OBNUC	1H	
EXMOD	noesy.jxp	
OFR	300.53	MHz
OBSET	1.15	KHz
OBFIN	8.57	Hz
POINT	819	
FREQU	4510.60	Hz
SCANS	8	
ACQTM	0.1816	sec
PD	1.5000	sec
PW1	11.00	usec
IRN		
CTEMP	20.5	c
SLVNT	DMSO	
EXREF	2.49	ppm
BF	4.20	Hz
RGAIN	46	

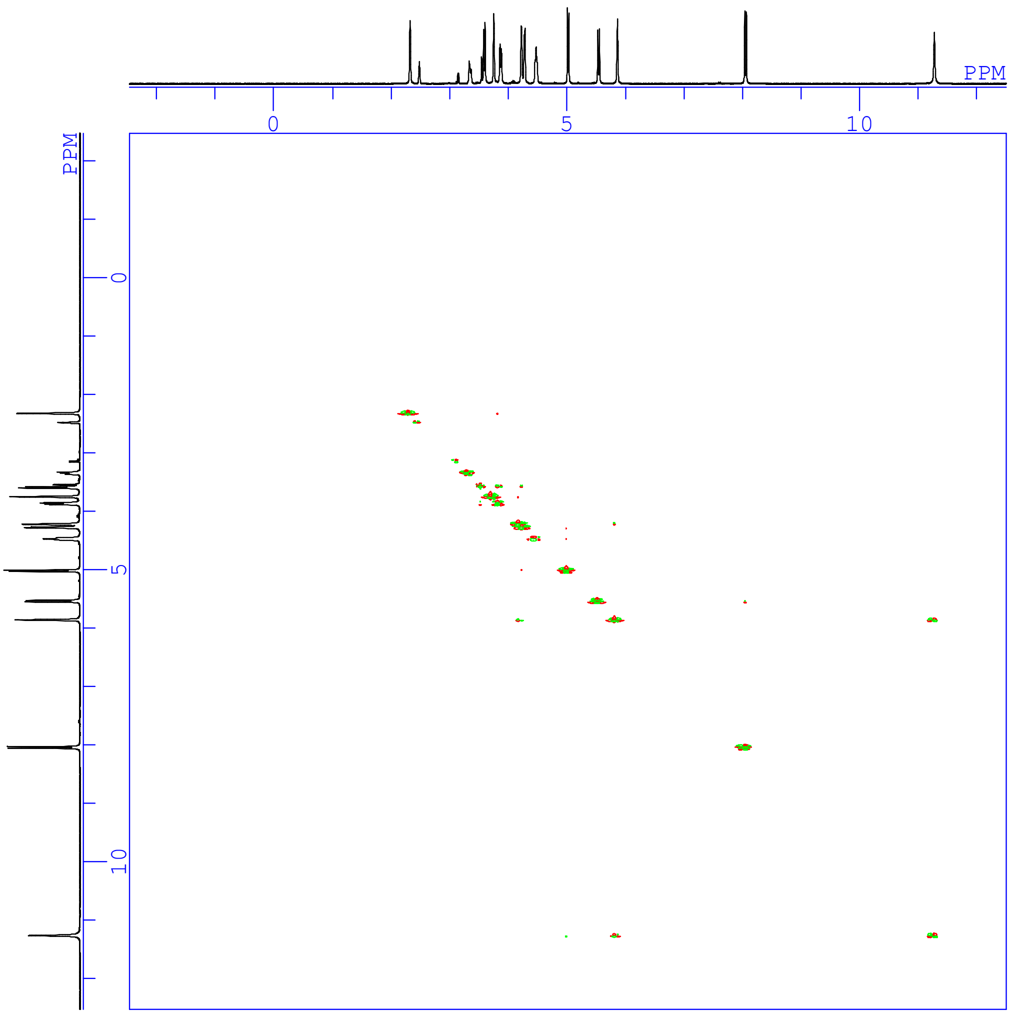

Compound **12** (^{1}H NMR, DMSO-d_6, 500MHz)

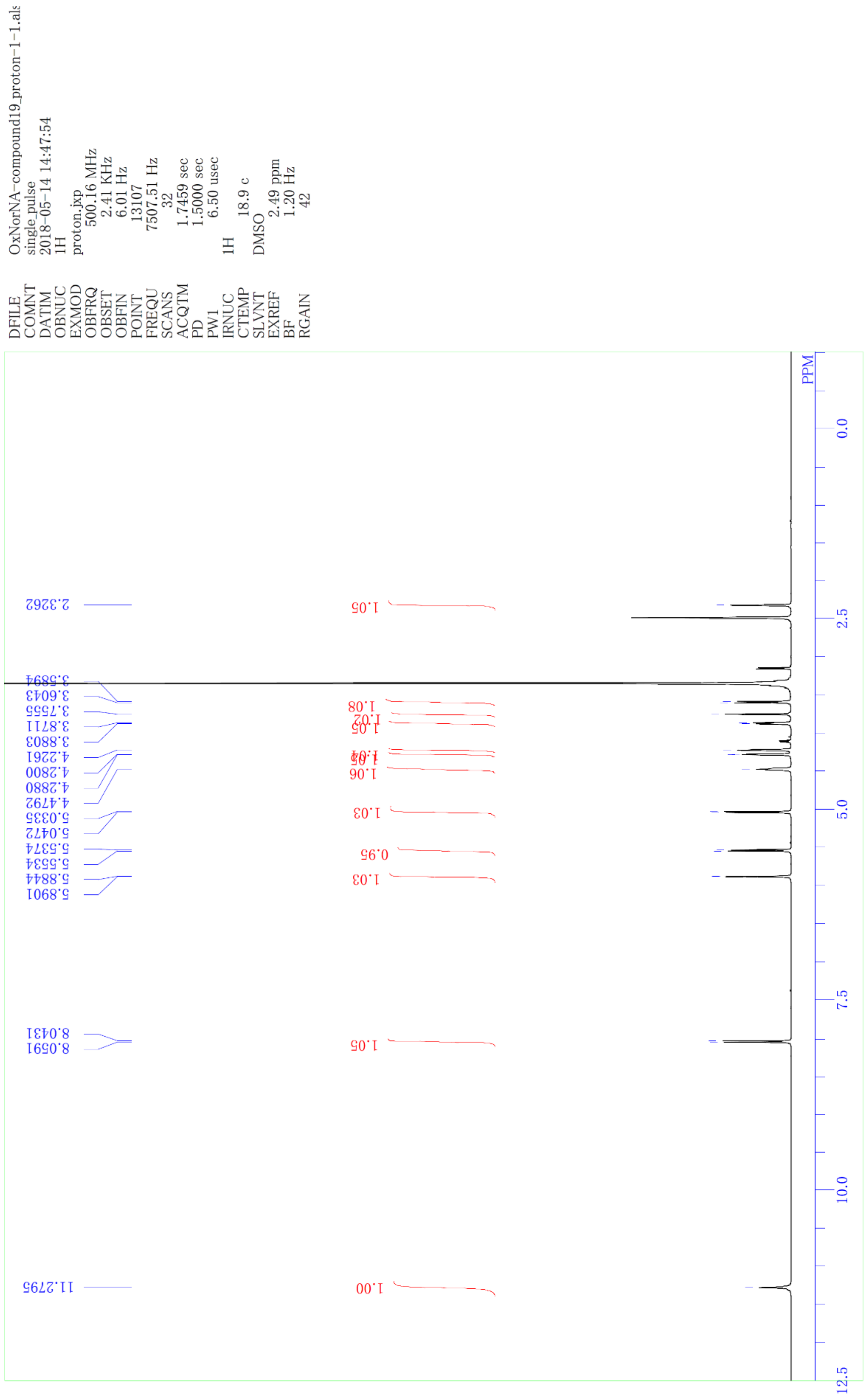

Compound **12** (^{13}C NMR, DMSO-d_6, 125MHz)

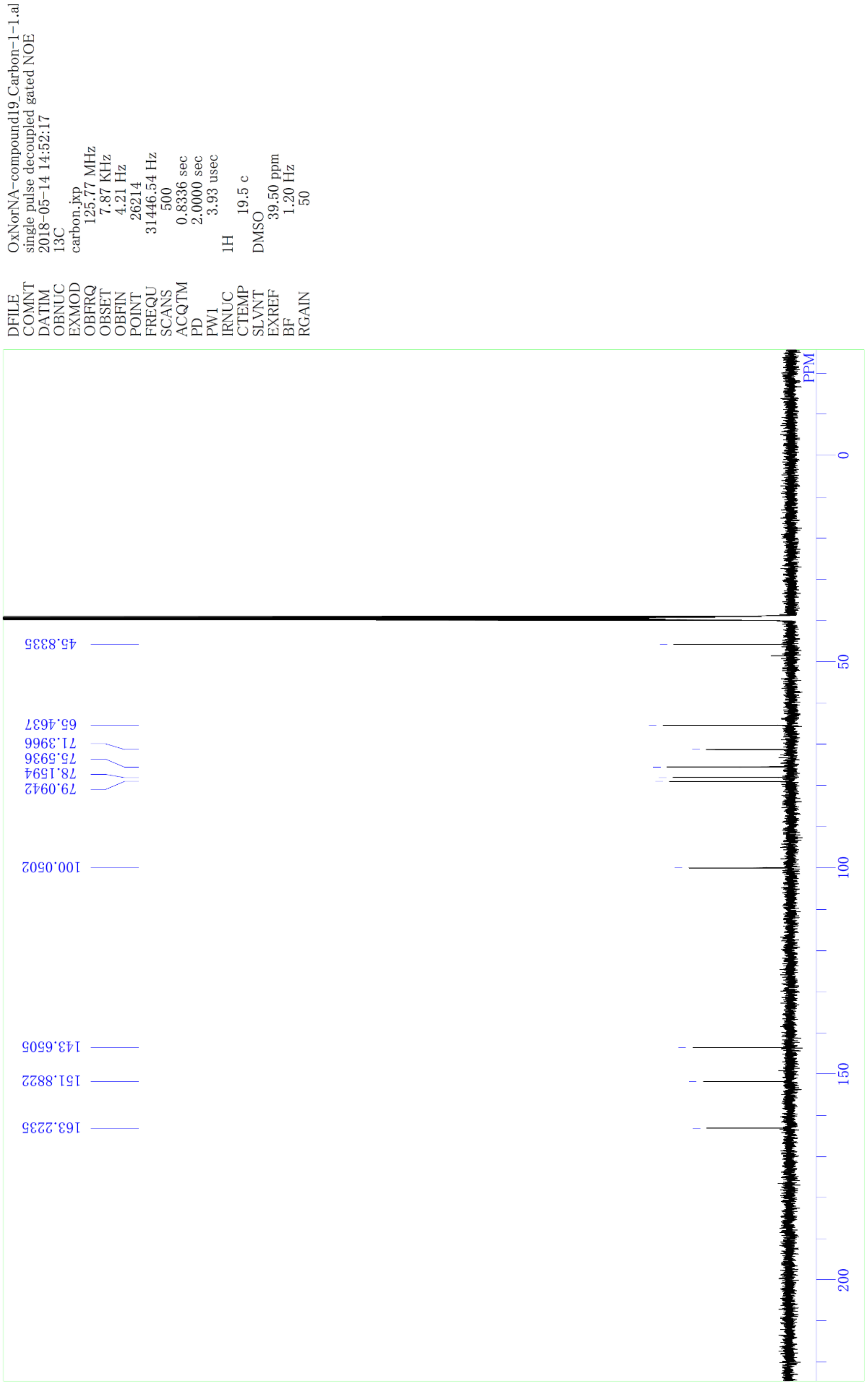

Compound **13** (^{1}H NMR, DMSO-d_6, 300MHz)

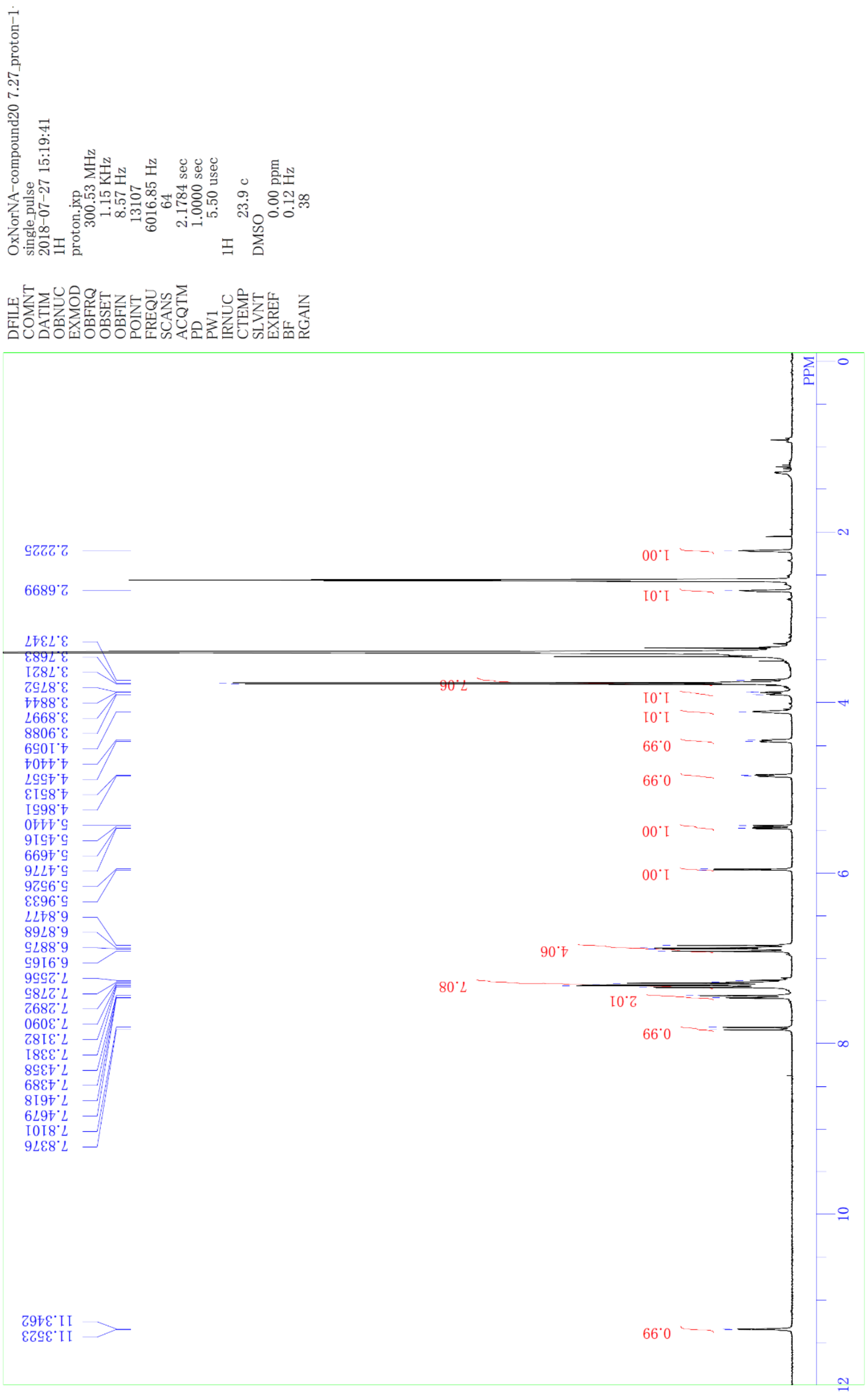

Compound **13** (^{13}C NMR, DMSO-d_6, 75MHz)

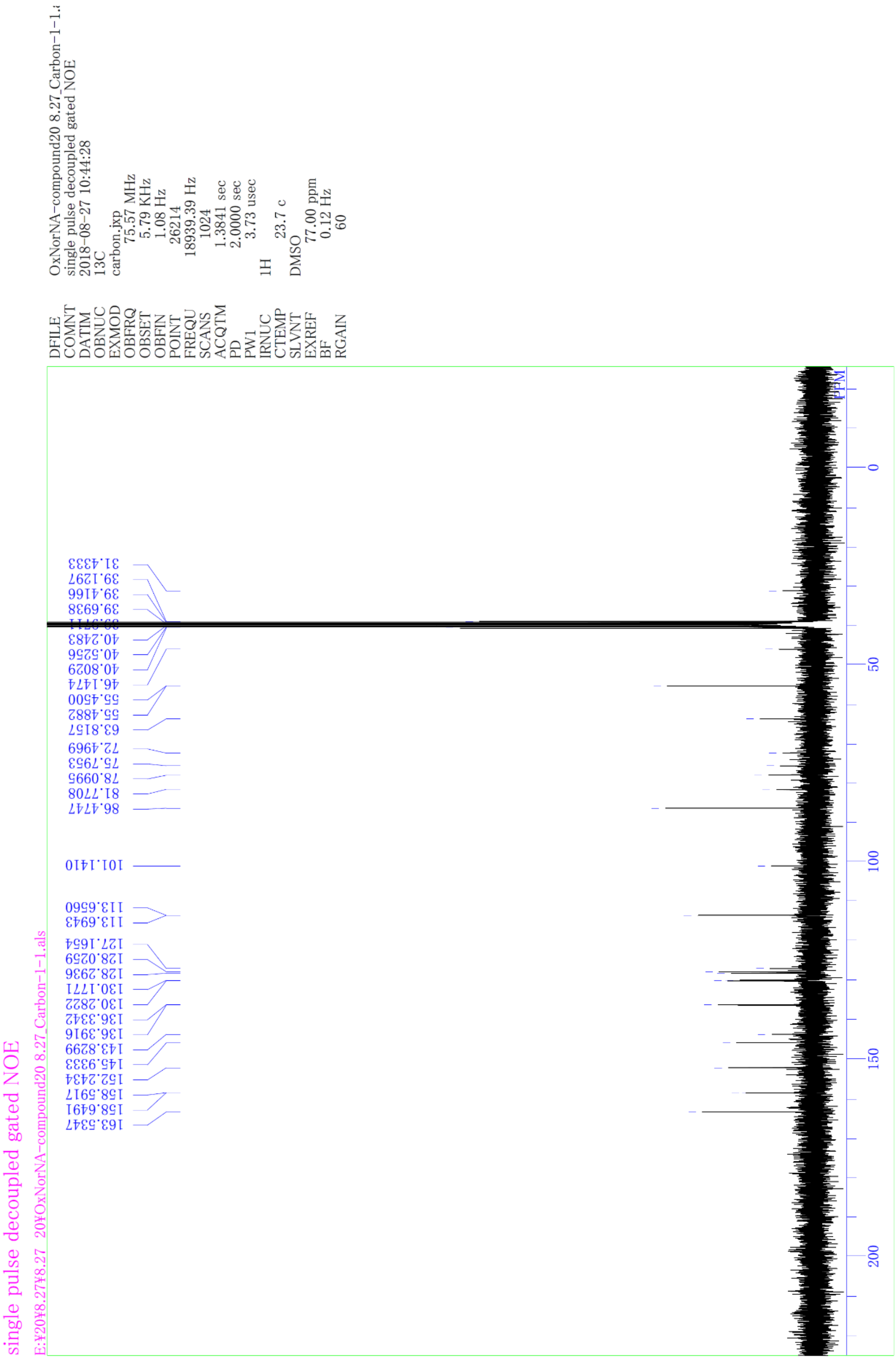

Compound **14** (^{1}H NMR, $CDCl_3$, 400MHz)

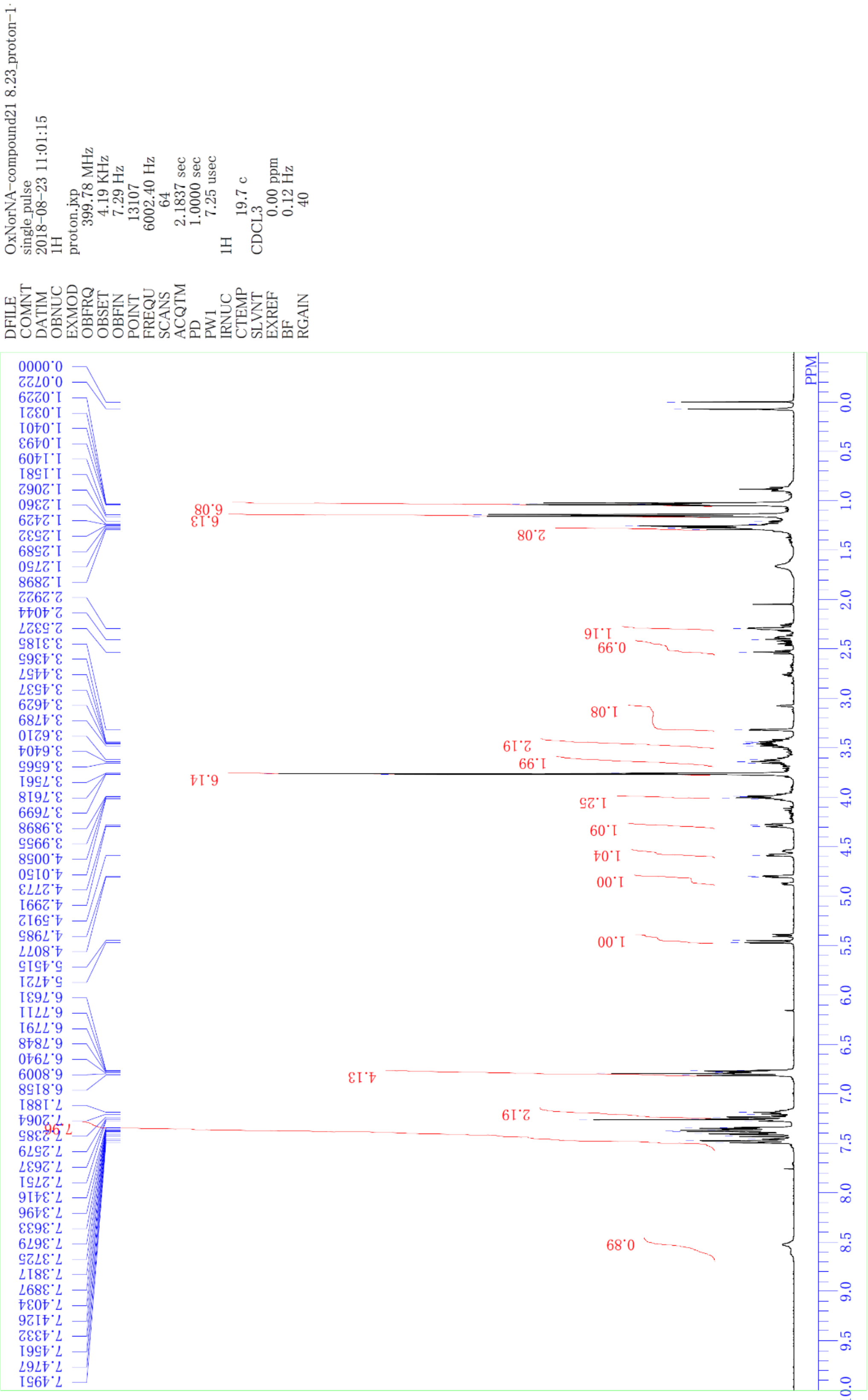

Compound **14** (^{13}C NMR, $CDCl_3$, 100MHz)

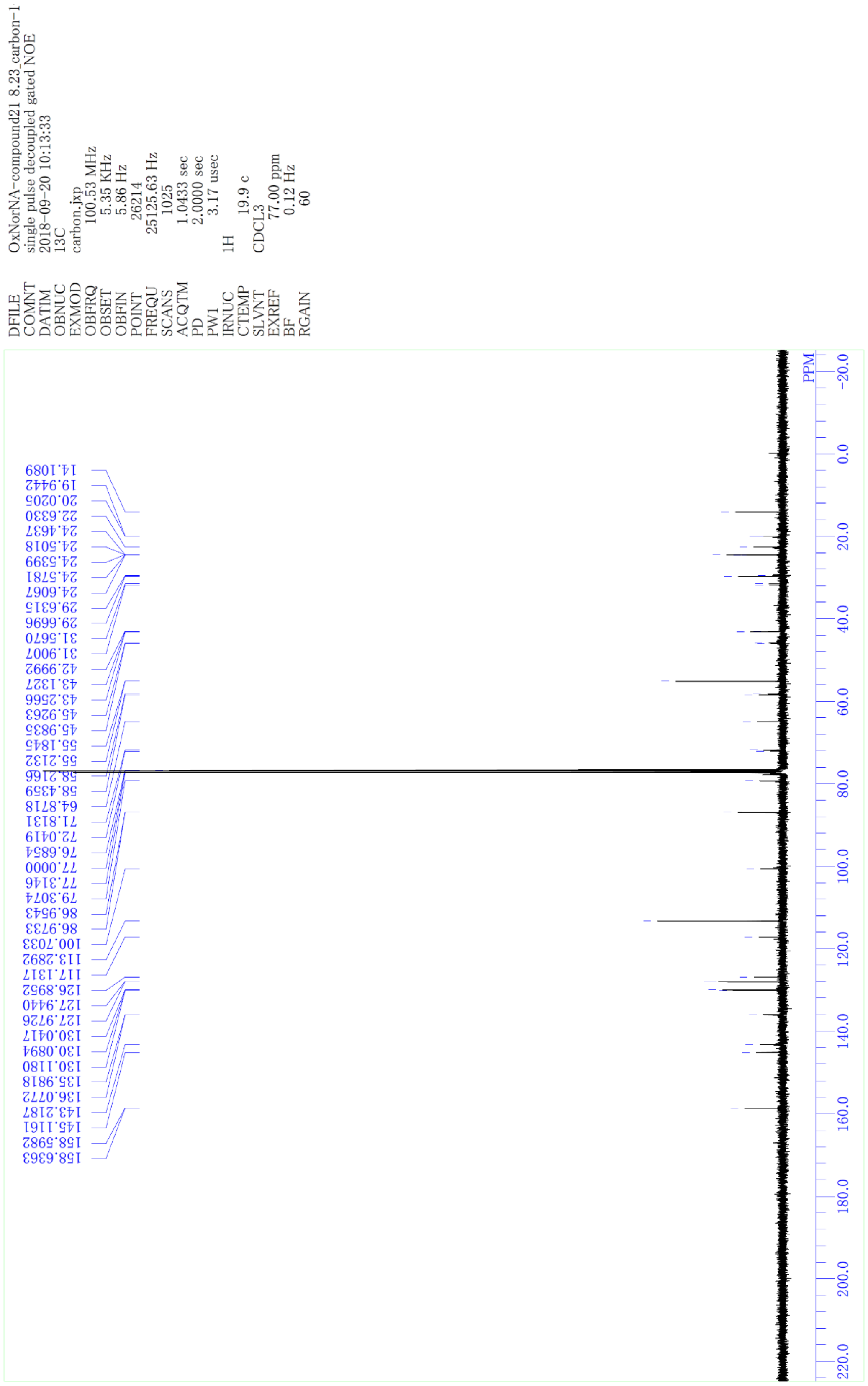

Compound **14** (^{31}P NMR, $CDCl_3$, 162MHz)

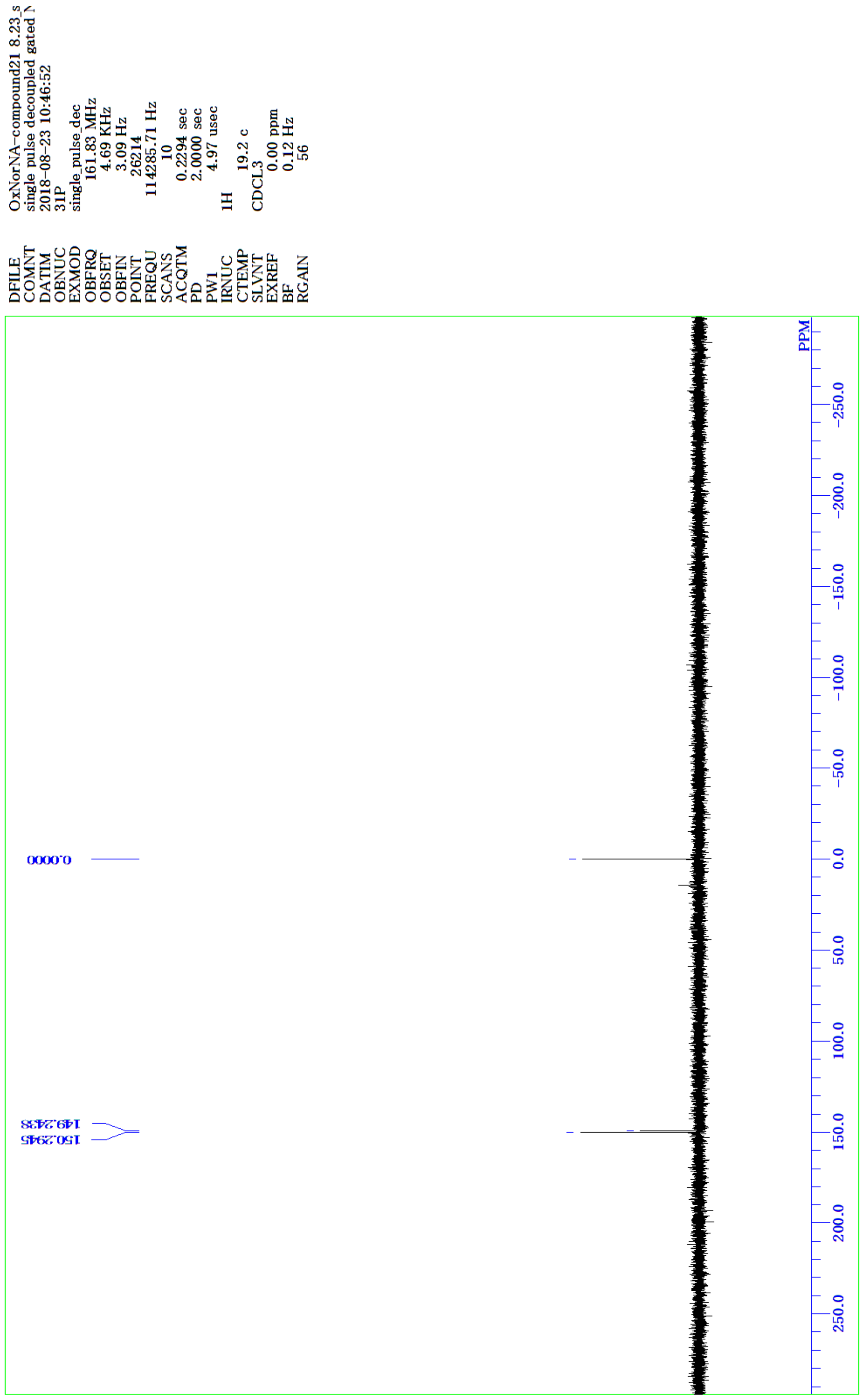

3. Copies of the HPLC and MALDI-TOF MS charts of the synthesized oligonucleotides

*Synthetic protocol for natural oligonucleotides (**ON4** and **ON5**)*

The natural DNA oligonucleotides (**ON4** and **ON5**) were synthesized on a 0.2 μmol scale by standard phosphoramidite chemistry (DMTr-off mode) utilizing an nS–8 Oligonucleotide Synthesizer (GeneDesign) and 2'-deoxyuridine phosphoramidite (**Table S3**). The resulting oligonucleotides were detached from the solid support by standard ammonia treatment (28% NH_4OH, rt, 4h), deprotected (28% NH_4OH, 55 °C, overnight), and purified quickly with NAP™-10 Sephadex™ G-25 DNA Grade columns and by reverse-phase HPLC.

Table S3. Isolated yields of natural oligonucleotides, together with MALDI-TOF MS data

	Sequence [a]	Yield (%)	$[M - H]^-$	
			Calcd	Found
**ON4**	5'-d(GCG TTU TTT GCT)-3'	74	3618.4	3617.9
**ON5**	5'-d(GCG UTU TUT GCT)-3'	66	3590.3	3589.9

HPLC (purified **ON1**) [eluent A: 0.1 M TEAA, eluent B: MeCN, gradient: 6–15% of eluent B]

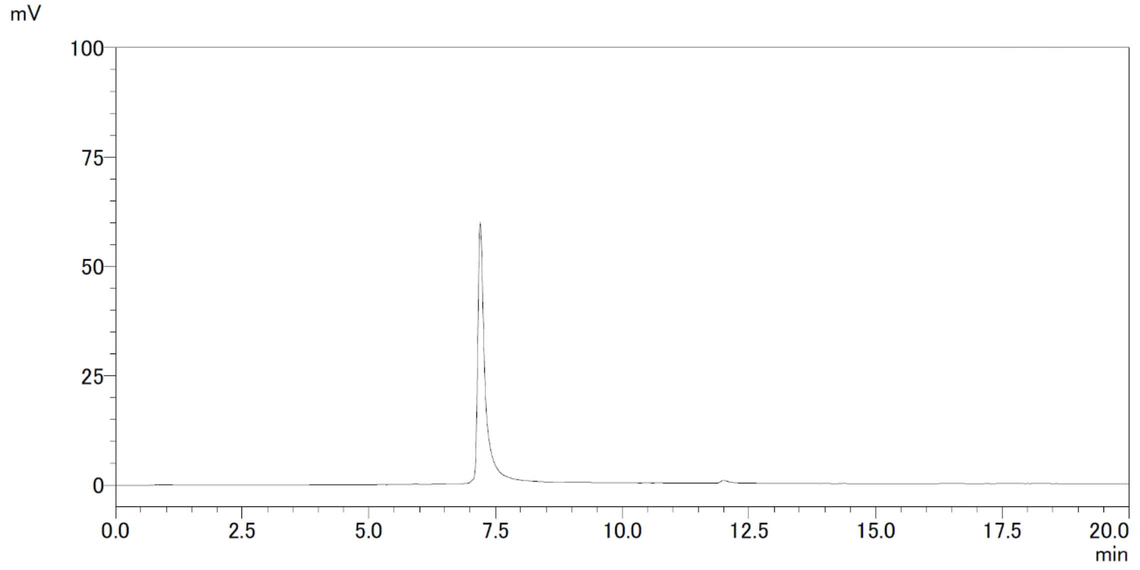

HPLC (crude **ON1** after Sep-Pak) [eluent A: 0.1 M TEAA, eluent B: MeCN, gradient: 6–15% of eluent B]

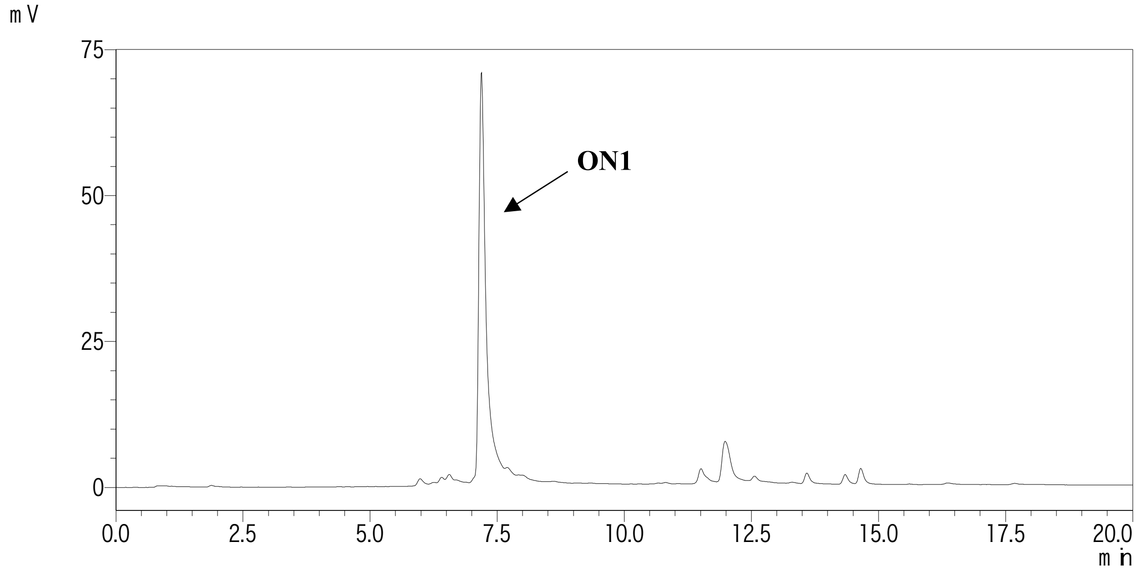

MALDI-TOF MS (purified **ON1**)

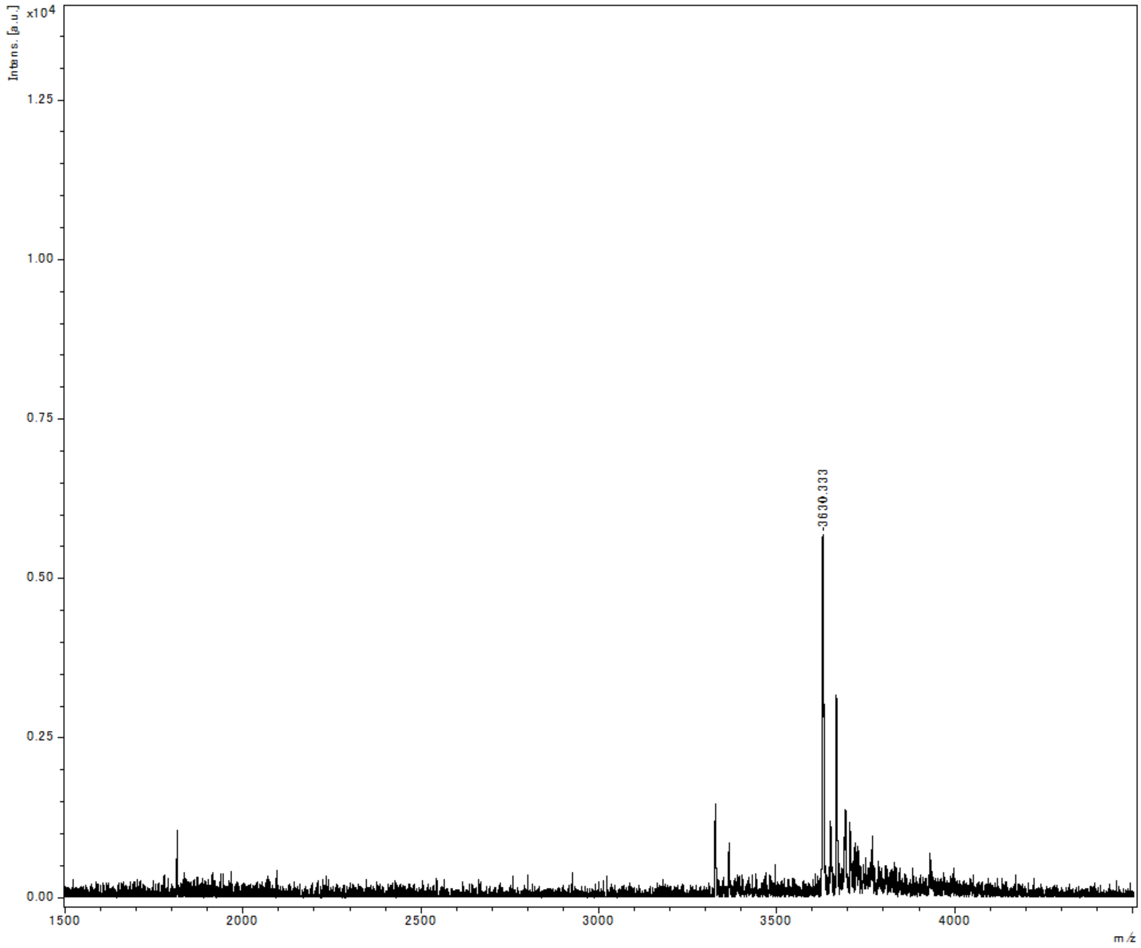

HPLC (purified **ON2**) [eluent A: 0.1 M TEAA, eluent B: MeCN, gradient: 5–9% of eluent B]

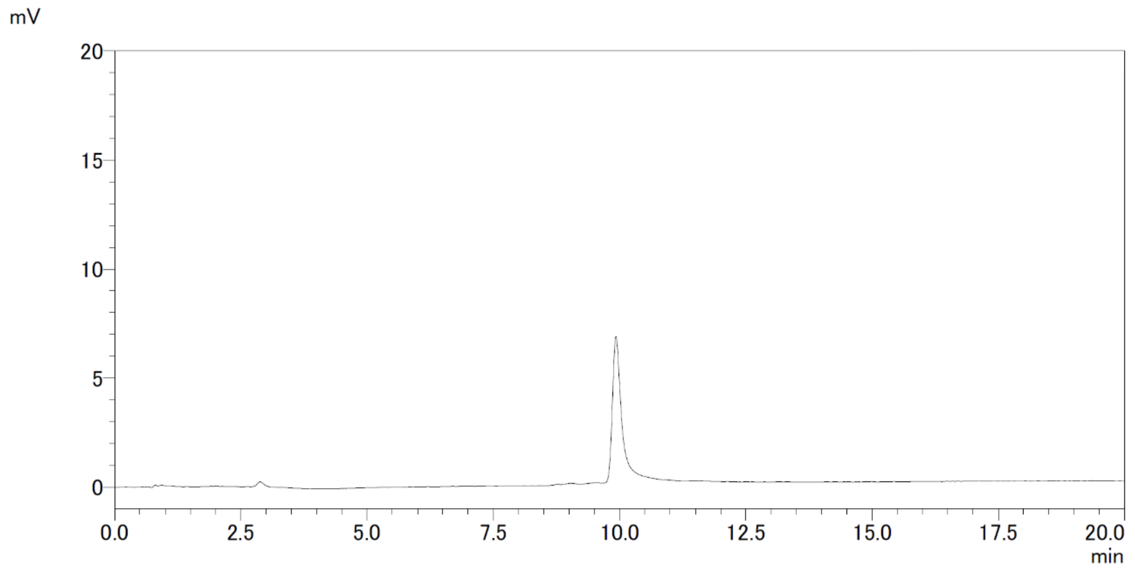

HPLC (crude **ON2** after Sep-Pak) [eluent A: 0.1 M TEAA, eluent B: MeCN, gradient: 6–15% of eluent B]

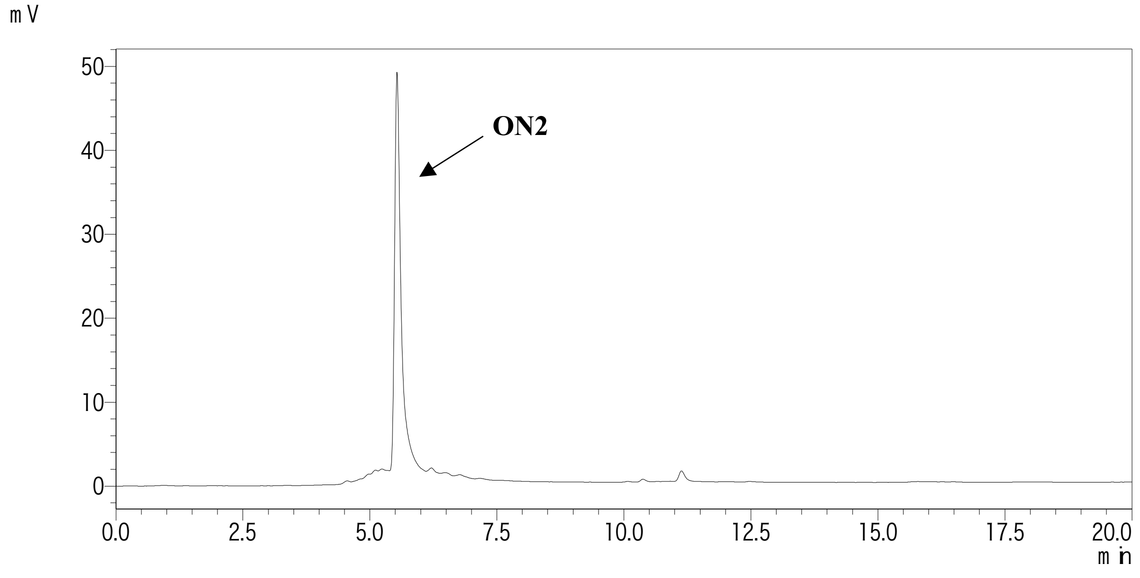

MALDI-TOF MS (purified **ON2**)

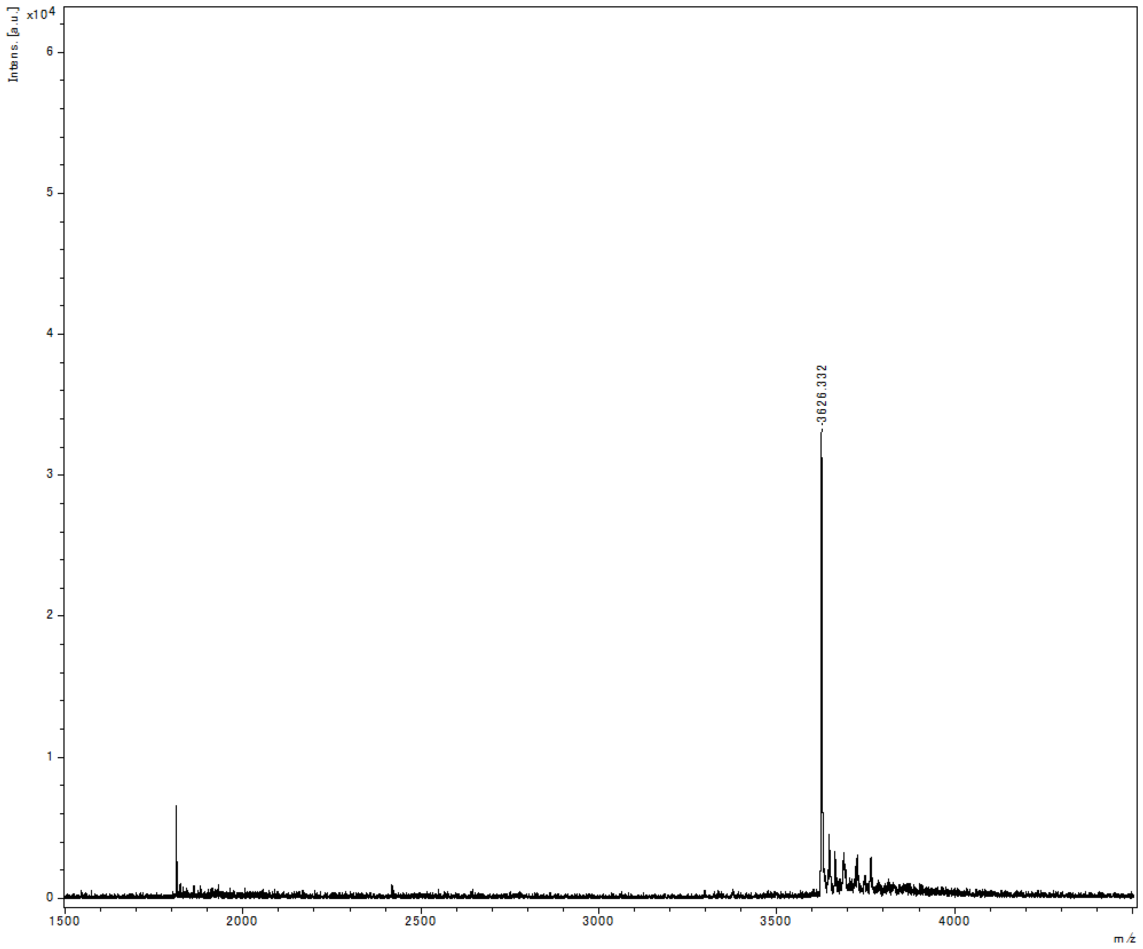

HPLC (purified **ON3**) [eluent A: 0.1 M TEAA, eluent B: MeCN, gradient: 6–15% of eluent B]

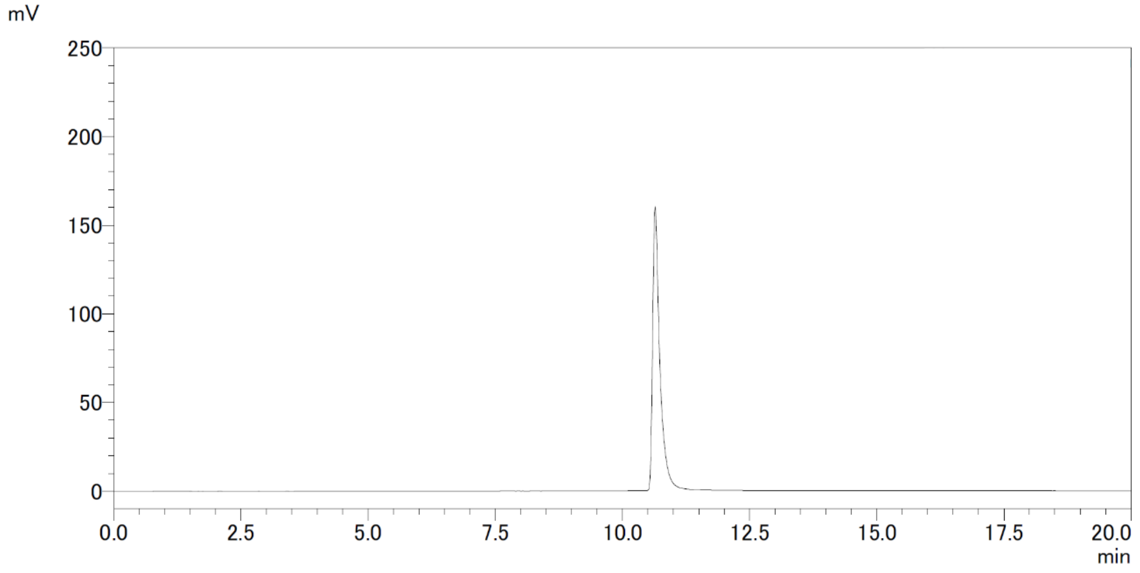

HPLC (crude **ON3** after Sep-Pak) [eluent A: 0.1 M TEAA, eluent B: MeCN, gradient: 6–15% of eluent B]

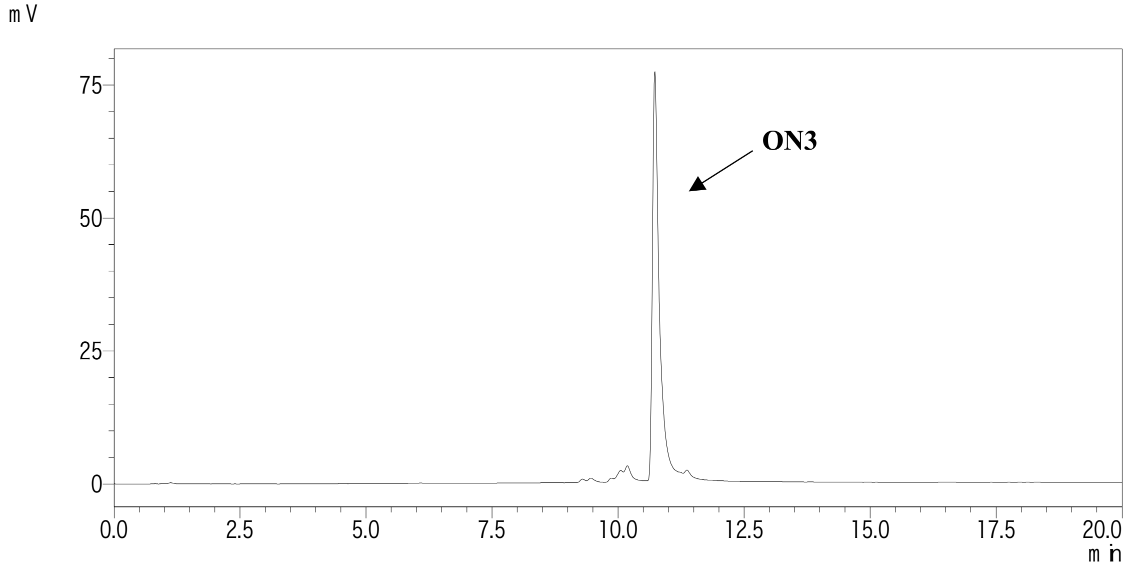

MALDI-TOF MS (purified **ON3**)

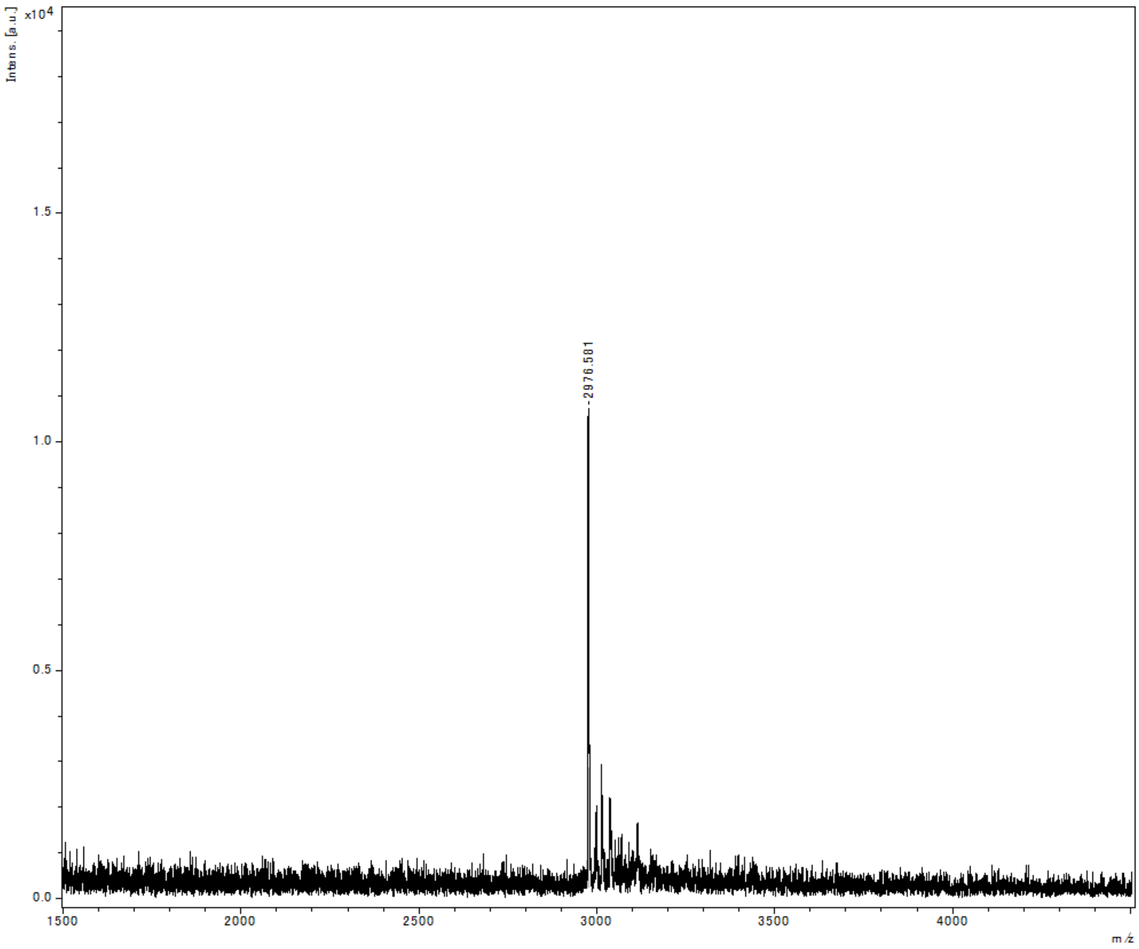

HPLC (purified **ON4**) [eluent A: 0.1 M TEAA, eluent B: MeCN, gradient: 6–15% of eluent B]

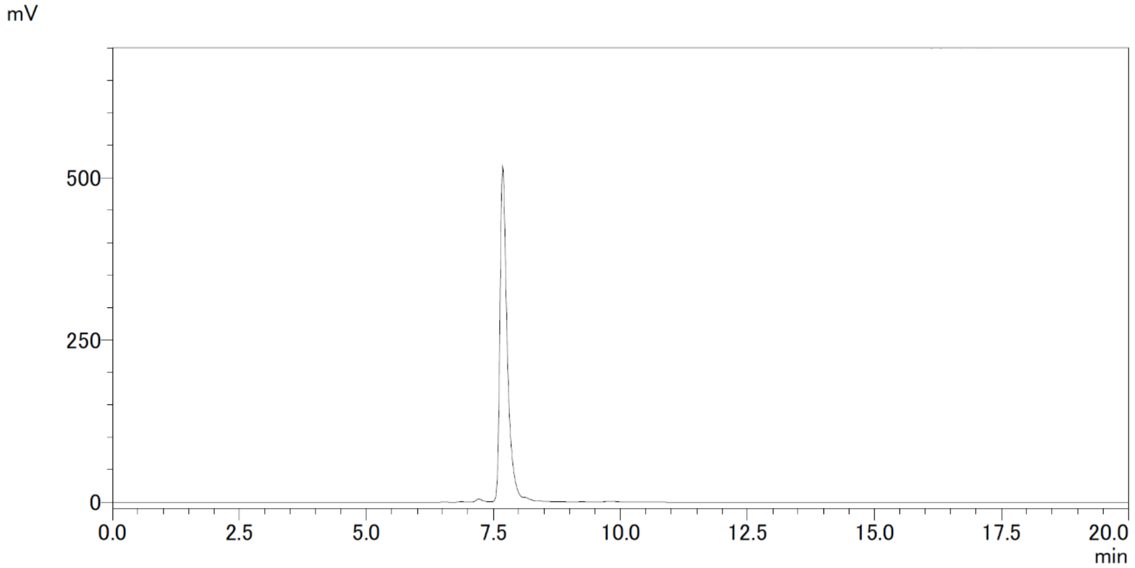

MALDI-TOF MS (purified **ON4**)

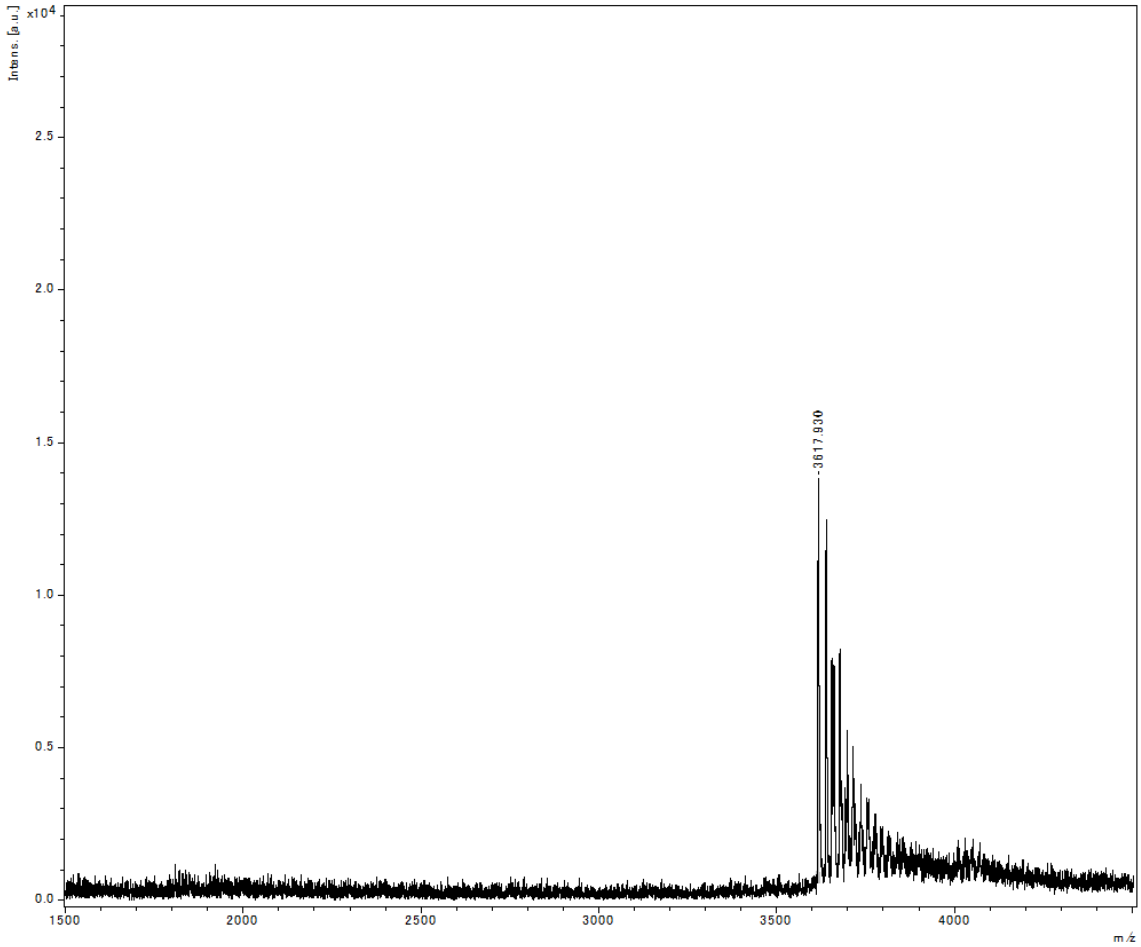

HPLC (purified **ON5**) [eluent A: 0.1 M TEAA, eluent B: MeCN, gradient: 6–15% of eluent B]

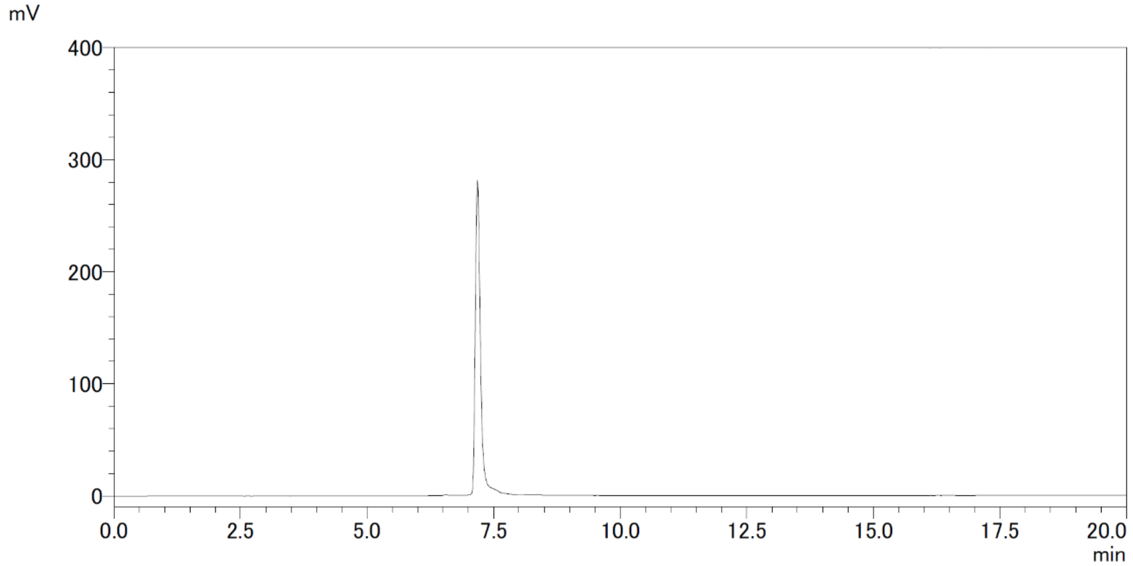

MALDI-TOF MS (purified **ON5**)

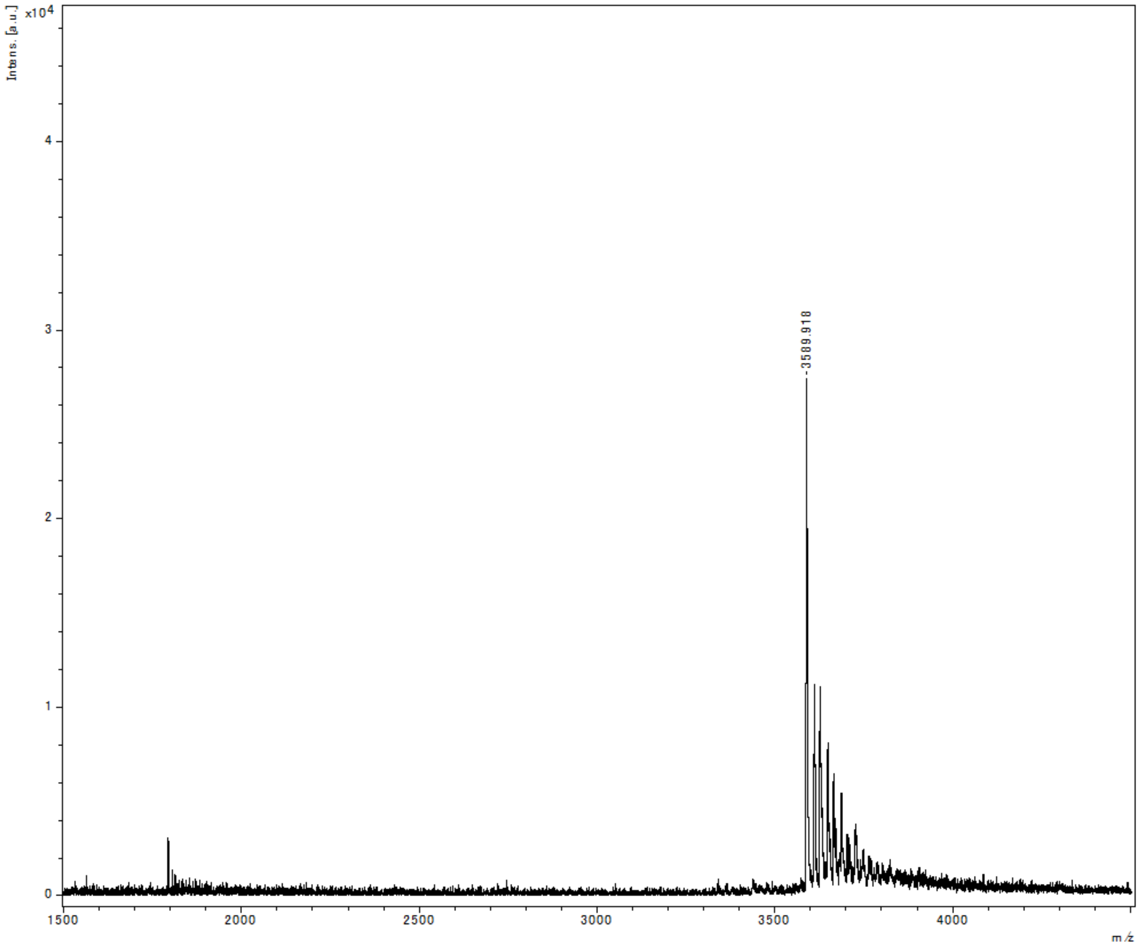

Supplement: Supplementary file 1 [file molecules-25-01732-s001.pdf]
